# A systematic review of ultrasound to predict severe disease in patients with dengue and other viral infections associated with hemorrhagic fevers

**DOI:** 10.1371/journal.pntd.0014609

**Published:** 2026-09-17

**Authors:** Daniel Z. Hodson, Renato A. Tambelli, Mahathar A. Wahab, Claudia Wallrauch, Hans-Michael Steffen, Benno Kreuels, Sabine Bélard, Tom Heller

**Affiliations:** 1 Division of Internal Medicine – Pediatrics, David Geffen School of Medicine at the University of California (Los Angeles), Los Angeles, United States of America; 2 Department of Emergency Medicine, Hospital das Clínicas da Faculdade de Medicina de Marília, São Paulo, Brazil; 3 Emergency and Trauma Department, Hospital Kuala Lumpur, Kuala Lumpur, Malaysia; 4 Ministry of Health, Putrajaya, Malaysia; 5 Lighthouse Clinic Trust, Lilongwe, Malawi; 6 Institute of Infectious Diseases and Tropical Medicine, LMU University Hospital, Munich, Germany; 7 Department of Gastroenterology and Hepatology, Faculty of Medicine, University Hospital Cologne, University of Cologne, Cologne, Germany; 8 Hypertension Center, Faculty of Medicine, University Hospital Cologne, University of Cologne, Cologne, Germany; 9 Department of Postgraduate Studies and Research, Chreso University, Lusaka, Zambia; 10 Division for Tropical Medicine, Department of Medicine, University Medical Centre Hamburg-Eppendorf, Hamburg, Germany; 11 Research Group Neglected Diseases and Envenoming, Section for Implementation Research, Bernhard Nocht Institute for Tropical Medicine, Hamburg, Germany; 12 Department of Medicine, School of Medicine, Kamuzu University of Health Sciences, Blantyre, Malawi; 13 Institute of Tropical Medicine, University of Tübingen, Tübingen, Germany; 14 German Center for Infectious Disease Research, DZIF Partner Site Tübingen, Tübingen, Germany; Beijing Children’s Hospital Capital Medical University, CHINA

## Abstract

**Background:**

Viral infections associated with hemorrhagic fevers (VaHFs) cause millions of cases each year across a broad geography with severity ranging from asymptomatic cases to clinical disease with a high case fatality rate. Commonalities in pathophysiology across VaHFs include vascular damage and plasma leakage with the possibility of hemorrhage, organ failure, shock, and death. These changes are seen on autopsy, and evidence of plasma leakage can be readily detected on ultrasound.

**Methods:**

We conducted a systematic review to assess ultrasound findings in VaHFs and their ability to predict severe disease (PROSPERO CRD42024620862). PubMed was searched through May 2025. Any articles presenting original, patient-level findings on ultrasound in VaHFs with serologic and/or molecular confirmation were included. Case series and duplicative datasets were excluded. A QUADAS-2 tool was used to assess bias. Study level characteristics and patient-level data on the ultrasound findings were extracted. The crude proportions of ultrasound findings were reported, and weighted meta-analysis using a random-effects model was performed and displayed in forest plots when the data allowed. Sensitivity, specificity, and positive/negative likelihood ratios for severe disease were calculated and visualized using a random effects model in SROC plots when the data allowed.

**Results:**

In total, 457 unique publications were screened for inclusion, and 56 publications comprising 6979 participants were included in the review, including 44 publications (6189 participants) on dengue, six (494 participants) on Crimean-Congo hemorrhagic fever, three (134 participants) on hantavirus disease, two (116 participants) on yellow fever, and one (46 participants) on Lassa fever. Participants were most often recruited while acutely sick during periods of high transmission at tertiary care centers. Ultrasound findings included gallbladder wall thickening, ascites, pleural effusions and abnormal lung ultrasound, pericardial effusions, abdominal lymphadenopathy, splenomegaly, abnormal pancreatic ultrasound, and hyperechoic kidneys or liver. Pericardial effusions and several other findings were associated with severe disease.

**Conclusions:**

Common pathophysiology leading to plasma leakage results in sonographic signs across viral infections associated with hemorrhagic fevers, and some findings can help predict severe disease. Important limitations included reliance on a single database and possibility of missing manuscripts from local journals, the small number of publications on non-dengue VaHFs, patient recruitment overwhelmingly from the in-hospital setting at tertiary referral centers during high-transmission periods, high degree of heterogeneity, and bias in the timing domain among the studies on dengue. We first propose ultrasound findings be incorporated into new definitions of severity for VaHFs and then introduce an Ultrasound-based assessment of Plasma leakage Severity (UPS) as a hypothesis generating framework to help guide future, prospective research. We discuss some virus and organ-specific considerations and conclude with detailed considerations for future research.

## Author summary

Viral infections associated with hemorrhagic fevers (VaHFs) may lead to vascular damage and plasma leakage with the possibility of hemorrhage, organ failure, shock, and death. Plasma leakage can be readily detected on ultrasound, and in this review, the authors assess the frequency of gallbladder wall thickening, ascites, pleural effusions and abnormal lung ultrasound, pericardial effusions, abdominal lymphadenopathy, splenomegaly, abnormal pancreatic ultrasound, and hyperechoic kidneys or liver in VaHFs, as well as the ability of these findings to predict severe disease. The review was dominated by studies on dengue, and there was great heterogeneity in the frequency of ultrasound findings in part related to variability in the era the study was completed, the age of participants, and the location of the original study. Several findings were associated with severe disease, and the authors raise many important considerations for future research. The authors recommend ultrasound findings be incorporated into definitions of severity for VaHFs and then propose an ultrasound-based grading scale for plasma leakage severity (UPS) as a hypothesis generating framework to help spark future research.

## Introduction

Viral infections associated with hemorrhagic fevers (VaHFs) result from the families Flaviviridae, Bunyaviridae, Arenaviridae, and Filoviridae [1,2]. Primates, including humans, serve as reservoirs for some of the implicated viruses (most notably yellow fever and dengue), while many other viruses reside in livestock, rodents, or bats [2–4]. The yellow fever and dengue viruses require insect vectors for transmission, while others spread directly from insects/animals to humans or even between humans [5,6]. VaHFs are geographically widespread with annual case numbers ranging from dozens to millions and can be associated with high morbidity and mortality [1]. The epidemiology and case burden reflect both environmental variables (such as rainfall) and human practices (such as farming, deforestation, and travel).

While many cases remain subclinical, dengue viruses, Crimean Congo hemorrhagic fever (CCHF) virus, hantaviruses, Lassa fever virus, and yellow fever virus all have the potential to lead to severe disease in humans. Dengue symptoms classically begin within 1–2 weeks of infection and progress through three stages: the febrile stage, the critical phase, and the recovery phase [3,7]. If they occur, severe manifestations occur during the critical phase in which a combination of plasma leakage, gastrointestinal loses, inadequate oral intake, and hemorrhage can contribute to hemodynamic instability and shock [3,7]. Severe CCHF is characterized by refractory shock, severe coagulopathy, effusions, multifocal necrosis of the liver and other organs, and bleeding, primarily from the nose, vagina, and gastrointestinal tract [8]. Death typically occurs within two weeks after symptom onset, [9,10] emphasizing the importance of establishing prognosis within the first 3–5 days of illness for optimal management [8]. Hantavirus syndromes depend on the local virus: The Hantaan and Seoul strains of China and Korea [11] as well as the Dobrava-Belgrade virus in southern Europe [12] may lead to kidney failure and shock associated with hemorrhagic fever with renal syndrome, which classically exhibits five phases (febrile, hypotensive, oliguric, polyuric, and convalescent) [13]. Among new world hantaviruses, the Sin Nombre virus can manifest as hantavirus pulmonary syndrome [14] as well as acute infection without significant cardiopulmonary symptoms [15]. In contrast, the Puumala virus, predominantly found in Scandinavia and parts of Russia, typically causes a milder clinical illness known as nephropathia epidemica characterized by headache, abdominal pain, emesis/diarrhea, and vision changes; [16] while less common, severe disease may include oligouria, hypotension, and shock [13]. Lassa fever classically progresses through four stages: general malaise and high fever within the first three days; exudative pharyngitis, productive cough, conjunctivitis, body aches, gastrointestinal distress with anemia, proteinuria, and hypotension in the second half of the first week; mucosal and internal hemorrhage, facial edema, altered mental status, seizures in the second week; coma and death in fulminant Lassa fever occurs after two weeks [17]. Among the pediatric population, Lassa fever may also present as congenital infection from an infected mother or a “swollen baby syndrome” with anasarca, abdominal distension, and hemorrhage [18]. Finally, severe yellow fever classically presents as acute hepatitis (with the notable jaundice giving the virus and disease its name), hemorrhage, renal failure, shock, and death [5].

Despite their different manifestations of severe disease, key aspects of the clinical presentation of VaHFs that bring patients to the health system include febrile illness and vascular dysfunction [2]. Autopsy studies have concluded generalized vascular damage “constitutes the basic disease process” and “the most important change in the disease” [19,20]. As detailed above, the disease course varies depending on the specific virus, but common initial symptoms are nonspecific and include fever, myalgia, and fatigue; while in more severe cases, vascular leakage, hemoconcentration, organ-specific damage, and shock may develop [21,22]. Multifocal bleeding, disseminated intravascular coagulation, encephalopathy, and severe shock are associated with poor outcomes [21]. Autopsy studies demonstrate edema, effusions, hemorrhage, and necrosis [19,20,23,24]. Treatment mainly consists of supportive care, particularly fluid and electrolyte management, [1] although specific agents may be employed, such as ribavirin for Lassa fever or Crimean-Congo hemorrhagic fever and monoclonal antibodies for Ebola virus disease and VaHFs caused by new world arenaviruses [6,25–28].

The application of ultrasound to the management of tropical infections continues to gain momentum in under-resourced settings [29]. In addition, simplified bedside or point-of-care ultrasound (POCUS) protocols, such the Focused Assessment with Sonography for HIV-associated tuberculosis (FASH) to detect findings suggestive of extrapulmonary tuberculosis, can expedite decision making in resource limited settings [30,31]. In the context of VaHFs, there are three key areas where ultrasound can contribute: First, detecting sonographic signs may increase the likelihood of diagnosis of viral infection, particularly in an epidemic setting where the pretest probability is already high. In such scenarios, even nonspecific findings such as effusions or gallbladder wall thickening (GBWT) may provide diagnostic support. However, other rapid point-of-care tests, such as lateral flow immunoassays for the dengue nonstructural 1 protein (NS1) with specificities in the high 90%, [32] likely outperform ultrasound in terms of diagnostic accuracy for VaHFs. Thus, the greatest utility of ultrasound in VaHFs may not be for diagnosis, [33] and any potential diagnostic value of ultrasound in VaHFs was not the focus of this review.

Second, ultrasound can be instrumental in guiding fluid management and determining the category of shock (i.e., hypovolemic, distributive, obstructive, cardiogenic) in VaHF patients, analogous to general shock protocols such as the Rapid Ultrasound in Shock (RUSH) protocol [34]. Third, ultrasound can guide the removal of fluid from the peritoneal, pleural, and pericardial spaces. While important, these uses are not specific to VaHFs, so they were not the focus of this review.

Fourth, and the primary aim of this review, ultrasound can assist in grading disease severity and in predicting the clinical course. In this way, ultrasound could influence triage and management decisions, such as the determination of inpatient versus outpatient care, the decision for intravenous versus oral fluid replacement, and the need for admission to a unit capable of caring for critically ill patients.

Modeled after the aforementioned FASH, ultrasound protocols such as the Focused Assessment with Sonography for Lassa (FAS-La) and Focused Assessment with Sonography for Dengue (FAS-D) have already been developed [35,36]. While substantial literature exists on the use of ultrasound in dengue, research on ultrasound applications in other VaHFs has been much less common, and lessons from one VaHF may be relevant for other diseases. This article systematically reviews the current evidence regarding ultrasound findings in viral infections associated with hemorrhagic fevers and the potential of sonographic signs to predict disease severity. From this review and our clinical experience, we then 1) propose that ultrasound findings be incorporated into definitions of disease severity and 2) introduce an ultrasound-based grading system for disease severity intended to be combined with other clinical, laboratory, and imaging findings. We conclude with some virus and organ-specific considerations, key limitations, and detailed considerations for future research.

## Methods

### Study design and registration

We conducted a systematic review of primary studies to assess the frequency of ultrasound findings in VaHFs and their ability to predict disease severity. The initial protocol of the systematic review was registered in PROSPERO (CRD42024620862) [37].

### Search strategy

Infections due to the following viruses were considered: dengue virus, Lassa virus, Crimean-Congo hemorrhagic fever (CCHF) virus, Ebola virus, Marburg virus, yellow fever virus, hantaviruses, Rift Valley fever virus, Junin virus, Machupo virus, Sabia virus and Guanarito virus. A search string capturing linguistic differences for both ultrasound and virus names was used (S1 Text); no restrictions were placed on article type, publication date, or language.

### Study selection, inclusion, and exclusion

PubMed was searched through May 17, 2025. Citations were imported into Rayyan (Cambridge, USA) for screening, and then citations and publications were imported into Zotero 6.0 (Corporation for Digital Scholarship, Vienna, USA) for full text review. Any articles presenting original, patient-level findings on ultrasound examinations in infections due to any of the above-mentioned viruses for which there was confirmation from serologic and/or nucleic acid testing were included. Echocardiography-based studies were included and reviewed only for data on pericardial effusions. The following types of publications were excluded: studies in which only a subset of enrolled participants were specifically selected for ultrasound examination, studies using the same set of patients as an already included publication, review articles, studies reporting only on ocular findings, and opinion or perspective pieces. In a second step, case studies or case series of three or fewer patients were removed. Two authors (TH, DZH) independently screened titles, abstracts, and the full texts of publications for inclusion, and any discrepancies were resolved through discussion and consensus.

### Data extraction

The following study-level data were extracted from the included publications by one author (DZH): age range of patients (children, adolescent, adult, mixed or unspecified), country of origin, recruitment setting (inpatient, emergency department, outpatient), recruitment methodology (prospective vs retrospective, consecutive vs convenience), the type of provider who performed the ultrasound scans (radiologist, cardiologist, sonographer, general physician or clinician), the percent of confirmed cases that underwent ultrasound, and the definition of severe disease if applicable. Patient level data on the presence of ultrasound findings were initially extracted by one author (TH) and included effusions in the pericardial, pleural, or abdominal cavities; GBWT; B-line pattern of the lungs; abdominal lymphadenopathy; and changes of the kidneys, liver, spleen, or pancreas. We did not extract data on hepatomegaly. A second author (DZH) then double-checked patient level data-extraction from a subset of publications, and any discrepancies were resolved through review of the original publication, discussion, and consensus. The cutoff values for GBWT and splenomegaly were also extracted when available (DZH). When ultrasound findings were reported by disease severity in the original publication, the number of participants with each finding was extracted by categorization of disease severity and the publication was included as a “predictive” study. If patient level data was not available by severity, the publication was included as a “descriptive” study only.

### Risk of bias assessment

Risk of bias was initially assessed by one author (TH) using an adapted QUADAS-2 tool for reviews of diagnostic accuracy (S2 Text) [38]. Bias and applicability were assessed across four domains: patient selection, the use of ultrasound as the index test, the definition of severity as the reference standard, and timing of the ultrasound assessment. As noted above, studies which did not provide serologic confirmation of infection or specifically scanned only a subset of patients were already excluded. Bias in patient selection was noted if >10% of cases were not confirmed or >10% of cases did not receive an ultrasound scan, and this domain was double-checked by a second author (DZH). Bias in the timing of the ultrasound assessment was noted if the ultrasound scan was done concurrently with the determination of disease severity, after the severity of disease had been determined, or if the timing of the ultrasound examination in relation to the determination of disease severity was unclear.

### Statistical analysis

Data were extracted and organized in Google sheets (Alphabet Inc., Mountain View, USA) and Microsoft Excel (IBM Corp., Armonk, USA). The frequencies of ultrasound findings with confidence intervals were first tabulated in Microsoft Excel as crude proportions for all findings. When there were at least three available studies, a weighted meta-analysis of the frequency of ultrasound findings was then performed in SPSS Version 31 (IBM Corp., Armonk, USA) using a random-effects model with a continuous effect size (the proportion of participants with the ultrasound finding) and displayed in forest plots. When the frequency was zero or the effect size was zero or one, a constant continuity correction of 0.01 was applied to allow for visualization and analysis. Three types of subgroups were considered: the era of publication year (≤ 2009, 2010 – 2019, ≥ 2020), age group of participants in the publication (pediatric + /- adolescent, adult + /- adolescent, mixed/not specified), and the region in which the study was conducted. For dengue, the regions were defined as South America, the Indian subcontinent including India/Pakistan/Sri Lanka, and East Asia including all other countries in Asia. For CCHF, the studies available for formal analysis were only from two countries, Turkey or Iran. For other VaHFs, formal meta-analysis was not possible. Heterogeneity of the frequency of ultrasound findings was assessed using the I² statistic. The effect of subgroups on heterogeneity was assessed in two ways: 1) examination of the I^2^ for each subgroup, and 2) the test of between-subgroup homogeneity with the Q statistic. To highlight the effect of age on the frequency of findings, the crude proportion of patients with each finding was additionally compared between pediatric/adolescent patients and adult/adolescent patients for dengue using a publicly available online calculator (https://www.statology.org/two-proportion-z-test-calculator/) for two-sided two-proportion z-tests. Statistical significance was defined as p < 0.05.

Severity was defined according to the categorization used in each study. Studies about dengue most often used one of the iterations of the WHO definition (S3 Text). Depending on the iteration, infection with dengue may be classified as dengue fever (DF), dengue hemorrhagic fever (DHF) with Grades 1–4, dengue shock syndrome (DSS), dengue with warning signs (DWS), or severe dengue disease (SDD) [3,39–41]. The treatment arm continuity correction (TACC) was applied when crude data showed a cell value of zero, and the corrected values were reported [42,43]. In the TACC, a different value is added to cells within the experiment group (here the group with positive ultrasound sign) and the control group (here the group with a negative ultrasound sign). The correction added to the group with a positive ultrasound finding equals the number of participants with positive ultrasound sign divided by the total number of participants, while the correction added to the group with a negative ultrasound finding equals the number of participants with negative ultrasound divided by the total number of participants. Due to concern for bias in the timing domain and definition of severity among the publications on dengue, only studies with low risk of bias in these domains were included in the analyses on the prediction of severe disease, and the small number of studies available limited the statistical analyses that could be reasonably performed.

First, the crude sensitivity, specificity, and positive/negative likelihood ratios, along with the 95% confidence intervals, of the ultrasound finding for severe disease were calculated from 2 x 2 tables using a publicly available online calculator (https://www.medcalc.org/en/calc/diagnostic_test.php) [44]. These test characteristics were selected because they remain independent of population prevalence, which could not be known from the included data. Likelihood ratios (LR) were then interpreted according to McGee in which a significant LR was defined as one which changes the probability by ≥ 20% [45]. A clinically significant positive LR was therefore defined as ≥ 3.0 (which increases the probability by ≥20%), while a clinically significant negative LR was defined as ≤ 0.4 (which decreases the probability by ≥20%) [45]. Second, the sensitivity and specificity of all findings were visualized for each VaHF using a random effects model in SROC plots using MetaDTA (https://crsu-metadta.le.ac.uk/MetaDTA/), a publicly available web-based platform for meta-analysis of diagnostic accuracy data based on the R packages Ime and Shiny [46,47]. Due to the small number of studies available and numerous variables (type of ultrasound finding, age of participants, country/region, year/period, cutoff value for GBWT and splenomegaly), it was not possible to perform subgroup or sensitivity analyses.

## Results

### Characteristics of included studies

**Fig 1:**
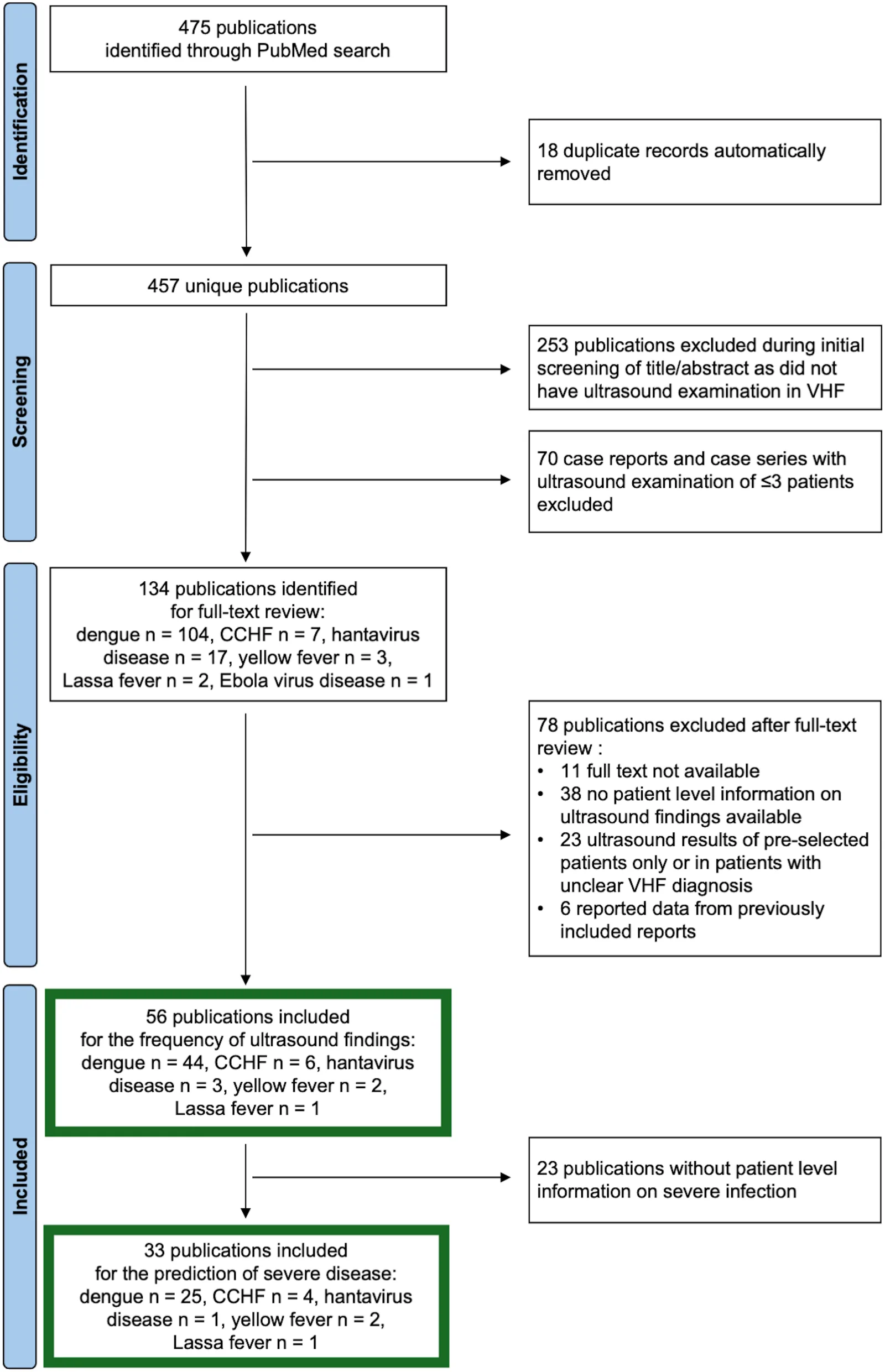
PRISMA flow diagram chronicling study selection, inclusion, and exclusion. CCHF: Crimean-Congo hemorrhagic fever.

The PubMed search yielded 457 unique publications for consideration. After screening the title/abstract for studies that presented original, patient-level data in confirmed cases of VaHFs, 134 publications remained for full text review. In total, 56 publications comprising 6979 participants were included in the systematic review, including 44 publications (6189 participants) on dengue, [48–91] six (494 participants) on CCHF, [10,92–96] three (134 participants) on hantavirus disease, [97–99] two (116 participants) on yellow fever, [100,101] and one (46 participants) on Lassa fever [35] (Fig 1). Several studies meeting other inclusion criteria were excluded for the absence of patient level ultrasound data [14, 102–134]. Included studies were published from 1991 – 2023 and recruited participants across the age spectrum and from 16 different countries across four continents (S4 Text). Patients were most often prospectively recruited (75.0%, 42/56 publications) from the inpatient setting (83.9%, 47/56 publications), although only a few studies explicitly mentioned that consecutive patients were recruited (S4 Text). The person performing the ultrasound was not reported in all studies, but when reported, this person was most often an expert sonographer (76.5%, 26/34 publications reporting this information), including radiologists, trained sonographers, or cardiologists (in the case of echocardiography-based studies), while fewer studies used a general physician or non-physician who had received a brief training in ultrasound (S4 Text). Among the included publications, only 59.8% (33/56 publications) provided information on disease severity, including 25 on dengue, four on CCHF, one on hantavirus disease, two on yellow fever, and one on Lassa fever (S4 Text). A complete list of all 457 publications that were screened, as well as the reason for exclusion if relevant, are provided in S5 Text.

### Risk of bias in the included studies

The main source of bias in patient recruitment occurred at the study level: participants were overwhelmingly recruited while acutely sick in the hospital setting during a period of high transmission at referral or tertiary care centers more likely to see more severe disease (S4 Text). No study recruited a community sample. Regarding the QUADAS-2, while the vast majority of studies prospectively recruited patients, most did not specify if recruitment was consecutive or not, leading to unclear amount of bias in patient recruitment (S4 Text). Only rarely did a study fail to either confirm all infections or complete ultrasound scans for all participants (S4 Text). The main source of bias captured by the QUADAS-2 tool occurred in studies on dengue in the timing domain as there was often a lack of distinction between the ultrasound finding (e.g., effusions) as related to the criteria for severe dengue (e.g., plasma leakage including effusions) and/or the timing of the ultrasound examination in relation to the classification of disease severity (S4 Text).

### Overall results

Across all VaHFs, ultrasound findings were common and included GBWT, ascites, pleural effusions and abnormal lung ultrasound, pericardial effusions, abdominal lymphadenopathy, splenomegaly, abnormal pancreatic ultrasound, and hyperechoic kidneys or liver (Table 1). Several findings had significant specificity and/or positive LR for severe disease (Table 2, Table 3).

**Table 1:** Frequency of ultrasound findings in viral infections associated with hemorrhagic fevers. For consistency, crude proportions are presented as percents for all findings. Weighted meta-analysis using a random-effects model was performed when there were at least three studies for any given finding, which was only possible for certain findings in dengue and Crimean-Congo hemorrhagic fever. CP: crude proportion. WMA: weighted meta-analysis. CI: confidence interval. GBWT: gall bladder wall thickening. *Crude proportion of findings among pediatric/adolescent and adult/adolescent patients with dengue compared using two-sided two-sample z-test.

	Dengue (pediatric/adolescent)		Dengue (adult/adolescent)			Dengue (all)		Crimean-Congo hemorrhagic fever		Hantavirus disease		Lassa fever		Yellow fever	
Finding	CP (95% CI) n WMA (95% CI)	No of studies	CP (95% CI) n WMA (95% CI)	No of studies	*p	CP (95% CI) n WMA (95% CI)	No of studies	CP (95% CI) n WMA (95% CI)	No of studies	CP (95% CI) n WMA (95% CI)	No of studies	CP (95% CI) n WMA (95% CI)	No of studies	CP (95% CI) n WMA (95% CI)	No of studies
B-lines	–	0	42 (28.3 – 55.7) 21/50	1	–	42.0 (28.3 – 55.7) 21/50	1	–	0	–	0	5.9 (0.0 – 13.8) 2/34	1	–	0
Effusion: Abdominal (ascites)	41.6 (39.4 – 43.7) 829/1995	13	25.2 (23.4 – 27.1) 534/2118	14	<0.001	35.3 (34.0 – 36.6) 1807/5120 39 (31 - 47)	34	18.3 (14.6 – 21.9) 78/427 19 (3 – 34)	4	25.3 (16.3 – 34.2) 23/91	2	31.8 (18.1 – 45.6) 14/44	1	30.4 (17.1 – 43.7) 14/46	1
Effusion: Pericardial	9.3 (7.7 – 11.0) 112/1203	8	2.0 (0.9 – 3.0) 14/712	6	<0.001	5.9 (4.9 – 6.9) 131/2228 6 (2 – 10)	16	38.8 (27.1 – 50.5) 26/67	2	4.5 (0.0 – 9.6) 3/66	2	21.7 (9.8 – 33.7) 10/46	1	14.3 (6.1 – 22.5) 10/70	1
Effusion: Pleural	41.7 (39.5 – 43.9) 832/1995	13	19.3 (17.7 – 21.0) 419/2168	15	<0.001	30.4 (29.2 – 31.7) 1573/5170 35 (27 – 44)	35	10.0 (5.5 – 14.5) 17/169	2	34.1 (24.3 – 43.8) 31/91	2	14.7 (2.8 – 26.6) 5/34	1	–	0
GBWT	52.5 (49.7 – 55.2) 685/1306	12	40.3 (38.0 – 42.7) 676/1676	14	<0.001	44.1 (42.5 – 45.6) 1758/3989 51 (42 – 60)	33	23.9 (19.8 – 27.9) 102/427 22 (5 – 38)	4	42.6 (30.9 – 54.4) 29/68	1	–	0	80.4 (69.0 – 91.9) 37/46	1
Hyperechoic kidneys	–	0	–	–	0	–	0	11.8 (7.0 – 16.7) 20/169	2	56.5 (36.3 – 76.8) 13/23	1	17.4 (6.4 – 28.3) 8/46	1	71.7 (58.7 – 84.8) 33/46	1
Hyperechoic liver	–	0	–	–	0	–	0	23.6 (16.7 – 30.5) 34/144	1	–	0	–	0	65.2 (51.5 – 79.0) 30/46	1
Lymphadenopathy: Mesenteric	4.7 (2.1 – 7.3) 12/254	1	–	0	–	4.7 (2.1 – 7.3) 12/254	1	8.5 (4.9 – 12.1) 20/235	2	–	0	–	0	–	0
Pancreas pathology	13.3 (10.3 – 16.4) 63/472	2	12.9 (10.0 – 15.8) 67/520	1	0.852	13.1 (11.0 – 5.2) 130/992 15 (4 – 27)	3	–	0	–	0	–	0	8.7 (0.6 – 16.8) 4/46	1
Splenomegaly	24.0 (21.3 – 26.6) 24/1009	7	9.8 (7.7 – 12.0) 71/722	6	<0.001	19.8 (18.2 – 21.4) 475/2399 23 (17 – 29)	17	19.9 (16.1 – 23.7) 85/427 20 (16 – 23)	4	36.8 (25.3 – 48.2) 25/68	1	–	0	–	0

**Table 2:** Test characteristics of ultrasound findings for predicting severity in dengue using only studies without significant concern for bias. Crude data used for these tables. See Supporting Information 4 for these included studies. CI: confidence interval. LR: likelihood ratio. GBWT: gall bladder wall thickening. *Likelihood ratio corrected using the treatment arm continuity correction.

A. All ages. The cutoff for GBWT was defined differently in each study, including >3mm, > 2mm, or even not defined in the one study. Splenomegaly in the one study was defined as >12 cm. There were no studies with results for B-lines, hyperechoic kidneys or liver, mesenteric lymphadenopathy, or pancreas pathology.						
Finding	Sensitivity 95% CI	Specificity 95% CI	Positive LR 95% CI	Negative LR 95% CI	Number of Patients	Number of studies
Effusion: Abdominal (ascites)	0.29 0.21 –0.39	0.80 0.77 –0.84	1.49 1.07 –2.09	0.88 0.78 –1.00	684	3
Effusion Pericardial	0.10 0.05 – 0.17	**1.00** **0.97 – 1.00**	*333.35 **323.86 – 342.83**	0.90 0.85 – 0.96	257	2
Effusion: Pleural	0.23 0.15 – 0.32	**0.96** **0.94 – 0.97**	**5.67** **3.34 –9.62**	0.80 0.73 – 0.89	684	3
GBWT	0.51 0.44 – 0.59	0.76 0.71 – 0.80	2.10 1.66 – 2.67	0.64 0.55 – 0.76	502	3
Splenomegaly	0.10 0.04 – 0.20	**0.99** **0.95 – 1.00**	**10.60** **1.33 – 84.30**	0.91 0.84 – 0.98	176	1
**B. Pediatric/adolescent only. The cutoff for GBWT was defined in the one study as >2mm. There were no studies with results for B-lines, pericardial effusions hyperechoic kidneys or liver, mesenteric lymphadenopathy, pancreas pathology, or splenomegaly.**						
Finding	Sensitivity 95% CI	Specificity 95% CI	Positive LR 95% CI	Negative LR 95% CI	Number of Patients	Number of studies
Effusion: Abdominal (ascites)	0.14 0.03 – 0.36	**0.93** **0.84 – 0.98**	2.18 0.53 – 8.95	0.92 0.76 –1.11	82	1
Effusion: Pleural	0.10 0.01 – 0.30	**0.98** **0.91 – 1.00**	**5.81** **0.55 – 60.84**	0.92 0.80 – 1.06	82	1
GBWT	0.05 0.00 – 0.24	**0.95** **0.86 – 1.00**	0.97 0.11 – 8.81	1.00 0.90 – 1.12	82	1
**C. Adult/adolescent only. In the one study investigating GBWT and splenomegaly, the cutoff for GBWT was not defined, while the cutoff for splenomegaly was 12 cm. There were no studies with results for B-lines, hyperechoic kidneys or liver, mesenteric lymphadenopathy, or pancreas pathology.**						
Finding	Sensitivity 95% CI	Specificity 95% CI	Positive LR 95% CI	Negative LR 95% CI	Number of Patients	Number of studies
Effusion: Abdominal (ascites)	0.33 0.23 – 0.43	0.79 0.75 – 0.82	1.55 1.10 – 2.18	0.85 0.73 – 0.99	602	2
Effusion Pericardial	0.10 0.05 – 0.17	**1.00** **0.97 – 1.00**	*333.35 **323.86 – 342.83**	0.90 0.85 – 0.96	257	2
Effusion: Pleural	0.19 0.10 – 0.30	**0.97** **0.92 – 0.99**	**6.56** **1.94 – 22.19**	0.84 0.75 – 0.94	176	1
GBWT	0.41 0.30 – 0.54	**0.95** **0.89 – 0.98**	**8.78** **3.57 – 21.60**	0.61 0.50 – 0.75	176	1
Splenomegaly	0.10 0.04 – 0.20	**0.99** **0.95 – 1.00**	**10.60** **1.33 – 84.30**	0.91 0.84 – 0.98	176	1

**Table 3:** Test characteristics of ultrasound findings for predicting severity in other VaHFs. Crude data used for these tables. CI: confidence interval. LR: likelihood ratio. GBWT: gall bladder wall thickening. *Likelihood ratio corrected using the treatment arm continuity correction.

A. Crimean-Congo hemorrhagic fever. Cutoff for GBWT was not specified in any of the three studies. The cutoff for splenomegaly was >12cm in one study and not specified in the other two studies. There were no studies with results for hyperechoic liver.						
Finding	Sensitivity 95% CI	Specificity 95% CI	Positive LR 95% CI	Negative LR 95% CI	Number of Patients	Number of studies
Effusion: Abdominal (ascites)	0.40 0.24 – 0.58	0.85 0.81 – 0.89	2.67 1.66 – 4.28	0.71 0.54 – 0.93	402	3
Effusion Pericardial	**1.00** **0.48 – 1.00**	0.64 0.47 – 0.79	2.78 1.83 – 4.24	*0.15 **0.00 – 2.63**	44	1
*Effusion: Pleural	0.00 0.00 – 0.41	**0.92** **0.86 – 0.96**	*0.12 0.00 – 7.20	1.09 1.03 – 1.14	144	1
GBWT	0.40 0.24 – 0.58	0.78 0.74 – 0.83	1.86 1.18 – 2.92	0.76 0.58 – 1.01	402	3
*Lymphadenopathy: Mesenteric	0.00 0.00 – 0.60	**0.96** **0.93 – 0.99**	*0.50 0.00 – 10.80	1.04 1.01 – 1.07	210	1
Splenomegaly	0.23 0.10 – 0.40	0.81 0.77 – 0.85	1.20 0.63 – 2.28	0.95 0.79 – 1.15	402	3
Hyperechoic kidneys	0.14 0.04 – 0.58	**0.93** **0.88 – 0.97**	2.18 0.32 – 14.85	0.92 0.68 – 1.25	144	1
**B. Hantavirus disease. GBWT defined as >4mm in the one study listed below. There were no studies with results for ascites, pleural effusions, B-lines, pericardial effusions hyperechoic kidneys or liver, mesenteric lymphadenopathy, pancreas pathology, or splenomegaly.**						
Finding	Sensitivity 95% CI	Specificity 95% CI	Positive LR 95% CI	Negative LR 95% CI	Number of Patients	Number of studies
GBWT	0.17 0.06 – 0.36	**1.00** **0.91 – 1.00**	*92.00 **84.74 – 99.26**	0.83 0.70 – 0.98	68	1
**C. Lassa fever. There were no studies with results for GBWT, hyperechoic liver, mesenteric lymphadenopathy, pancreas pathology, or splenomegaly.**						
Finding	Sensitivity 95% CI	Specificity 95% CI	Positive LR 95% CI	Negative LR 95% CI	Number of Patients	Number of studies
B-lines	0.00 0.00 – 0.60	**0.93** **0.78 – 0.99**	*0.18 0.00 – 8.32	1.07 0.97 – 1.18	34	1
Effusion: Abdominal (ascites)	0.14 0.36 – 0.58	0.64 0.47 – 0.80	0.41 0.06 – 2.63	1.32 0.90 – 1.94	44	1
Effusion Pericardial	0.14 0.36 – 0.58	0.77 0.61 – 0.89	0.62 0.09 – 4.15	1.11 0.79 – 1.58	46	1
Effusion: Pleural	0.6 0.15 – 0.95	**0.93** **0.77 – 0.99**	**8.70** **1.91 – 39.65**	0.43 0.15 – 1.26	34	1
Hyperechoic kidneys	0.57 0.18 – 0.90	**0.90** **0.76 – 0.97**	**5.57** **1.80 – 17.22**	0.48 0.20 – 1.13	46	1
**D. Yellow fever. GBWT defined as >3mm in the one study listed below. There were no studies with results for B-lines, hyperechoic kidneys or liver, mesenteric lymphadenopathy, pancreas pathology, or splenomegaly.**						
Finding	Sensitivity 95% CI	Specificity 95% CI	Positive LR 95% CI	Negative LR 95% CI	Number of Patients	Number of studies
Effusion: Abdominal (ascites)	0.34 0.17 – 0.56	0.75 0.51 – 0.91	1.36 0.55 – 3.49	0.87 0.60 – 1.27	46	1
Effusion Pericardial	0.19 0.09 – 0.33	**0.95** **0.77 – 1.00**	**4.13** **0.56 – 30.59**	0.85 0.72 – 1.00	70	1
GBWT	0.85 0.67 – 0.96	0.25 0.09 – 0.49	1.14 0.85 – 1.53	0.57 0.18 – 1.87	46	1

### Ultrasound in dengue

Among the 44 publications describing ultrasound findings in dengue, an outcome related to severity was included in 25 publications (S4 Text). Overall, risk of bias in patient selection, ultrasound testing, and the reference test was considered low or unclear in almost all studies (S4 Text). Participants were from Brazil, Cambodia, India, Indonesia, Malaysia, Mexico, Pakistan, Sri Lanka, Taiwan, Thailand, and Vietnam. One study reported on travelers to multiple of the above-listed countries. Seventeen of the studies included children/adolescents, 20 studies included adults/adolescents, five included participants of all ages, and the age range was not specified in two studies (though these studies included adults and possibly adolescents).

**Fig 2:**
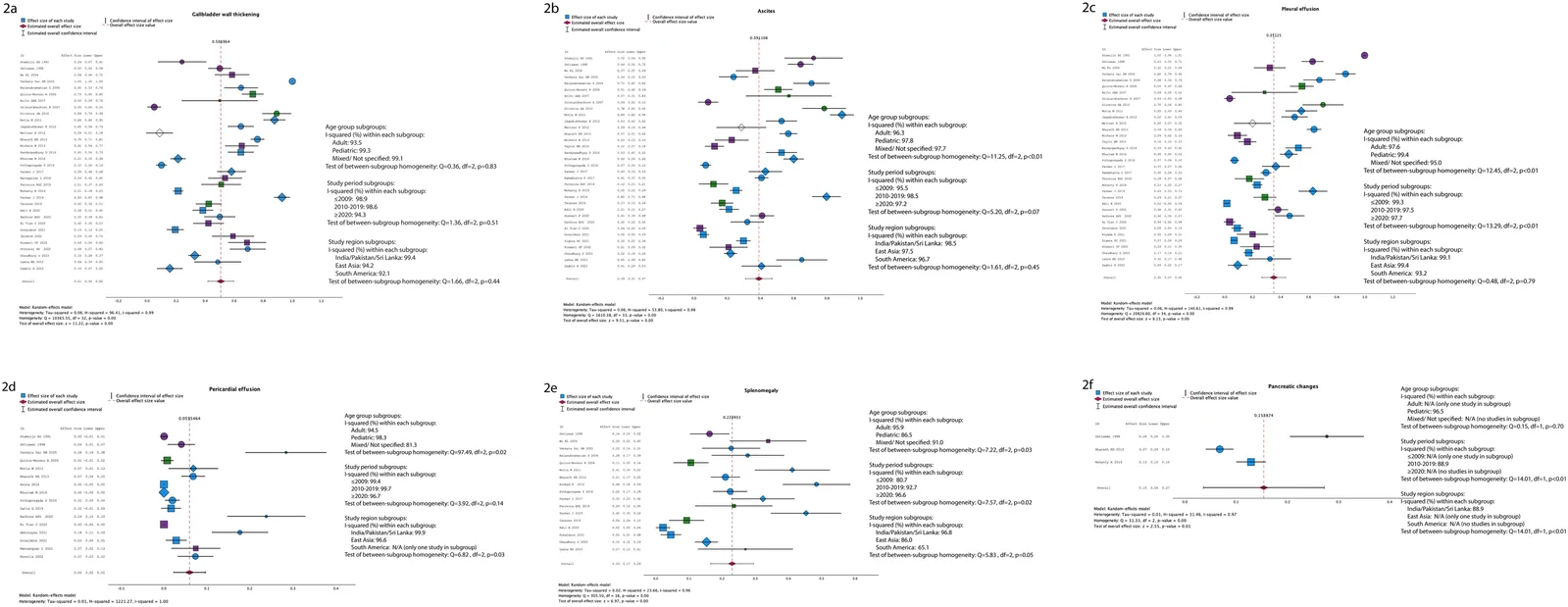
Forest plots showing the frequency of ultrasound findings in dengue. Random effects model employed. Publication year is listed with the first author under the study ID. Weight is represented by the size of the shape. Circles indicate studies with pediatric + /- adolescent participants, squares indicate adult + /- adolescent participants, and diamonds indicate mixed or unspecified age group of participants. Blue indicates studies from India/Pakistan/Sri Lanka, purple indicates studies from elsewhere in Asia, green indicates studies from South America, and white indicates participants from multiple countries. 2a. Gallbladder wall thickening. 2b. Ascites. 2c. Pleural effusion. 2d. Pericardial effusion. 2e. Splenomegaly. 2f. Pancreatic changes.

The frequency of ultrasound findings in dengue is shown in Table 1 and Fig 2. Regarding the crude proportions, GBWT was seen in almost half (44.1%, 1758/3989 participants from 33 studies) of participants. Effusions in the abdomen (35.3%, 1807/5120 participants from 34 studies) and in the pleural space (30.4%, 1573/5170 participants from 35 studies) were also common. Splenomegaly was seen in 19.8% (475/2399 participants from 17 studies). Effusions in the pericardial space (5.9%, 131/2228 participants from 16 studies) and other findings were less common.

Weighted meta-analysis was possible for most sonographic findings in dengue studies, and the results were similar to the crude data estimates (Table 1)**.** There was extreme heterogeneity in the frequency of ultrasound findings in dengue, and some of this heterogeneity resulted from differences in the age of participants, the era during which the study was conducted, and region (Fig 2). Test of between-subgroup homogeneity showed significant differences for subgroups of age for ascites, pleural effusion, pericardial effusion and splenomegaly; for subgroups of study region for pericardial effusion and pancreas changes; and for subgroups of study period for pleural effusion, splenomegaly and pancreas changes. Individual I^2^ as well as Q values for between-subgroup homogeneity are given in Fig 2.

Given the relatively consistent association of participant age on the frequency of ultrasound findings and the potential for important physiologic differences between children and adults, the crude proportions of ultrasound findings were examined separately for studies looking at pediatric/adolescent and adult/adolescent patients. Five studies included participants of all ages, and two studies did not specify the age range of participants, so these seven studies were excluded for this categorization (S4 Text). Seventeen studies included only pediatric/adolescent patients with an age range of 1 month to 18 years old, while 20 studies included only adult/adolescent patients with an age range of years 12 – 84 years old (S4 Text). Ascites, pericardial effusion, pleural effusions, GBWT, and splenomegaly were statistically more common among pediatric/adolescent patients compared to adult/adolescent patients (Table 1). Pleural effusions, for example, were found in 41.7% (832/1995 participants from 13 studies) of pediatric/adolescent patients compared to 19.3% (419/2168 participants from 15 studies) of adult/adolescent patients (z = 15.8, p < 0.001).

Among the 25 studies which included information on severity, there was significant concern for bias in the timing domain in 20 (80.0%) of these studies (S4 Text). This was related mainly to the definition of severity and/or the timing of the ultrasound examination in relation to the classification of disease severity. All five studies without bias were from the WHO Southeast Asia region including two from Thailand, two from Sri Lanka, and one from India (S4 Text). Three studies included only adults/adolescents, one included only children/adolescents, and one included patients of all ages (S4 Text). From these five studies, several ultrasound findings were significantly associated with severe disease (Table 2a). Regarding the crude data, pleural effusions had a specificity of 0.96 (95% CI 0.94 – 0.97) and a positive LR of 5.67 (3.34 – 9.62) for severe disease. Splenomegaly had a specificity of 0.99 (0.95 – 1.00) and a positive LR of 10.60 (1.33 – 84.30). All the participants with pericardial effusion had severe disease, therefore the specificity was 1.00 (0.97 – 1.00). No ultrasound finding had a clinically meaningful sensitivity or negative LR. As included studies used different cutoffs for gallbladder wall thickening, we attempted to investigate test characteristics of different cutoffs, but the data did not allow for such a comparison.

**Fig 3:**
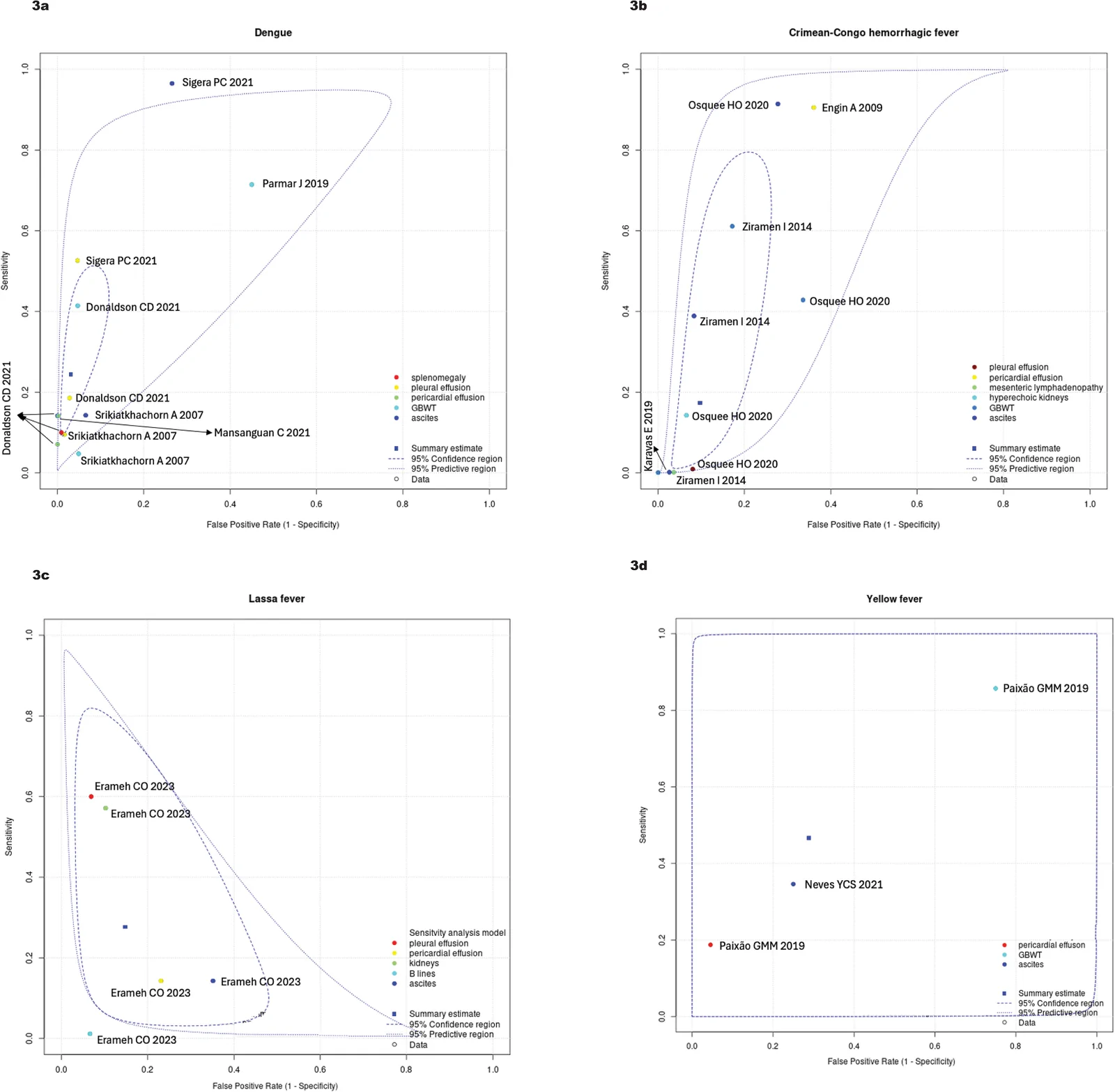
SROC plots of ultrasound findings for severe disease. The different ultrasound findings are identified by the different colors defined in each figure. Only a single study testing a single ultrasound finding was included for hantaviruses, so no SROC plot was created. 3a. Dengue. Results were available for splenomegaly (red), pleural effusion (yellow), pericardial effusion (green), gallbladder wall thickening abbreviated as GBWT (teal), ascites (dark blue). 3b. Crimean-Congo hemorrhagic fever. Results were available for pleural effusion (dark red), pericardial effusion (yellow), mesenteric lymphadenopathy (green), hyperechoic kidneys (teal), gallbladder wall thickening abbreviated as GBWT (light blue), ascites (dark blue). 3c. Lassa fever. Results were available for pleural effusion (red), pericardial effusion (yellow), hyperechoic kidneys (green), pulmonary B-lines (teal), ascites (dark blue). 3d. Yellow fever. Results were available for pericardial effusion (red), gallbladder wall thickening abbreviated as GBWT (teal), and ascites (dark blue).

Regarding the random effects meta-analysis of all findings for severe disease, the SROC plots (Fig 3a) demonstrate the low sensitivity and high specificity of most ultrasound findings as well as the heterogeneity of test characteristics with the large difference between the 95% confidence and 95% predictive regions. The sensitivity and specificity with weights are provided in S6a Text.

Regarding the prediction of severe disease among pediatric/adolescent patients and adult/adolescent patients (Table 2b and 2c), there was only one study without bias among pediatric/adolescent patients and two studies without bias for adult/adolescent patients (S4 Text), so these are described using crude data and in a narrative manner. The one study among children/adolescents only assessed for ascites, pleural effusions, and GBWT. All three findings were associated with severe disease, most notably pleural effusions showed a positive LR of 5.81 (0.55 – 60.84) and a specificity of 0.98 (0.91 – 1.00) (Table 2b). Among adults/adolescents, data from two studies found pericardial effusions were strongly associated with severe disease with a corrected positive LR > 300 (324 – 343) and specificity 1.00 (0.97 – 1.00), while data from one study found pleural effusions (positive LR 6.56 [1.94 – 22.19], specificity 0.97 [0.92 – 0.99]), GBWT (positive LR 8.78 [3.57 – 21.60], specificity 0.95 [0.89 – 0.98]), and splenomegaly (positive LR 10.60 [1.33 – 84.30], specificity 0.99 [0.95 – 1.00]) were all strongly associated with severe disease (Table 2c).

### Ultrasound in Crimean-Congo hemorrhagic fever

Six of the included publications described ultrasound findings in patients with CCHF, and an outcome related to severity was included in four of these six (S4 Text). Three publications included adults, one study included adults/adolescents, one study included children/adolescents, and the age range was not specified in one study. Five studies were conducted in Turkey, while one was conducted in Iran. Risk of bias was overall low, but three studies did not specify if recruitment occurred consecutively (S4 Text).

**Fig 4:**
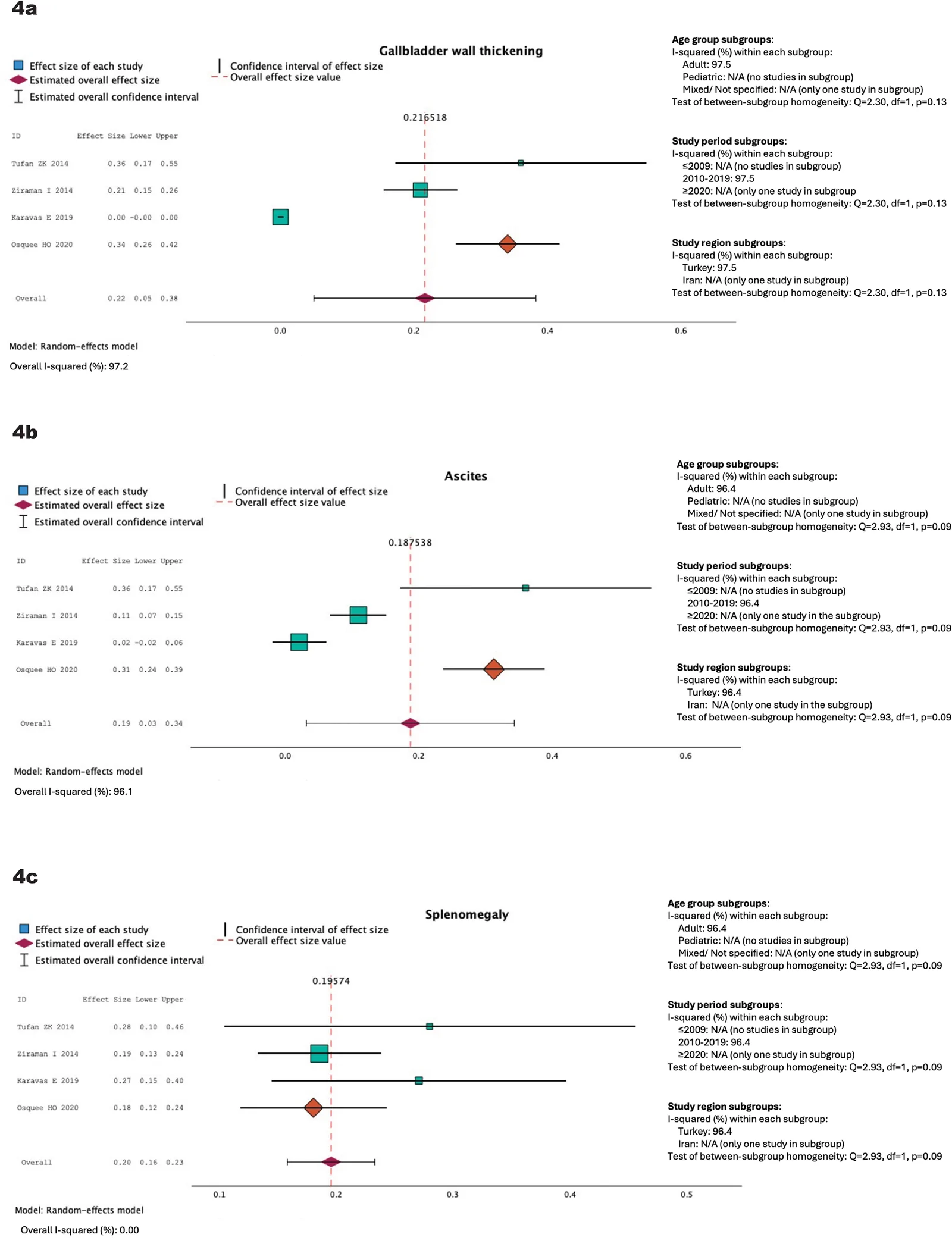
Forest plots showing the frequency of ultrasound findings in Crimean-Congo hemorrhagic fever. Random-effects model employed. Publication year is listed with the first author under the study ID. Weight is represented by the size of the shape. Circles indicate studies with pediatric + /- adolescent participants, squares indicate adult + /- adolescent participants, and diamonds indicate mixed or unspecified age group of participants. The teal color indicates studies from Turkey, while orange indicates the study from Iran. 4a. Gallbladder wall thickening. 4b. Ascites. 4c. Splenomegaly.

The frequency of ultrasound findings in CCHF is shown in Table 1 and Fig 4. Regarding the crude proportions, pericardial effusions were detected in 38.8% (26/67 participants from 2 studies) of participants. GBWT, splenomegaly, and ascites occurred in around 20% of the participants, whereas other findings were less common. Pleural effusions were found in only 10.0% (17/169 participants from 2 studies). One study also reported on echogenicity of the liver and renal cortex; liver echogenicity was increased in almost a quarter (23.8%, 34/143 participants) and the renal cortex was increased in 11.8% (20/169 participants).

Regarding the weighted meta-analysis, only three findings (GBWT, ascites, and splenomegaly) were reported in at least three studies (Fig 4). For all three findings, the four studies were the same, and included three studies among adults from 2010 – 2019 in Turkey and one study from 2020 in Iran with an unspecified age range. None of the tests of between-subgroup homogeneity for subgroups age, region and period were significant. Individual I^2^ as well as Q values for between-subgroup homogeneity are given in Fig 4.

The four studies with information on severity used death or various classifications of severity as the outcomes of interest (S4 Text). From the crude data available, pleural effusions (0.92, 95% CI 0.86–0.96), mesenteric lymphadenopathy (0.96, 0.93 – 0.99), and hyperechoic kidneys (0.93, 0.88 – 0.97) had high specificity for severe disease (Table 3a). Surprisingly, pericardial effusion had high sensitivity (1.00, 0.48 – 1.00) and significant negative LR (0.15, 0.00 – 2.63) for severe disease. Regarding the random effects meta-analysis of all findings for severe disease, the SROC plot (Fig 3b) demonstrates variability in both the sensitivity and specificity of ultrasound findings across studies, but again as expected, the findings are overall more specific than sensitive. The sensitivity and specificity with weights are provided in S6b Text.

### Ultrasound in hantavirus disease

Three publications describing ultrasound findings in patients with hantavirus disease were included, and only one provided information on disease severity (S4 Text). There was no ultrasound findings reported in at least three publications, so the results are presented only as crude proportions and in a narrative manner. A relatively high proportion of studies (29.4%, 5/17 studies) about hantavirus were inaccessible for full text review. Of the three included studies, two studies reported on adult/adolescent patients from Finland, and one study reported on adults/adolescents from Korea. In one study, there was high risk of bias in patient selection as only 61% of participants underwent ultrasound examination; risk of bias was otherwise low (S4 Text).

The frequency of ultrasound findings in hantavirus disease is shown in Table 1. Pleural effusions were found in about a third of participants (34.1%, 31/91 participants from 2 studies), and ascites in about a quarter (25.3%, 23/91 participants from 2 studies). GBWT (42.6%, 29/68 participants) and splenomegaly (36.8%, 25/68 participants) were only reported in a single study. One study described kidney findings, and hyperechoic kidneys were found in over half of participants (56.5%, 13/23 participants). In addition, the study also described parenchymal swelling in 73.9% (17/23 participants), disturbance in corticomedullary differentiation in 69.6% (16/23 participants), and a “patchy pattern” of parenchymal renal echotexture in 34.8% (8/23 participants). Pericardial effusions were not common (4.5%, 3/66 participants from 2 studies).

Only a single study testing a single ultrasound finding could be used to examine the test characteristics of ultrasound findings to predict severe hantavirus infection (S4 Text), so crude data are used and presented in a narrative manner. The study used death as the outcome of interest (Table 3b), and only GBWT (defined as >4 mm) was assessed. All five patients with the finding died yielding a specificity of 1.00 (95% CI 0.91 – 1.00) and an infinite uncorrected positive LR.

### Ultrasound in Lassa fever

Only one study describing ultrasound findings in patients with Lassa fever was included, and this study provided information about severity, which was defined as death (S4 Text). As there was only a single study, the frequencies are reported as crude proportions and in a narrative manner. The study was conducted in Nigeria and included adults/adolescents. Recruitment was not specified as consecutive or not, but otherwise, concern for bias was overall low (S4 Text).

The frequency of ultrasound findings in Lassa fever is shown in Table 1. The most common findings were ascites (31.8%, 14/44 participants), pericardial effusions (21.7%, 10/46 participants), hyperechoic kidneys (17.4%, 8/46 participants), and pleural effusions (14.7%, 5/34 participants). B-lines were uncommon (5.9%, 2/34 participants). Unfortunately, GBWT was not assessed.

From this single study (Table 3c), crude data show several findings had specificity ≥ 90% for severe disease, including pleural effusions (0.93, 95% CI 0.77 – 0.97), B-lines (0.93, 0.78 – 0.99), and hyperechoic kidneys (0.90, 0.76 – 0.97). Both pleural effusions (8.70, 1.91 – 39.65) and hyperechoic kidneys (5.57, 1.80 – 17.22) were found to have clinically meaningful positive likelihood ratios. The SROC plot visualizes the variability in both sensitivity and specificity (Fig 3c); the sensitivity and specificity with weights are provided in S6c Text.

### Ultrasound in yellow fever

Two publications describing ultrasound findings in patients with yellow fever were included, and both provided information on severity of infection (S4 Text). Both studies included adult participants from Brazil. One study was retrospective and did not specify if consecutive patient records were examined leading to unclear bias in patient selection (S4 Text).

As there were no ultrasound findings reported in at least three publications, the frequency of ultrasound findings is presented as crude proportions and in a narrative manner (Table 1). GBWT was only reported in one study, but was the most common finding (80.4%, 37/46 participants) reported in the entire review. Pericardial effusion was found in 14.3% (10/70 participants from one study) and ascites in 30.4% (14/46 participants from one study). One study reported on echogenicity of the kidneys and liver, which were frequently found to be hyperechoic (71.7%, 33/46 participants for kidneys; 65.2%, 30/46 participants for liver).

The reference for severity in one study was a national Brazilian severity grading scale, while the other study used death as the outcome of interest. From the crude data, pericardial effusion was significantly associated with severe disease (Table 3d) with a high specificity (0.95, 95% CI 0.77 – 1.00) and positive LR (4.13, 0.56 – 30.59). The SROC plot also demonstrates that GBWT showed a relatively high sensitivity relative to other findings in the review (Fig 3d); the sensitivity and specificity with weights are provided in S6d Text.

## Discussion

In this systematic review, we evaluated studies using ultrasound in viral infections associated with hemorrhagic fevers (VaHFs) to assess the frequency of ultrasound findings and the test characteristics of ultrasound findings to predict disease severity. Patients were most often recruited while acutely ill in the inpatient setting at referral hospitals, but there was wide variability in the geography of the studies and age of participants. However, key fundamental lessons and commonalities across the different VaHFs can be derived. Unfortunately, the review was dominated by studies on dengue, while evidence for other VaHFs was quite limited or absent. Therefore, our impressions and conclusions relate most strongly to dengue, and additional studies are needed to further validate these findings in non-dengue VaHFs.

An important study-level commonality among the included publications was that patients were recruited in an emergency or inpatient setting, often at tertiary referral centers during periods of high-transmission or outright epidemics and therefore were more likely to have more severe disease. Thus, the findings of this review apply most directly to patients presenting to acute care hospitals during periods of high transmission. We specifically avoided metrics such as positive or negative predictive values that require information on population prevalence. Our review cannot comment on patients with mild disease presenting to primary care clinics or asymptomatic community members found to be positive for viruses associated with hemorrhagic fevers.

The frequency of ultrasound findings across the included studies showed significant heterogeneity, which can be explained by several factors including different ages of patients, different countries in which the studies were performed, different cutoff values for findings that require a cutoff, different inclusion criteria among the included studies, and different eras during which the included studies were conducted. Only the studies on dengue allowed for formal analysis of heterogeneity of the frequency of ultrasound findings, and age was consistently found to be partially responsible for the heterogeneity of the findings with ultrasound findings reported more commonly among pediatric patients. The other subgroups of study period and region were only sometimes significant; for example, earlier studies were more likely to report pleural effusions. However, these three subgroups did not explain all the heterogeneity.

At the patient level, there were many commonalities in the frequency of sonographic findings across VaHFs. These patients are prone to extravasation of intravascular fluid, the so-called “plasma leakage” characteristic of these infections that is driven by the inflammatory milieu. Such shifting of fluid from the intravascular to extravascular space occurs in many other conditions, such as failure of the heart, kidneys, or liver; sepsis and septic shock; anaphylaxis and anaphylactic shock; and the hyperinflammatory states of acute pancreatitis or Kawasaki disease. The sonographic findings described above help identify extravascular fluid and may therefore assist in identifying or predicting more severe disease states. GBWT and effusions of the abdomen and pleural space were common findings, while pericardial effusions were overall less common but more consistently predictive of severe disease. Some sonographic findings, such as effusions, were commonly assessed across VaHFs, whereas others, such as pulmonary B-lines or changes in kidney echogenicity, were more frequently evaluated in some VaHF but not others.

As noted above, the frequency of ultrasound findings was more common among children than adults. Other studies have also found higher incidence of plasma leakage among children compared to adults across several countries [135,136]. Risk of death may also vary by age, as younger age is associated with *lower* mortality in dengue, [137] CCHF, [93] Lassa fever, [138] and yellow fever [139]. Interestingly, age was not found to be associated with mortality in hantavirus pulmonary syndrome [140]. Children may have a larger and more permeable microvascular surface area so that endothelial dysfunction leads more readily to extravasation of plasma in children compared to adults [135]. Immune status may also affect the development of plasma leakage; however, the incidence of sonographic signs of plasma leakage was similar in primary and secondary cases among travelers returning with dengue infection, suggesting plasma leakage reflects the underlying pathophysiology of the disease and not the patient’s immune status [59].

### Ultrasound for early detection of severe disease and the prediction of future clinical deterioration

A key point raised from the dengue literature concerns the difference between ultrasound used for early detection of severe disease versus prediction of future outcomes. We agree with Sigera et al. who state plainly “since one of the criteria for severe dengue or DHF is plasma leakage, correlating sonographic detection of extravasated fluid with these adverse outcomes becomes a circular argument” [84]. When ultrasound findings help a patient meet criteria for more severe disease, then the findings cannot truly be said to *predict* severe disease as this constitutes incorporation bias. Even when ultrasound findings are not included in the severity definition, the timing of the ultrasound relative to the assessment of disease severity may introduce additional bias. In some studies, serial ultrasound examinations were performed, but the temporal relationship between the ultrasound findings and the classification of disease severity was not clearly reported; these studies were therefore judged at risk of bias in the timing domain and were not included in the analyses of the prediction of severe disease. Thus, it is critical to separate early detection of existing severe disease from the prediction of future clinical deterioration.

Ultrasound can allow for early detection of established severe disease by detecting plasma leakage, [52,55,61,79] and new severity definitions for VaHFs should integrate ultrasound findings with findings from the physical exam and laboratory. Incorporation of ultrasound findings into the definitions of severe disease also avoids the circular conundrum described above. For example, GBWT, effusions, and lung B-lines in the clinically stable patient could be listed with an increase in hematocrit under the definition of Dengue with Warning Signs. Applying the 2009 WHO classification for dengue, clinical fluid accumulation or increase in hematocrit are warning signs for severe dengue and severe plasma leakage automatically classifies the patient as Severe Dengue [41]. In the 2011 iteration, any plasma leakage automatically classifies the patient as Dengue Hemorrhagic Fever [3]. Other authors have suggested that a minimal amount of liquid can be considered as mild plasma leakage and a warning sign while moderate-severe fluid can be considered as severe plasma leakage and thus be diagnostic for Severe Dengue [77]. While not the focus of this review, assessment of intravascular status could also be incorporated.

**Fig 5:**
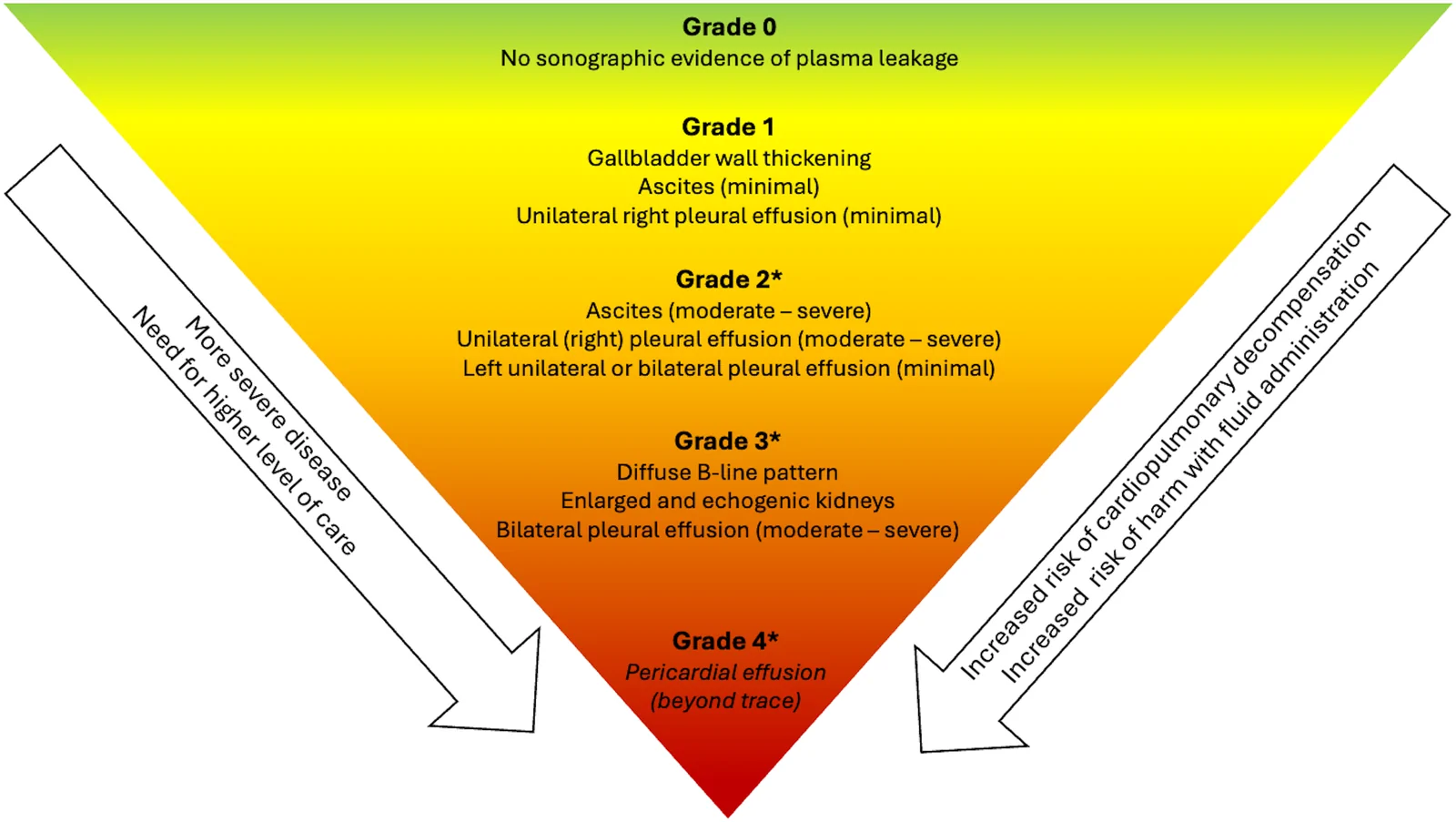
Ultrasound-based assessment of Plasma leakage Severity (UPS) for patients with viral infections associated with hemorrhagic fevers and with stable hemodynamics and respiratory status. *Each higher grade may include any findings from lower grades as well as any finding for that grade. For example, Grade 3 may include gallbladder wall thickening and ascites from lower grades along with any of the Grade 3 criteria: diffuse B-lines or enlarged and echogenic kidneys or bilateral pleural effusions. Source: This is an original image created by the authors; please cite this current manuscript.

Ultrasound findings may also help predict adverse outcomes among both children and adults. Based on the evidence described in our review and considering common pathways of inflammation, vascular leakage, and shock, we propose an Ultrasound-based assessment of Plasma leakage Severity (UPS) in VaHFs as a hypothesis generating framework for patients of any age to serve as a starting point for future, prospective studies (Fig 5). Lower grades include more common findings less associated with severe disease (such as GBWT or ascites), while higher grades include less common findings (such as pericardial or large pleural effusions) associated with possible respiratory or hemodynamic changes and more severe disease. The optimal cutoff value for GBWT needs to be investigated further, and there may be utility to different cutoff values in different clinical situations. While strict quantitative measurement of effusions is likely not necessary, semi-quantitative methods can help standardize definitions of “mild”, “moderate”, and “severe”. For ascites, rather than calculating the Ascites Index, [141] the vertical height of the fluid pocket with the patient in the supine position could be used [88]. For pleural effusions, rather than multi-section approaches such as the Remerand method, [142,143] single-measurement techniques such as the Balik method or simply measuring the distance between the tip of the lung and the diaphragm could be used [142,144]. For pericardial effusions, the maximal distance of the effusion between the heart and the pericardium at end-diastole can be used [145]. We propose the UPS as a hypothesis generating framework only, as future research among different age groups will need to help define semi-quantitative cutoff values and associate these grades with future clinical deterioration as discussed below. More research is clearly needed before such an ultrasound-based grading system will be ready for clinical implementation.

### Virus- and organ-specific considerations

The literature from each virus raises important considerations that may be applied across VaHFs, including the potential role of intravascular assessment, caution in interpreting third spacing in peritoneal organs as acute bacterial infection, the potential role for cardiac ultrasound, and the potential for under-utilized organ specific assessments such as pulmonary B-lines or renal echogenicity.

Dengue highlights the role of ultrasound to query the intravascular space. In the 2009 dengue guidelines, the WHO states explicitly that intravenous rehydration is the therapy of choice that can reduce the case fatality rate to less than 1% of severe cases [41]. At the same time, similar to seminal trials in pancreatitis, [146] studies on fluid resuscitation in VaHFs have demonstrated the possibility of harm with aggressive resuscitation with intravenous fluids. A review on the use of fluids during the critical phase of dengue characterized the goal of fluid resuscitation to be normalization of hemodynamics and urine output to avoid indiscriminate use of fluids and fluid overload; the review noted that fluids are usually only needed during the critical phase for 24–36 hours [147]. The authors propose specific instructions for fluid resuscitation in Dengue with Warning Signs and Severe Dengue Disease and conclude, “Limiting the duration of [intravenous fluid] therapy to the minimum necessary is essential to reduce the occurrence of intravascular fluid overload during the recovery phase” [147]. One study found that the risk of developing plasma leakage detected by ultrasound was more than tripled for patients who received intravenous fluids prior to admission compared to those who did not receive fluids prior to admission [63]. The Malaysia Health Technology Assessment Section 2020 Clinical Practice Guidelines for the Management of Dengue in Children states explicitly “the higher the fluid infusion rate, the more frequent the patient should be monitored and reviewed in order to avoid fluid overload…” and notes that ultrasound can be utilized as part of this monitoring [148]. While not the focus of this review, ultrasound can help guide fluid resuscitation by directly visualizing intravascular spaces [149]. Fluid resuscitation should be guided by several data points (such as clinical status, urine output, hematocrit, coagulation markers), and ultrasound can also be a useful tool to assess for signs of plasma leakage in extravascular spaces and to gauge the intravascular fluid status.

Dengue also highlighted confusion about GBWT and acute surgical cholecystitis [150]. Similar to the literature on dengue, there were also case reports of “acalculous cholecystitis” in hantavirus disease [151,152]. GBWT seems to be an early finding in dengue, peaking between day 5–8 of illness [75,105]. A recent meta-analysis looking specifically at GBWT in dengue also found a significant association with severe disease [39]. In the right clinical context, GBWT is a sign of acute surgical cholecystitis, and thus some authors label GBWT in VaHFs as “acalculous cholecystitis”. However, GBWT can be seen also in other inflammatory states with third-spacing, such as pancreatitis, [153] advanced chronic liver disease with clinically significant portal hypertension, [154] and acute viral hepatitis [155].

Among the many reports in literature of “acalculous cholecystitis” in patients with dengue, [107,156–171] cholecystectomy was performed in some cases [107,163,168,169,171]. In one series of 14 patients with striated GBWT > 3 mm and positive sonographic Murphy’s sign, all patients recovered without cholecystectomy [150]. In another series, when sonographic criteria for cholecystitis of GBWT > 3.5 mm and positive sonography Murphy’s sign were applied to patients with dengue fever, about 28% would have met criteria for acute cholecystitis; however, all 11 patients improved without surgical intervention [170]. In one series of 357 patients with dengue fever and concern for acute abdomen, three emergent cholecystectomies were performed for suspected perforated/gangrenous cholecystitis and 16 elective cholecystectomies for acalculous cholecystitis were planned for outpatients. Of the three completed cholecystectomies, none were found to be perforated. Of the 16 planned outpatient cholecystectomies, symptoms had resolved in all patients thereby obviating the need for surgery [107]. Only very rarely have pathological findings been consistent with true surgical cholecystitis [169]. Thus, GBWT in dengue reflects the inflammatory state and plasma leakage, rather than an acute surgical cholecystitis, and therefore usually resolves without sequalae [56]. Conservative management under continuous observation is advised to prevent unnecessary morbidity and mortality in patients with dengue fever and GBWT [164]. Immediate surgical intervention has been associated with increased need for blood products and longer hospital stay [107,171]. If bacterial superinfection is a concern, a trial of antibiotics may offer a more favorable risk-benefit ratio than surgery.

Similarly, several case studies discussed appendicitis and pancreatitis in dengue, [113,172–177] and again, patients sometimes endured surgery for concern for appendicitis [172,174,175]. In the abovementioned series of 357 patients, five patients underwent appendectomy for concern of perforated appendicitis; none had evidence of perforation in the operating room [107]. In a series of 12 patients in Sri Lanka with concern for appendicitis, all but one improved with conservative management. Pathology from the one patient who underwent surgery showed follicular lymphoid hyperplasia consistent with generalized inflammation [113]. Again, only very rarely have pathological findings been consistent with acute ulcerative appendicitis [174].

Hantavirus disease, specifically the manifestations of hantavirus pulmonary syndrome and hemorrhagic fever with renal syndrome, highlights the role for ultrasound to evaluate the lung and renal parenchyma. Only two included studies, one in dengue [82] and one in Lassa fever, [35] assessed for pulmonary B-lines, and the relationship with severe disease was not clear. Another study, which was not included in the systematic review as cases were not confirmed by serology, assessed for B-lines in dengue and found B-lines in fewer than 15% of children; the small number of true positives precluded useful association with severity [178]. Hyperechoic kidneys were associated with severe in disease in CCHF and Lassa fever, [35,96] while no included studies on dengue assessed for changes to the renal parenchyma.

Several VaHFs raise considerations of cardiac involvement. In addition to the cardiac tropism of yellow fever, cardiac tissues can also be directly affected in dengue [106] and hantavirus disease [179]. For hantavirus disease, some authors prefer the term “hantavirus cardiopulmonary syndrome” to emphasize cardiac features of the clinical presentation [180,181]. Myocarditis can complicate dengue, [71,106,111,115,118,123,182] CCHF, [183] hantavirus disease, [184,185] yellow fever, [100,186] and Ebola virus disease [187]. While fluid shifts play a prominent role in the hypotension and shock encountered in patients with VaHFs, other etiologies of shock, including hemorrhagic and cardiogenic causes, must also be considered. Authors from New Delhi noted wryly “The WHO guidelines on management of [dengue] assumes fluid deficit to be the only reason for hypotension” [188]. The above-cited set of authors noted the persistence of shock despite fluid resuscitation and high central venous pressure among children with dengue and so conducted an echocardiographic study which revealed a left ventricular ejection fraction <50% in 17% of participants [188]. While not the focus of this review, cardiac ultrasound could help identify cardiac dysfunction in patients whose abnormal hemodynamics persist after correction of fluid shifts and acute blood loss.

### Limitations

There were several important limitations to this review. First, the review used a single database (PubMed), so may have missed studies from journals that are not listed in the database. Specifically, we may have missed publications from local journals where VaHFs are more prevalent, for example from South America, Eastern Europe, or Africa. Second, the review was dominated by studies about dengue, while studies on other VaHFs were lacking. For several VaHFs, most notably Ebola, there were no studies that could be included in the review, and there were no studies on hantaviruses in the Americas. Third, although there may be important differences between pediatric and adult patients, studies could only be stratified by age group for dengue. Fourth, there was significant heterogeneity in the reported frequency of ultrasound findings which was not completely explained by the three explored subgroups of age, region, and study period. One consistency, however, was that the included studies mainly recruited from the inpatient setting at tertiary care centers in high-prevalence geographies during a known epidemic and therefore likely reflected more severe disease. Thus, the results likely do not apply to asymptomatic disease in the community or well-appearing patients presenting for lower acuity, outpatient care in geographies with low transmission. Fifth, analysis of the predictive accuracy of ultrasound findings for severe disease was limited to the much smaller proportion of included studies reporting on severity and satisfactorily free of bias; thus, the opportunity for formal meta-analysis was greatly limited. Sixth, specifically related to dengue, there were issues of bias or lack of clarity related to 1) the definition of severity and whether ultrasound findings impacted the category of severity and 2) the timing of the ultrasound assessment in relation to the determination of severity. Finally, ultrasound in most studies was performed by physician experts, rather than general physicians or other health-care workers with more limited ultrasound training.

### Considerations for future research

As there were several limitations to our current review, and we make hypothesis generating proposals for incorporating ultrasound into definitions of severity and a new ultrasound-based framework for grading severity, our work highlights the need for future, prospective research in multiple key areas.

First, ultrasound findings need to be clearly defined and standardized to allow comparison of their frequency and test characteristics across studies. Among adults, a GBWT cutoff of 3.5 – 4.0 mm is commonly accepted for acalculous acute cholecystitis and may be a starting point for future research in VaHFs [189]. Regarding splenomegaly, 12 cm is a commonly accepted cutoff, but height-and sex-corrected values may be more accurate [190]. In children, a GBWT cutoff of 3.0 mm is generally accepted across the pediatric age range for acute cholecystitis, [191] but some authors suggest a higher cutoff [105]. Assessment of splenomegaly in children requires comparison to normative values for age, height, and potentially sex [192–194]. Regarding plasma leakage, exact quantification of the volume of extravascular fluid can be difficult, and so qualitative or semi-quantitative assessment as discussed above may be more useful and replicable than attempts at exact quantification. Future research will need to investigate the utility of different cutoff values. Generally, a lower cutoff would be expected to be more sensitive, while a higher cutoff would be expected to be more specific; each might have a practical use depending on the clinical context and triage workflow.

Second, the timing of ultrasound findings needs to be investigated in relation to the disease course, rather than in relation to the hospital course, and there may be benefit to serial examinations. For example, GBWT can be a useful tool both early in the disease course to identify early plasma leakage and later during the recovery phase to identify improvement. Initially, GBWT should be assessed between day 3–7 of illness to correspond to the onset of vascular leak [75,105]. Among adults with confirmed, non-severe dengue without clinical or hematocrit evidence of plasma leakage, one study found that almost half (45.8%) had ultrasonographic evidence of plasma leakage, and among these, 92% developed Dengue with Warning Signs or Severe Dengue Disease [79]. During the recovery phase, resolution of GBWT reflects overall clinical improvement as patients may recover 2–3 days after GBWT resolves [79].

Third, prospective studies are needed to investigate the association between ultrasound findings, including ultrasound-based severity scales such as our UPS, and future clinical deterioration, such as need for supplemental oxygen, intubation and mechanical ventilation, vasopressors or inotropes for hemodynamic support, renal replacement therapy, blood products for coagulopathy and hemorrhage, readmission, or death. In addition to the common findings of GBWT and effusions, studies should also investigate the less commonly assessed findings, including B-lines in the lung parenchyma, changes in renal and liver echogenicity, mesenteric lymphadenopathy, and splenomegaly. As the frequency of findings and overall case fatality rate will affect the test characteristics of ultrasound findings for severe disease, future studies should 1) recruit independent cohorts of pediatric and adult patients and 2) recruit separate cohorts of sick inpatients, symptomatic outpatients, and asymptomatic community members.

Fourth, the role of ultrasound in guiding triage, disposition, and fluid resuscitation in VaHFs should be investigated further in prospective studies. Faster triage can help save lives: For example, during the 2018–2020 Ebola virus disease outbreak, a clinical trial of several treatments found that 97% of the deaths in the trial occurred within 10 days after enrollment, and each day with symptoms prior to enrollment was associated with an 11% increase in the odds of death, thereby emphasizing the importance of early detection, rapid triage, and expeditious identification of patients with more severe disease [28]. Ultrasound assessment can help determine level of care (standard ward vs unit with capacity for more intensive monitoring), the type of resuscitation (oral vs intravenous crystalloids vs intravenous colloids), or need for additional interventions (antiviral medications and/or steroids). Furthermore, while our review did not include fundamental ultrasound assessment of intravascular volume status (e.g., inferior vena cava, internal jugular vein, or basic cardiac views), ultrasound can help guide fluid resuscitation in patients who are fluid tolerant or fluid responsive and avoid harmful administration of fluids in patients at risk for fluid-related adverse events. As always, ultrasound findings cannot be interpreted in isolation and should always be taken within the context of the clinical exam, laboratory studies, and additional imaging results. Consideration for admission to a unit capable of close monitoring for critically ill patients is recommended for patients with any signs of clinical instability, and high-risk patients may need higher level of care even if they appear relatively stable. For example, obesity and diabetes have been associated with higher risk of severe dengue infection [195,196]. Independent of the patient’s clinical status, public health considerations must also be weighed, and so the risk of transmission to the community or at-risk community members may also affect appropriate disposition. Overall, we hypothesize that the use of ultrasound to guide triage and management can help reduce mortality in patients with VaHFs, and this should be tested in rigorous clinical trials.

Finally, given the millions of cases of VaHFs each year, many of them occurring in settings without adequate medical personnel, future research must demonstrate that front-line health care workers can be trained in simple ultrasound protocols to achieve the same outcomes as providers with decades of experience and/or advanced training in ultrasound [197]. An important characteristic of any successful protocol is its ability to change management, and furthermore, any ultrasound protocol must be easily implementable by front line health care workers where patients seek care, scalable under epidemic conditions, and teachable to front line health care workers with varying levels of training. In many setting where VaHFs occur, POCUS may be the only imaging modality available and can therefore have an even greater impact on patient outcomes than in high-resource settings in which advanced cross-sectional imaging may be obtained rapidly. By integrating ultrasound training into broader medical and allied health education, we can enhance diagnostic capabilities and improve decision-making, especially in resource-limited settings. A recent meta-analysis on task shifting found that POCUS protocols can be used by non-physicians, including midwives, nurses, clinical officers, technicians, and community health workers [197]. Logistical issues, such as electricity and internet connectivity, will need to be addressed [197]. The implementation of a POCUS protocol for Lassa fever in Nigeria demonstrated that it is possible to perform scans in isolation wards in an endemic area, and potential challenges were also highlighted [35]. For example, the gallbladder was deemed too complicated and not included in the protocol, thoracic views proved challenging, and the need to swap probes added extra time and hassle for sonographers [35]. Similarly, a study on patients with dengue from a primary hospital in Columbia assessed the quality of image acquisition and compared interpretation of POCUS images by a general physician and a radiologist [198]. A general physician without prior ultrasound experience completed a short training and acquired the images using a common portable ultrasound device, and the scans were then independently interpreted by both the general physician and a radiologist. Only about half of the acquired images were deemed by the radiologist to be of adequate quality to assess plasma leakage, and the level of agreement for the detection of plasma leakage between the general physician and the radiologist was only deemed fair (kappa 0.25) [198]. Such findings emphasize the importance of teachability and scalability. Finally, future research will need to investigate the role of remote and artificial intelligence guidance to aid acquisition and interpretation.

## Conclusions

Ultrasonography can be a useful tool for triage and management of viral infections associated with hemorrhagic fevers. Common pathophysiology leading to plasma leakage results in some frequent sonographic signs seen across VaHFs, while some VaHFs present with organ-specific findings also detectable on ultrasound. We recommend that ultrasound findings of plasma leakage be incorporated into new definitions of severity for VaHFs. Sonographic signs may also help predict future clinical deterioration, and we propose an ultrasound-based grading system for VaHFs. There is a need for prospective studies to better define ultrasound findings and their frequency, the appropriate time for ultrasound assessment in relation to the phase of illness, associations between ultrasound findings and specific clinical deterioration, the role of ultrasound in triage and management, and how ultrasound can best be implemented by front line clinicians.

## Supporting information

S1 Text PubMed search string. (DOCX)

S2 Text Adapted QUADAS-2 tool. (DOCX)

S3 Text Iterations of World Health Organization (WHO) classifications for the spectrum of disease severity in dengue. (DOCX)

S4 Text Publications included in the systematic review. (DOCX)

S5 Text All screened publications. (DOCX)

S6 Text Sensitivity and specificity with weights from the meta-analysis of ultrasound findings for severe disease. (DOCX)

S1 Data Datasets (Excel) used in the systematic review. (ZIP)

S1 File PRISMA checklist. (DOCX)
