# Supplementary material for: A systematic review of ultrasound to predict severe disease in patients with dengue and other viral infections associated with hemorrhagic fevers
Source: PLoS Negl Trop Dis. 2026 Sep 17;20(9):e0014609. doi: 10.1371/journal.pntd.0014609 (PMC13585234; doi:10.1371/journal.pntd.0014609)
Supplement: Supplementary file 1 [file pntd.0014609.s001.docx]

Supporting Information 1: PubMed search string.

("ultrasound" OR "ultrasonography" OR "sonography" OR "echography" OR "echocardiography" OR “echo*”)

AND

("viral hemorrhagic fever" OR “viral haemorrhagic fever" OR "dengue" or "lassa" OR "crimean” OR “crimean-congo" OR "ebola" OR "Marburg virus" OR "yellow fever" OR "hanta" OR "puumala" OR "HFRS" OR "hemorrhagic fever with renal syndrome" OR “haemorrhagic fever with renal syndrome” OR "rift valley" OR "junin" OR "machupo" OR "Sabia" OR "guanarito" OR "bunyavirus" OR "filovirus" OR "flavivirus" OR "arenavirus")
