# Supplementary material for: A systematic review of ultrasound to predict severe disease in patients with dengue and other viral infections associated with hemorrhagic fevers
Source: PLoS Negl Trop Dis. 2026 Sep 17;20(9):e0014609. doi: 10.1371/journal.pntd.0014609 (PMC13585234; doi:10.1371/journal.pntd.0014609)
Supplement: Supplementary file 2 [file pntd.0014609.s002.docx]

Review Question: Can ultrasound findings predict severe disease in patients with viral infections associated with hemorrhagic fever?

Index test: Ultrasound

Reference standard: Severe disease as determined by each individual study

Domain 1: Patient selection

A. Risk of Bias
   - Was a consecutive or random sample of patients enrolled? (Yes/No/Unclear)
   - Did the study avoid inappropriate exclusions? (Yes/No/Unclear)
   - *Could the selection of patients have introduced bias? (Low/High/Unclear)*
B. Concerns regarding applicability

- *Is there concern that the patients do not match the review question? (Low/High/Unclear)*

Domain 2: Index test (ultrasound)

A. Risk of Bias
   - Were ultrasound results interpreted without knowledge of the presence/absence of severe disease? (Yes/No/Unclear)
   - *Could the conduct or interpretation of the ultrasound examination have introduced bias? (Low/High/Unclear)*
B. Concerns regarding applicability

- *Is there concern that the ultrasound examination, its conduct, or interpretation differ from the review question? (Low/High/Unclear)*

Domain 3: Reference standard (severe disease)

A. Risk of Bias
   - *Is the definition of severe disease likely to correctly classify the target condition? (Yes/No/Unclear)*
   - *Could the definition of severe disease or its interpretation have introduced bias? (Low/High/Unclear)*
B. Concerns regarding applicability

- *Is there concern that the definition of severe disease does not match the review question? (Low/High/Unclear)*

Domain 4: Flow and timing

Risk of Bias

- *Did all patients have an assessment of disease severity? (Yes/No/Unclear)*
- *Did all patients have the same assessment? (Yes/No/Unclear)*
- *Were all patients included in the analysis? (Yes/No/Unclear)*
- *Was the ultrasound done BEFORE the level of severity was determined? (Yes/No/Unclear)*
- *Could the patient flow (i.e. the timing of the ultrasound examination) have introduced bias? (Low/High/Unclear)*

**References**

1.	Whiting PF, Rutjes AWS, Westwood ME, *et al.* QUADAS-2: a revised tool for the quality assessment of diagnostic accuracy studies. *Ann Intern Med* 2011; **155**: 529–36.
