# Supplementary material for: A systematic review of ultrasound to predict severe disease in patients with dengue and other viral infections associated with hemorrhagic fevers
Source: PLoS Negl Trop Dis. 2026 Sep 17;20(9):e0014609. doi: 10.1371/journal.pntd.0014609 (PMC13585234; doi:10.1371/journal.pntd.0014609)
Supplement: Supplementary file 3 [file pntd.0014609.s003.docx]

|  | WHO 1997[1] | WHO 2009[2] | WHO-SEA 2011[3] |
| --- | --- | --- | --- |
| *Mild Disease* | Dengue Fever Syndrome:  Probable symptoms + supportive serology/PCR | Dengue without Warning Signs:  Probably symptoms + lab confirmation | Fever with two of following: headache, retro-orbital pain, myalgia, arthralgia/bone pain, rash, hemorrhagic manifestation  Thrombocytopenia <101-150k cells/mm^3^  Increase in Hct 5-10%  No evidence of plasma leakage on exam or laboratory testing. |
| *Intermediate* | Dengue Hemorrhagic Fever (Grade 1-2):  Fever/History of fever  Evidence of hemorrhage  Thrombocytopenia  Plasma leakage: ≥20% increase in Hct OR ≥20% decrease in Hct following fluid resuscitation OR hypoproteinemia OR pleural effusions / ascites  Grade 1: Fever accompanied by non-specific constitutional symptoms; the only hemorrhagic manifestation is a positive tourniquet test and/or easy bruising.  Grade 2: Spontaneous bleeding in addition to the manifestations of Grade I patients, usually in the forms of skin or other hemorrhages. | Dengue with Warning Signs:  Abdominal pain / tenderness  Persistent vomiting  Clinical fluid accumulation  Mucosal bleed  Lethargy, restlessness  Liver enlargement >2cm  Increase in Hct with rapid decrease in platelets | Dengue Hemorrhagic Fever Grade 1-2:  Thrombocytopenia <100k cells/mm^3^  Rise in Hct ≥20%  DHF Grade 1:  Fever and hemorrhagic manifestation  (positive tourniquet test) and evidence of plasma leakage  DHF Grade 2:  Grade 1 + spontaneous bleeding |
| *Severe Disease* | DHF Grade 3-4 aka Dengue Shock Syndrome:  Circulatory failure manifested by a rapid, weak pulse and narrowing of pulse pressure.  Or in lieu of narrow pulse pressure, could have hypotension the presence of cold,  clammy skin and restlessness. | Severe Dengue:  Severe plasma leakage: shock or fluid accumulation with respiratory distress  Severe bleeding  Severe organ involvement with transaminase ≥1000, impaired consciousness, or “heart or other organs” | DHF Grade 3-4 aka Dengue Shock Syndrome:  DHF Grade 3:  Circulatory failure as manifested by weak pulse, narrow pulse pressure, hypotension, restlessness  DHF Grade 4:  Undetectable BP and pulses |

**References**

1.	World Health Organization. Dengue hemorrhagic fever: Diagnosis, treatment, prevention and control. Second Edition. 1997.

2.	World Health Organization. Dengue Guidelines for Diagnosis, Treatment, Prevention and Control. New edition 2009. 2009.

3.	World Health Organization Regional Office for South-East Asia. Comprehensive Guidelines for Prevention and Control of Dengue and Dengue Hemorrhagic Fever. Revised and expanded edition. 2011.
