# Supplementary material for: A systematic review of ultrasound to predict severe disease in patients with dengue and other viral infections associated with hemorrhagic fevers
Source: PLoS Negl Trop Dis. 2026 Sep 17;20(9):e0014609. doi: 10.1371/journal.pntd.0014609 (PMC13585234; doi:10.1371/journal.pntd.0014609)
Supplement: Supplementary file 4 [file pntd.0014609.s004.docx]

Supporting Information 4: Publications included in the systematic review. Enrollment design refers to whether participant enrollment 1) was prospective or retrospective, 2) if patients were recruited in consecutive, convenience, or another manner. CCHF: Crimean-Congo hemorrhagic fever. N/A: not applicable. NS: Not specified. DF: dengue fever. DHF: dengue hemorrhagic fever. DWS: dengue with warning signs. DSS: dengue shock syndrome. POCUS: Point-of-care ultrasound. *Range provided for inclusion/recruitment, but actual age range of participants not provided. **Used 3mm and 5mm, and 3mm was the cutoff used in our analyses.

| **Extracted Data** | | | | | | | | | | | | | | **QUADAS** | | | | | | |
| --- | --- | --- | --- | --- | --- | --- | --- | --- | --- | --- | --- | --- | --- | --- | --- | --- | --- | --- | --- | --- |
| **Study Information** | | | | | | | | **Recruitment and Participant Information** | | | | **Cutoffs** | | **Patient Selection** | | **Ultrasound Test** | | **Outcome Assessment** | | **Flow and Timing** |
| **Virus** | **Year** | **Authors** | **Country** | **Inclusion Type** | **Definition of Severity** | **n** | **Sonographer** | **Setting** | **Enrollment**  **Design** | **Percent with Ultrasound** | **Participant Age Group** | **GBWT** | **Splenomegaly** | **Bias** | **Applicability** | **Bias** | **Applicability** | **Bias** | **Applicability** | **Bias** |
| CCHF | 2009 | Engin A,  et al.[1] | Turkey | Predictive | Per Swanepoel et al. 1989[2] | 44 | Echocardiographer | Inpatient | Prospective, Consecutive | 100% | Adult  Range not provided | - | - | Low | Low | Low | Low | Low | Low | Low |
| CCHF | 2011 | Gul I,  et al.[3] | Turkey | Descriptive | - | 23 | Echocardiographer | Inpatient | Prospective, Consecutive | 100% | Pediatric/Adolescent  9 – 16 years | - | - | Low | Low | Low | Low | N/A | N/A | N/A |
| CCHF | 2014 | Tufan ZK,  et al.[4] | Turkey | Descriptive | - | 210 | Radiologists | Inpatient | Prospective,  NS | 100% | Adult  18 – 81 years | ≥2.5 mm | NS | Unclear | Low | Low | Low | N/A | N/A | N/A |
| CCHF | 2014 | Ziraman I,  et al.[5] | Turkey | Predictive | Severity Scoring Index per Dokuzoguz et al. 2013[6] | 25 | Radiologists | Inpatient | Prospective,  NS | 100% | Adult/Adolescent  15 – 80 years | NS | NS | Unclear | Low | Low | Low | Low | Low | Low |
| CCHF | 2019 | Karavaş E,  et al.[7] | Turkey | Predictive | Per Bakir et al.[8,9] | 48 | Radiologist | Inpatient | Prospective,  NS | 100% | Adult  19 – 79 years | NS | >12 cm | Unclear | Low | Low | Low | Low | Low | Low |
| CCHF | 2020 | Osquee HO,  et al.[10] | Iran | Predictive | Death | 144 | NS | Inpatient | NS | 90% | Not specified  (included adults) | NS | NS | Unclear | Low | Low | Low | Low | Low | Low |
| Dengue | 1991 | Pramuljo HS,  et al.[11] | Indonesia | Descriptive | - | 25 | NS | Inpatient | NS | 100% | Pediatric/Adolescent  2 – 13 years | NS | - | Unclear | Low | Low | Low | N/A | N/A | N/A |
| Dengue | 1998 | Setiawan MW,  et al.[12] | Indonesia | Predictive | WHO 1986[13]  DHF I/II vs  DHF III/IV | 148 | NS | Inpatient | Prospective,  NS | 100% | Pediatric/Adolescent  5 months – 14 years | >3 mm | Indexed to height | Unclear | Low | Low | Low | Low | Low | High |
| Dengue | 2004 | Wu KL,  et al.[14] | Taiwan | Descriptive | - | 65 | NS | Inpatient | Prospective,  NS | 100% | Adult  18 – 76 years | >3.5 mm | Index >20cm | Unclear | Low | Low | Low | N/A | N/A | N/A |
| Dengue | 2005 | Venkata Sai PM,  et al.[15] | India | Descriptive | - | 88 | NS | NS | Retrospective, NS | 100% | Pediatric  2 – 9 years | >2 mm | NS | Unclear | Low | Low | Low | N/A | N/A | N/A |
| Dengue | 2006 | Balasubramanian S,  et al.[16] | India | Predictive | WHO 1999[17]  DF vs DHF | 65 | NS | Inpatient | Prospective,  NS | 100% | Pediatric/Adolescent  >12 months – 18 years* | NS | NS | Unclear | Low | Low | Low | Low | Low | Unclear |
| Dengue | 2006 | Quiroz-Moreno R,  et al.[18] | Mexico | Descriptive | - | 132 | NS | Inpatient | Prospective,  NS | 100% | Adult/Adolescent  16 – 76 years | **>3 mm /  >5 mm | NS | Unclear | Low | Unclear | Low | N/A | N/A | N/A |
| Dengue | 2007 | Brito CA,  et al.[19] | Brazil | Descriptive | - | 14 | NS | Inpatient | NS | 100% | Adult  18 – 82 years | ≥5 mm | - | Unclear | Low | Low | Low | N/A | N/A | N/A |
| Dengue | 2007 | Srikiatkhachorn A,  et al.[20] | Thailand | Predictive | Per Nimmannitya et al. 1993[21]  DF vs DHF/DSS | 82 | Radiologists | Inpatient | Prospective,  NS | 100% | Pediatric/Adolescent  <15 years* | >2 mm | - | Unclear | Low | Low | Low | Low | Low | Low |
| Dengue | 2010 | Oliveira GA,  et al.[22] | Brazil | Descriptive | - | 37 | Radiology resident with pediatric sonographer | Inpatient | NS | 100% | Pediatric/Adolescent  1 month – 17 years | ≥3 mm | - | Unclear | Low | Low | Low | N/A | N/A | N/A |
| Dengue | 2011 | Motla M,  et al.[23] | India | Descriptive | - | 73 | Radiologist | Inpatient | Retrospective,  NS | 100% | All  3 – 75 years | >3 mm | NS | Unclear | Low | Low | Low | N/A | N/A | N/A |
| Dengue | 2012 | Jagadishkumar K,  et al.[24] | India | Predictive | WHO 1997  DF vs DHS/DSS | 110 | NS | NS | Prospective, Consecutive | 100% | Pediatric/Adolescent  2 months – 14 years | >5 mm | - | Low | Low | Low | Low | Low | Low | Unclear |
| Dengue | 2012 | Meltzer E,  et al.[25] | Multiple | Descriptive | - | 35 | NS | Inpatient | Prospective,  NS | 100% | Not specified but included adults | NS | - | Unclear | Low | Low | Low | N/A | N/A | N/A |
| Dengue | 2013 | Bharath KR,  et al.[26] | India | Predictive | Death | 324 | Senior radiologist | Inpatient | Prospective,  NS | 100% | Pediatric/Adolescent  <12 months – 18 years | >3 mm | NS | Unclear | Low | Low | Low | Low | Low | Unclear |
| Dengue | 2013 | Michels M,  et al.[27] | Indonesia | Predictive | WHO 2009  Severe vs Non-severe | 66 | "Study physicians" who received, radiologist | Inpatient | Prospective,  NS | 100% | Adult/Adolescent  ≥14 years* | ≥3 mm | - | Unclear | Low | Low | Low | Low | Low | High |
| Dengue | 2015 | Arshad K,  et al.[28] | Pakistan | Descriptive | - | 93 | "Radiology department" | Inpatient | Prospective, Consecutive | 100% | Pediatric/Adolescent  4 – 14 years | - | NS | Low | Low | Low | Low | N/A | N/A | N/A |
| Dengue | 2015 | Taylor WR,  et al.[29] | Vietnam | Descriptive | - | 129 | Attending physicians | Inpatient | Prospective,  NS | 90% | Adult/Adolescent  16 – 72 years | - | - | Unclear | Low | Unclear | Low | N/A | N/A | N/A |
| Dengue | 2016 | Arora M,  et al.[30] | India | Descriptive | - | 120 | NS | Inpatient | NS | 100% | Adult  >18 years* | - | - | Unclear | Low | Low | Low | N/A | N/A | N/A |
| Dengue | 2016 | Bandyopadhyay D,  et al.[31] | India | Predictive | DF vs DHF/DSS | 110 | NS | Inpatient | Prospective,  NS | 100% | Adult/Adolescent  ≥14 years* | NS | - | Unclear | Low | Low | Low | Unclear | Low | Unclear |
| Dengue | 2016 | Khurram M,  et al.[32] | Pakistan | Predictive | DHF vs DSS | 240 | Sonographer | NS | NS | 100% | All  Range not provided | >3 mm | - | Unclear | Low | Low | Low | Low | Low | Unclear |
| Dengue | 2016 | Pothapregada S,  et al.[33] | India | Predictive | WHO-SEA 2011  Severe vs Non-severe | 254 | Radiologist | Inpatient | Retrospective, Consecutive | 100% | Pediatric  Range not provided | >3 mm | Per Konus et al. 1998[34] | Low | Low | Low | Low | Low | Low | High |
| Dengue | 2017 | Parmar J,  et al.[35] | India | Predictive | WHO 2009  Severe vs Non-severe | 93 | Radiologist | Emergency and medicine departments | Prospective,  NS | 100% | All  1 – 77 years | ≥3 mm | Adults: >12 cm  Pediatrics: Indexed to height/weight | Unclear | Low | Low | Low | N/A | N/A | Unclear |
| Dengue | 2017 | Ramabhatta S,  et al.[36] | India | Predictive | WHO 2012  Severe vs Non-severe | 568 | NS | Inpatient | Prospective,  NS | 100% | Pediatric/Adolescent  “Infancy” – 17 years | - | - | Unclear | Low | Low | Low | Low | Low | Unclear |
| Dengue | 2018 | Nainggolan L,  et al.[37] | Indonesia | Predictive | WHO 1997  DF vs DHF based on presence of plasma leakage | 69 | Sonographer | Inpatient | Prospective, Consecutive | 100% | Adult  Range not provided | >3 mm | - | Low | Low | Low | Low | Low | Low | Unclear |
| Dengue | 2019 | Datta G,  et al.[38] | India | Descriptive | - | 120 | NS | Inpatient | Prospective,  NS | 100% | Adult/Adolescent  13 – 74 years | - | - | Unclear | Low | Low | Low | N/A | N/A | N/A |
| Dengue | 2019 | Ferreira BDC,  et al.[39] | Brazil | Descriptive | - | 51 | Radiologist | Referred from emergency department | Prospective, Convenience | 100% | Adult  ≥18 years* | >3 mm | >20 cm^3^ | Unclear | Low | Low | Low | N/A | N/A | N/A |
| Dengue | 2019 | Mohanty B,  et al.[40] | India | Descriptive | - | 520 | NS | Inpatient | Prospective,  NS | 100% | Adult/Adolescent  12 – 76 years | NS | - | Unclear | Low | Low | Low | N/A | N/A | N/A |
| Dengue | 2019 | Parmar J,  et al.[41] | India | Predictive | WHO 2009  Severe vs Non-severe | 244 | Radiologist | Inpatient | Prospective,  NS | 100% | All  1 -81 years | ≥3 mm | NS | Unclear | Low | Low | Low | Low | Low | Low |
| Dengue | 2019 | Tavares MA,  et al.[42] | Brazil | Predictive | WHO 2009  Severe vs DWS | 118 | Physician | Emergency department | Retrospective, Consecutive | 100% | Adult/Adolescent  13 – 84 years | >3 mm** /  > 5 mm | NS | Low | Low | Low | Low | Low | Low | High |
| Dengue | 2020 | Adil B,  et al.[43] | Pakistan | Predictive | WHO 1997  DF vs DHF/DSS | 180 | Radiologists | Inpatient | Prospective, Stratified | 100% | Adult/Adolescent  16 – 73 years | >3 mm | >12 cm | Low | Low | Low | Low | Low | Low | Unclear |
| Dengue | 2020 | Dussart P,  et al.[44] | Cambodia | Descriptive | - | 173 | NS | Inpatient | Prospective,  NS | 100% | Pediatric  2 – 5 years | - | - | Unclear | Low | Low | Low | N/A | N/A | N/A |
| Dengue | 2020 | Rathore APS,  et al.[45] | Sri Lanka | Predictive | WHO 1997  DF vs DHF | 84 | "Per hospital standard protocol" | Inpatient | Prospective,  NS | 100% | Pediatric  ≤12 years* | NS | - | Unclear | Low | Low | Low | Low | Low | Unclear |
| Dengue | 2020 | Xin Tian C,  et al.[46] | Malaysia | Descriptive | - | 83 | Single operator, received one month training | Emergency department and outpatient | Prospective,  NS | 100% | Adult  18 – 72 years | ≥3 mm | - | Unclear | Low | Low | Low | N/A | N/A | N/A |
| Dengue | 2021 | Abhinayaa J,  et al.[47] | India | Predictive | WHO 2009  Severe vs Non-severe | 130 | NS | Inpatient | Prospective,  NS | 100% | Pediatric  1 month – 15 years* | - | - | Unclear | Low | Low | Low | Low | Low | Unclear |
| Dengue | 2021 | Donaldson CD,  et al.[48] | Sri Lanka | Predictive | WHO 2009  Severe vs Non-severe | 176 | Radiologists | Inpatient | Prospective,  NS | 100% | Adult  18 – 72 years | NS | >12 cm | Unclear | Low | Low | Low | Low | Low | Low |
| Dengue | 2021 | Koyama H,  et al.[49] | Thailand | Predictive | WHO  Dengue vs DWS | 50 | “Experienced lung ultrasound examiner” | Inpatient | Prospective, Consecutive | 100% | Adult/Adolescent  ≥15 years | - | - | Low | Low | Low | Low | Low | Low | Unclear |
| Dengue | 2021 | Mansanguan C,  et al.[50] | Thailand | Predictive | WHO 1997  DF vs DHF/DSS | 81 | Cardiologist | Inpatient | Prospective,  NS | 100% | Adult  20 – 46 years | - | - | Unclear | Low | Low | Low | Low | Low | Low |
| Dengue | 2021 | Sigera PC,  et al.[51] | Sri Lanka | Predictive | WHO 2009 / Navares et al. 2011[52]  Compensated vs uncompensated shock | 426 | Medical or radiology registrars, senior house officers, consultant radiologists or physicians | Inpatient | Prospective,  NS | 100% | Adult  ≥18 years* | >3 mm  (but results not included) | - | Unclear | Low | Low | Low | Low | Low | Low |
| Dengue | 2022 | Ibrahim MA,  et al.[53] | Malaysia | Predictive | WHO 2009  DWS vs Severe dengue | 44 | POCUS fellowship trained principal investigator | Emergency department and inpatient | Prospective, Convenience | 100% | Adult  Range not provided | ≥3 mm | - | Unclear | Low | Low | Low | Low | Low | High |
| Dengue | 2022 | Nerella S,  et al.[54] | India | Predictive | National Guidelines 2025  Severe vs Non-severe | 150 | Cardiologist | Inpatient | Prospective,  NS | 100% | Pediatric  1 month – 12 years | - | - | Unclear | Low | Low | Low | Low | Low | Unclear |
| Dengue | 2022 | Riswari SF,  et al.[55] | Indonesia | Predictive | WHO 1997  DF vs DHF | 48 | Attending clinicians | Inpatient | Prospective,  NS | 100% | Adult/Adolescent  ≥14 years* | >3 mm | - | Unclear | Low | Low | Low | Low | Low | High |
| Dengue | 2022 | Uthraraj NS,  et al.[56] | India | Descriptive | - | 52 | Radiologist | Inpatient | Retrospective,  NS | 100% | Pediatric/Adolescent  ≤15 years* | >5 mm | - | Unclear | Low | Low | Low | N/A | N/A | N/A |
| Dengue | 2023 | Chaudhary S,  et al.[57] | India | Predictive | Dengue Infection Severity Score[58]  DF vs DHS/DSS | 418 | Radiologists | Inpatient | Prospective,  “All patients" | 100% | All  <15 – >60 years | >3 mm | >13 cm | Low | Low | Low | Low | Low | Low | Unclear |
| Dengue | 2023 | Lakra MS,  et al.[59] | India | Descriptive | - | 37 | NS | Inpatient | Prospective,  NS | 37% | Pediatric/Adolescent  3 months – 14 years* | NS | NS | High | Low | Low | Low | N/A | N/A | N/A |
| Dengue | 2023 | Saghir H,  et al.[60] | Pakistan | Descriptive | - | 64 | NS | Outpatient | Retrospective,  NS | NS | Not Specified  (included adults) | NS | - | Unclear | Low | Unclear | Low | N/A | N/A | N/A |
| Hanta | 2001 | Kim YO,  et al.[61] | Korea | Predictive | Death | 68 | NS | Inpatient | Retrospective,  NS | 100% | Adult/Adolescent  10 – 76 years | ≥4 mm | NS | Unclear | Low | Unclear | Low | Low | Low | Low |
| Hanta | 2002 | Paakkala A,  et al.[62] | Finland | Descriptive | - | 23 | Radiologist | Inpatient | Prospective, Consecutive | 100% | Adult/Adolescent  17 – 66 years | - | - | Low | Low | Low | Low | N/A | N/A | N/A |
| Hanta | 2009 | Makela S,  et al.[63] | Finland | Descriptive | - | 43 | NS | Inpatient | Prospective, Consecutive | 61% | Adult/Adolescent  15 – 70 years | - | - | High | Low | Low | Low | N/A | N/A | N/A |
| Lassa | 2023 | Erameh CO,  et al.[64] | Nigeria | Predictive | Death | 46 | General physicians who received training | Inpatient | Prospective,  NS | 100% | Adult/Adolescent  16 – 72 years | - | - | Unclear | Low | Low | Low | Low | Low | Low |
| Yellow Fever | 2019 | Paixao GMM,  et al.[65] | Brazil | Predictive | Per Litvoc et al. 2018[66]  Mild/moderate vs Severe | 70 | "Nonphysicians" | Inpatient | Prospective, Consecutive | 100% | Not specified  (included adults) | - | - | Low | Low | Low | Low | Low | Low | Low |
| Yellow Fever | 2021 | Neves YCS,  et al.[67] | Brazil | Predictive | Death | 46 | Radiologist | Inpatient | Retrospective,  NS | NS | Adult  19 – 75 years | >3 mm | - | Unclear | Low | Low | Low | Low | Low | Low |

**References**

1.	Engin A, Yilmaz MB, Elaldi N, Erdem A, Yalta K, Tandogan I, et al. Crimean-Congo hemorrhagic fever: does it involve the heart? International journal of infectious diseases : IJID : official publication of the	International Society for Infectious Diseases. 2009;13: 369–73. doi:10.1016/j.ijid.2008.07.019

2.	Swanepoel R, Gill DE, Shepherd AJ, Leman PA, Mynhardt JH, Harvey S. The clinical pathology of Crimean-Congo hemorrhagic fever. Rev Infect Dis. 1989;11 Suppl 4: S794-800. doi:10.1093/clinids/11.supplement_4.s794

3.	Gul I, Kaya A, Güven AS, Karapinar H, Küçükdurmaz Z, Yilmaz A, et al. Cardiac findings in children with Crimean-Congo hemorrhagic fever. Medical science monitor : international medical journal of experimental and	clinical research. 2011;17: CR457-460. doi:10.12659/MSM.881907

4.	Tufan ZK, Yigit H, Kacar M, Bulut C, Canpolat G, Hatipoglu CA, et al. Sonographic findings in patients with Crimean-Congo hemorrhagic fever. Journal of ultrasound in medicine : official journal of the American Institute of	Ultrasound in Medicine. 2014;33: 1999–2003. doi:10.7863/ultra.33.11.1999

5.	Ziraman I, Celikbas A, Ergonul O, Degirmenci T, Uyanik SA, Koparal S, et al. Crimean-Congo hemorrhagic fever: aid of abdominal ultrasonography in prediction of severity. Vector borne and zoonotic diseases (Larchmont, NY). 2014;14: 817–20. doi:10.1089/vbz.2014.1662

6.	Dokuzoguz B, Celikbas AK, Gök ŞE, Baykam N, Eroglu MN, Ergönül Ö. Severity Scoring Index for Crimean-Congo Hemorrhagic Fever and the Impact of Ribavirin and Corticosteroids on Fatality. Clinical Infectious Diseases. 2013;57: 1270–1274. doi:10.1093/cid/cit527

7.	Karavaş E, Karakeçili F. Evaluation of Portal Vein Doppler Findings in Patients with Crimean^−^Congo Hemorrhagic Fever. Medicina (Kaunas, Lithuania). 2019;55. doi:10.3390/medicina55050146

8.	Bakir M, Engin A, Gozel MG, Elaldi N, Kilickap S, Cinar Z. A new perspective to determine the severity of cases with Crimean-Congo hemorrhagic fever. J Vector Borne Dis. 2012;49: 105–110.

9.	Bakır M, Gözel MG, Köksal I, Aşık Z, Günal Ö, Yılmaz H, et al. Validation of a severity grading score (SGS) system for predicting the course of disease and mortality in patients with Crimean-Congo hemorrhagic fever (CCHF). Eur J Clin Microbiol Infect Dis. 2015;34: 325–330. doi:10.1007/s10096-014-2238-0

10.	Osquee HO, Taghizadeh S, Haghdoost M, Pourjafar H, Ansari F. New Insight for the Prognosis of CCHF: Clinical, Laboratory and Sonography Findings. Current medical imaging. 2020;16: 1125–1130. doi:10.2174/1573405615666191111115354

11.	Pramuljo HS, Harun SR. Ultrasound findings in dengue haemorrhagic fever. Pediatric radiology. 1991;21: 100–2. doi:10.1007/BF02015615

12.	Setiawan MW, Samsi TK, Wulur H, Sugianto D, Pool TN. Dengue haemorrhagic fever: ultrasound as an aid to predict the severity of the disease. Pediatric radiology. 1998;28: 1–4. doi:10.1007/s002470050281

13.	World Health Organization. Dengue Hemorrhagic Fever: Diagnosis, Treatment and Control. Geneva; 1986.

14.	Wu KL, Changchien CS, Kuo CH, Chiu KW, Lu SN, Kuo CM, et al. Early abdominal sonographic findings in patients with dengue fever. Journal of clinical ultrasound : JCU. 2004;32: 386–8. doi:10.1002/jcu.20060

15.	Venkata Sai PM, Dev B, Krishnan R. Role of ultrasound in dengue fever. The British journal of radiology. 2005;78: 416–8. doi:10.1259/bjr/54704044

16.	Balasubramanian S, Janakiraman L, Kumar SS, Muralinath S, Shivbalan S. A reappraisal of the criteria to diagnose plasma leakage in dengue hemorrhagic fever. Indian pediatrics. 2006;43: 334–9.

17.	World Health Organziation SEARO. Prevention and control of Dengue and Dengue hemorrhagic fever: Comprehensive guidelines. WHO Regional Publication, SEARO No.29. New Delhi; 1999.

18.	Quiroz-Moreno R, Méndez GF, Ovando-Rivera KM. [Clinical utility of ultrasound in the identification of dengue hemorrhagic fever]. Revista medica del Instituto Mexicano del Seguro Social. 2006;44: 243–8.

19.	Brito CA, Albuquerque Mde F, Lucena-Silva N. [Plasma leakage detection in severe dengue: when serum albumin quantification plays a role?]. Revista da Sociedade Brasileira de Medicina Tropical. 2007;40: 220–3. doi:10.1590/s0037-86822007000200015

20.	Srikiatkhachorn A, Krautrachue A, Ratanaprakarn W, Wongtapradit L, Nithipanya N, Kalayanarooj S, et al. Natural history of plasma leakage in dengue hemorrhagic fever: a serial ultrasonographic study. Pediatr Infect Dis J. 2007;26: 283–290; discussion 291-292. doi:10.1097/01.inf.0000258612.26743.10

21.	Nimmannitya S. Clinical manifestations of dengue/dengue haemorrhagic fever. In: Monograph on Dengue/Dengue Haemorrhagic Fever. 1993 pp. 48–61.

22.	Oliveira GA, Machado RC, Horvat JV, Gomes LE, Guerra LR, Vandesteen L, et al. Transient reticular gallbladder wall thickening in severe dengue fever: a reliable sign of plasma leakage. Pediatric radiology. 2010;40: 720–4. doi:10.1007/s00247-009-1489-x

23.	Motla M, Manaktala S, Gupta V, Aggarwal M, Bhoi SK, Aggarwal P, et al. Sonographic evidence of ascites, pleura-pericardial effusion and gallbladder wall edema for dengue fever. Prehospital and disaster medicine. 2011;26: 335–41. doi:10.1017/S1049023X11006637

24.	Jagadishkumar K, Jain P, Manjunath VG, Umesh L. Hepatic involvement in dengue Fever in children. Iranian journal of pediatrics. 2012;22: 231–6.

25.	Meltzer E, Heyman Z, Bin H, Schwartz E. Capillary leakage in travelers with dengue infection: implications for pathogenesis. The American journal of tropical medicine and hygiene. 2012;86: 536–9. doi:10.4269/ajtmh.2012.10-0670

26.	Bharath Kumar Reddy KR, Laksmana RR, Veerappa BG, Shivananda. Ultrasonography as a tool in predicting the severity of dengue fever in children--a useful aid in a developing country. Pediatric radiology. 2013;43: 971–7. doi:10.1007/s00247-013-2642-0

27.	Michels M, Sumardi U, de Mast Q, Jusuf H, Puspita M, Dewi IM, et al. The predictive diagnostic value of serial daily bedside ultrasonography for severe dengue in Indonesian adults. PLoS neglected tropical diseases. 2013;7: e2277. doi:10.1371/journal.pntd.0002277

28.	Arshad K, Sheikh S, Naqvi SU, Sarwar I, Javaid S, Asghar M, et al. FREQUENCY OF SPLENOMEGALY IN DENGUE FEVER IN CHILDREN. Journal of Ayub Medical College, Abbottabad : JAMC. 2015;27: 356–9.

29.	Taylor WR, Fox A, Pham KT, Le HNM, Tran NTH, Tran GV, et al. Dengue in adults admitted to a referral hospital in Hanoi, Vietnam. The American journal of tropical medicine and hygiene. 2015;92: 1141–1149. doi:10.4269/ajtmh.14-0472

30.	Arora M, Patil RS. Cardiac Manifestation in Dengue Fever. The Journal of the Association of Physicians of India. 2016;64: 40–44.

31.	Bandyopadhyay D, Chattaraj S, Hajra A, Mukhopadhyay S, Ganesan V. A Study on Spectrum of Hepatobiliary Dysfunctions and Pattern of Liver Involvement in Dengue Infection. Journal of clinical and diagnostic research : JCDR. 2016;10: OC21-6. doi:10.7860/JCDR/2016/16946.7784

32.	Khurram M, Qayyum W, Umar M, Jawad M, Mumtaz S, Bushra Khaar HT. Ultrasonographic pattern of plasma leak in dengue haemorrhagic fever. JPMA The Journal of the Pakistan Medical Association. 2016;66: 260–4.

33.	Pothapregada S, Kullu P, Kamalakannan B, Thulasingam M. Is Ultrasound a Useful Tool to Predict Severe Dengue Infection? Indian journal of pediatrics. 2016;83: 500–4. doi:10.1007/s12098-015-2013-y

34.	Konuş OL, Ozdemir A, Akkaya A, Erbaş G, Celik H, Işik S. Normal liver, spleen, and kidney dimensions in neonates, infants, and children: evaluation with sonography. American Journal of Roentgenology. 1998;171: 1693–1698. doi:10.2214/ajr.171.6.9843315

35.	Parmar JP, Mohan C, Vora M. Patterns of Gall Bladder Wall Thickening in Dengue Fever: A Mirror of the Severity of Disease. Ultrasound international open. 2017;3: E76–E81. doi:10.1055/s-0043-105262

36.	Ramabhatta S, Palaniappan S, Hanumantharayappa N, Begum SV. The Clinical and Serological Profile of Pediatric Dengue. Indian journal of pediatrics. 2017;84: 897–901. doi:10.1007/s12098-017-2423-0

37.	Nainggolan L, Wiguna C, Hasan I, Dewiasty E. Gallbladder Wall Thickening for Early Detection of Plasma Leakage in Dengue Infected Adult Patients. Acta medica Indonesiana. 2018;50: 193–199.

38.	Datta G, Mitra P. A Study on Cardiac Manifestations of Dengue Fever. The Journal of the Association of Physicians of India. 2019;67: 14–16.

39.	Ferreira BDC, Correia D. Ultrasound Assessment of Hepatobiliary and Splenic Changes in Patients With Dengue and Warning Signs During the Acute and Recovery Phases. Journal of ultrasound in medicine : official journal of the American Institute of	Ultrasound in Medicine. 2019;38: 2015–2024. doi:10.1002/jum.14890

40.	Mohanty B, Sunder A, Pathak S. Clinicolaboratory profile of expanded dengue syndrome - Our experience in a teaching hospital. Journal of family medicine and primary care. 2019;8: 1022–1027. doi:10.4103/jfmpc.jfmpc_12_19

41.	Parmar J, Vora M, Mohan C, Shah S, Mahajan H, Patel T. “Honeycomb” pattern of gallbladder wall thickening - A forward step in early diagnosis of “Severe Dengue Fever”. The Indian journal of radiology & imaging. 2019;29: 14–18. doi:10.4103/ijri.IJRI_363_18

42.	Tavares MA, João GAP, Bastos MS, Gimaque JBL, Almeida ACG, Ngo TT, et al. Clinical relevance of gallbladder wall thickening for dengue severity: A cross-sectional study. PloS one. 2019;14: e0218939. doi:10.1371/journal.pone.0218939

43.	Adil B, Rabbani A, Ahmed S, Arshad I Sr, Khalid MA. Gall Bladder Wall Thickening in Dengue Fever - Aid in Labelling Dengue Hemorrhagic Fever and a Marker of Severity. Cureus. 2020;12: e11331. doi:10.7759/cureus.11331

44.	Dussart P, Duong V, Bleakley K, Fortas C, Lorn Try P, Kim KS, et al. Comparison of dengue case classification schemes and evaluation of biological changes in different dengue clinical patterns in a longitudinal follow-up of hospitalized children in Cambodia. PLoS neglected tropical diseases. 2020;14: e0008603. doi:10.1371/journal.pntd.0008603

45.	Rathore APS, Senanayake M, Athapathu AS, Gunasena S, Karunaratna I, Leong WY, et al. Serum chymase levels correlate with severe dengue warning signs and clinical fluid accumulation in hospitalized pediatric patients. Scientific reports. 2020;10: 11856. doi:10.1038/s41598-020-68844-z

46.	Xin Tian C, Baharuddin KA, Shaik Farid AW, Andey R, Ridzuan MI, Siti-Azrin AH. Ultrasound findings of plasma leakage as imaging adjunct in clinical management of dengue fever without warning signs. The Medical journal of Malaysia. 2020;75: 635–641.

47.	Abhinayaa J, James S, Jebaraj R, Vinoth PN. Incidence of Cardiac Manifestations in Children with Dengue Fever: A Cross-sectional Study. Rambam Maimonides medical journal. 2021;12. doi:10.5041/RMMJ.10436

48.	Donaldson CD, de Mel S, Clarice CSH, Thilakawardana BU, de Mel P, Shalindi M, et al. Admission ultrasonography as a predictive tool for thrombocytopenia and disease severity in dengue infection. Transactions of the Royal Society of Tropical Medicine and Hygiene. 2021;115: 1396–1402. doi:10.1093/trstmh/trab064

49.	Koyama H, Chierakul W, Charunwatthana P, Sanguanwongse N, Phonrat B, Silachamroon U, et al. Lung Ultrasound Findings of Patients with Dengue Infection: A Prospective Observational Study. The American journal of tropical medicine and hygiene. 2021;105: 766–770. doi:10.4269/ajtmh.20-1274

50.	Mansanguan C, Hanboonkunupakarn B, Muangnoicharoen S, Huntrup A, Poolcharoen A, Mansanguan S, et al. Cardiac evaluation in adults with dengue virus infection by serial echocardiography. BMC infectious diseases. 2021;21: 940. doi:10.1186/s12879-021-06639-x

51.	Sigera PC, Weeratunga P, Deepika Fernando S, Lakshitha De Silva N, Rodrigo C, Rajapakse S. Rational use of ultrasonography with triaging of patients to detect dengue plasma leakage in resource limited settings: a prospective cohort study. Tropical medicine & international health : TM & IH. 2021;26: 993–1001. doi:10.1111/tmi.13594

52.	Narvaez F, Gutierrez G, Pérez MA, Elizondo D, Nuñez A, Balmaseda A, et al. Evaluation of the Traditional and Revised WHO Classifications of Dengue Disease Severity. Hirayama K, editor. PLoS Negl Trop Dis. 2011;5: e1397. doi:10.1371/journal.pntd.0001397

53.	Ibrahim MA, Hamzah SS, Md Noor J, Mohamad MIK, Mokhtar MF, Isa MR, et al. The association of ultrasound assessment of gallbladder wall thickness with dengue fever severity. The ultrasound journal. 2022;14: 13. doi:10.1186/s13089-022-00262-w

54.	Nerella S, Sarkar UK, Namdeo H. Electrocardiographic and echocardiographic findings in children with dengue infection. Journal of family medicine and primary care. 2022;11: 2334–2339. doi:10.4103/jfmpc.jfmpc_1280_21

55.	Riswari SF, Budiman MF, Darmayanti D, Ernawati, Prodjosoewojo S, Susandi E, et al. A Comparison of the Accuracy of Handheld Hemoglobinometer and Hematocrit Measurements for Detecting Plasma Leakage in Dengue Hemorrhagic Fever. International journal of general medicine. 2022;15: 2589–2595. doi:10.2147/IJGM.S343017

56.	Uthraraj NS, Sriraam LM, Hiriyur Prakash M, Kumar M, Palanisamy U, Chettiakkapalayam Venkatachalam KU. Predictive Factors for the Complications of Dengue Fever in Children: A Retrospective Analysis. Cureus. 2022;14: e33027. doi:10.7759/cureus.33027

57.	Chaudhary S, Manrai K, Dhagat P, Dudeja P, Sen D, Grewal DS, et al. Abdominal and Chest Ultrasonography: A predictor for disease progression in nonsevere dengue. Medical journal, Armed Forces India. 2023;79: 386–391. doi:10.1016/j.mjafi.2021.03.012

58.	Pongpan S, Wisitwong A, Tawichasri C, Patumanond J, Namwongprom S. Development of Dengue Infection Severity Score. ISRN Pediatrics. 2013;2013: 1–6. doi:10.1155/2013/845876

59.	Lakra MS, Karotkar SA, Lakhkar B, Dhawan V, Lakra A. To study ultrasound and chest X-ray findings and their role in the diagnosis of dengue fever in children. Journal of family medicine and primary care. 2023;12: 1938–1942. doi:10.4103/jfmpc.jfmpc_2325_22

60.	Saghir H, Tariq M, Safdar N, Zahoor A, Rashid F, Khan SE, et al. Association of Liver Function Test with Severity of Dengue Fever in Suburbs of Islamabad. Journal of community hospital internal medicine perspectives. 2023;13: 95–99. doi:10.55729/2000-9666.1250

61.	Kim YO, Chun KA, Choi JY, Yoon SA, Yang CW, Kim KT, et al. Sonographic evaluation of gallbladder-wall thickening in hemorrhagic fever with renal syndrome: prediction of disease severity. Journal of clinical ultrasound : JCU. 2001;29: 286–9. doi:10.1002/jcu.1035

62.	Paakkala A, Kallio T, Huhtala H, Apuli P, Paakkala T, Pasternack A, et al. Renal ultrasound findings and their clinical associations in nephropathia epidemica. Analysis of quantitative parameters. Acta radiologica (Stockholm, Sweden : 1987). 2002;43: 320–5. doi:10.1080/j.1600-0455.2002.430315.x

63.	Makela S, Kokkonen L, Ala-Houhala I, Groundstroem K, Harmoinen A, Huhtala H, et al. More than half of the patients with acute Puumala hantavirus infection have abnormal cardiac findings. Scandinavian journal of infectious diseases. 2009;41: 57–62. doi:10.1080/00365540802502629

64.	Erameh CO, Koch T, Edeawe OI, Oestereich L, Omansen T, Jochum J, et al. Focussed Assessment with Sonography in acute Lassa Fever (FASLa): Development of a point-of-care protocol and description of common ultrasound findings. The Journal of infection. 2023;87: 27–33. doi:10.1016/j.jinf.2023.04.008

65.	Paixão GMM, Nunes MCP, Beato BDVG, Sable C, Beaton AZ, Oliveira KKB, et al. Cardiac Involvement by Yellow Fever(from the PROVAR+ Study). The American journal of cardiology. 2019;123: 833–838. doi:10.1016/j.amjcard.2018.11.032

66.	Litvoc MN, Novaes CTG, Lopes MIBF. Yellow fever. Rev Assoc Med Bras (1992). 2018;64: 106–113. doi:10.1590/1806-9282.64.02.106

67.	Neves YCS, Castro-Lima VAC, Solla DJF, Ogata VSM, Pereira FL, Araujo JM, et al. Ultrasound Findings and Laboratory Predictors of Early Mortality in Patients With Severe Yellow Fever. AJR American journal of roentgenology. 2021;216: 1392–1399. doi:10.2214/AJR.20.23455
