# Supplementary material for: A systematic review of ultrasound to predict severe disease in patients with dengue and other viral infections associated with hemorrhagic fevers
Source: PLoS Negl Trop Dis. 2026 Sep 17;20(9):e0014609. doi: 10.1371/journal.pntd.0014609 (PMC13585234; doi:10.1371/journal.pntd.0014609)
Supplement: Supplementary file 5 [file pntd.0014609.s005.docx]

| **screeners** | **screening type** | **inclusion vs exclusion reason** | **year** | **authors** | **title** | **journal** | **volume** | **issue** | **pages** | **language** | **pubmed url** | **doi** |
| --- | --- | --- | --- | --- | --- | --- | --- | --- | --- | --- | --- | --- |
| TH/DZH | title/abstract | did not have primary data on US in VHF | 1963 | LINDENMANN J and LANE CA and HOBSON D | THE RESISTANCE OF A2G MICE TO MYXOVIRUSES. | Journal of immunology (Baltimore, Md. : 1950) | 90 |  | 942-51 | eng | https://pubmed.ncbi.nlm.nih.gov/14050204/ |  |
| TH/DZH | title/abstract | did not have primary data on US in VHF | 1963 | WENNER HA and LOU TY | VIRUS DISEASES ASSOCIATED WITH CUTANEOUS ERUPTIONS. | Progress in medical virology. Fortschritte der medizinischen Virusforschung. Progres en virologie medicale | 5 |  | 219-94 | eng | https://pubmed.ncbi.nlm.nih.gov/14159144/ |  |
| TH/DZH | title/abstract | did not have primary data on US in VHF | 1970 | Eggers HJ | [Viruses as causal agents of disease]. | Deutsche medizinische Wochenschrift (1946) | 95 | 9 | 473-7 | ger | https://pubmed.ncbi.nlm.nih.gov/4312733/ | 10.1055/s-0028-1108489 |
| TH/DZH | title/abstract | did not have primary data on US in VHF | 1973 | Gaƒ≠damovich SIa and Obukhova VR and Mel'nikova EE and Volokhova NA and Kiriushchenko TV | [Use of ultrasound for increasing the activity of arbovirus antigens in serological reactions in vitro]. | Voprosy virusologii | 18 | 3 | 356-60 | rus | https://pubmed.ncbi.nlm.nih.gov/4203622/ |  |
| TH/DZH | title/abstract | did not have primary data on US in VHF | 1973 | Wenner HA | Virus diseases associated with cutaneous eruptions. | Progress in medical virology. Fortschritte der medizinischen Virusforschung. Progres en virologie medicale | 16 |  | 269-336 | eng | https://pubmed.ncbi.nlm.nih.gov/4356897/ |  |
| TH/DZH | title/abstract | did not have primary data on US in VHF | 1977 | Inouye S and Matsuno S and Hasegawa A | Surface charge of dengue virus hemagglutinins prepared from infected mouse brains. | Japanese journal of medical science & biology | 30 | 2 | 109-18 | eng | https://pubmed.ncbi.nlm.nih.gov/916352/ | 10.7883/yoken1952.30.109 |
| TH/DZH | title/abstract | did not have primary data on US in VHF | 1979 | Shimshony A | RVF outbreak echoes biblical plague. | The Veterinary record | 104 | 22 | 511 | eng | https://pubmed.ncbi.nlm.nih.gov/384653/ | 10.1136/vr.104.22.511 |
| TH/DZH | title/abstract | did not have primary data on US in VHF | 1983 | Kirsi JJ and North JA and McKernan PA and Murray BK and Canonico PG and Huggins JW and Srivastava PC and Robins RK | Broad-spectrum antiviral activity of 2-beta-D-ribofuranosylselenazole-4-carboxamide, a new antiviral agent. | Antimicrobial agents and chemotherapy | 24 | 3 | 353-61 | eng | https://pubmed.ncbi.nlm.nih.gov/6615611/ | 10.1128/AAC.24.3.353 |
| TH/DZH | title/abstract | did not have primary data on US in VHF | 1988 | Petrichko MI and Glazun LO and Rodin AE | [Ultrasonic scanning of the kidneys in patients with hemorrhagic fever with renal syndrome and its complications]. | Klinicheskaia meditsina | 66 | 11 | 105-7 | rus | https://pubmed.ncbi.nlm.nih.gov/2907069/ |  |
| TH/DZH | title/abstract | did not have primary data on US in VHF | 1988 | Sirotin BZ and Maneshin VN and Shapiro IA and Voronina NV | [The importance of ultrasonography in detecting pathology of the kidneys during ambulatory observation of patients with hemorrhagic fever with renal syndrome]. | Klinicheskaia meditsina | 66 | 11 | 107-10 | rus | https://pubmed.ncbi.nlm.nih.gov/2907070/ |  |
| TH/DZH | title/abstract | US in VHF, but case study or series | 1989 | Pelupessy JM and Allo ER and Jota S | Pericardial effusion in dengue haemorrhagic fever. | Paediatrica Indonesiana | 29 | 3 | 72-5 | eng | https://pubmed.ncbi.nlm.nih.gov/2812820/ |  |
| TH/DZH | title/abstract | US in VHF, but case study or series | 1989 | Yanase O and Motomiya T and Watanabe K and Tokuyasu Y and Sakurada H and Tejima T and Hiyoshi Y and Sugiura M and Yabata Y and Kitazumi H | [Lassa fever associated with effusive constrictive pericarditis and bilateral atrioventricular annular constriction: a case report]. | Journal of cardiology | 19 | 4 | 1147-56 | jpn | https://pubmed.ncbi.nlm.nih.gov/2486633/ |  |
| TH/DZH | full text | full text not available | 1990 | Valiakhmetov RZ and Gafarov AI and Memkhes VS and Galimzianov VZ | [Spontaneous rupture of the kidneys in hemorrhagic fever with renal syndrome]. | Urologiia i nefrologiia |  | 6 | 50-3 | rus | https://pubmed.ncbi.nlm.nih.gov/1982474/ |  |
| TH/DZH | full text | only 38 of 113 patients were scanned with unclear mode of selection for ultrasound | 1990 | Wang LY and Chang WY and Lu SN and Chen TP | Sequential changes of serum transaminase and abdominal sonography in patients with suspected dengue fever. | Gaoxiong yi xue ke xue za zhi = The Kaohsiung journal of medical sciences | 6 | 9 | 483-9 | eng | https://pubmed.ncbi.nlm.nih.gov/2213970/ |  |
| TH/DZH | title/abstract | did not have primary data on US in VHF | 1991 | Paul R and Kiili√§inen H and Tarssanen L and Vorne M | [99Tcm]MAG3 gamma camera nephrography in epidemic nephritis. | Nuclear medicine communications | 12 | 1 | 15-25 | eng | https://pubmed.ncbi.nlm.nih.gov/1673780/ | 10.1097/00006231-199101000-00003 |
| TH/DZH | full text | INCLDUED | 1991 | Pramuljo HS and Harun SR | Ultrasound findings in dengue haemorrhagic fever. | Pediatric radiology | 21 | 2 | 100-2 | eng | https://pubmed.ncbi.nlm.nih.gov/2027705/ | 10.1007/BF02015615 |
| TH/DZH | title/abstract | did not have primary data on US in VHF | 1991 | Sabia P and Abbott RD and Afrookteh A and Keller MW and Touchstone DA and Kaul S | Importance of two-dimensional echocardiographic assessment of left ventricular systolic function in patients presenting to the emergency room with cardiac-related symptoms. | Circulation | 84 | 4 | 1615-24 | eng | https://pubmed.ncbi.nlm.nih.gov/1914101/ | 10.1161/01.cir.84.4.1615 |
| TH/DZH | title/abstract | did not have primary data on US in VHF | 1991 | Sabia P and Afrookteh A and Touchstone DA and Keller MW and Esquivel L and Kaul S | Value of regional wall motion abnormality in the emergency room diagnosis of acute myocardial infarction. A prospective study using two-dimensional echocardiography. | Circulation | 84 | 3 | I85-92 | eng | https://pubmed.ncbi.nlm.nih.gov/1884510/ |  |
| TH/DZH | title/abstract | did not have primary data on US in VHF | 1992 | Amvros'eva TV and Votiakov VI and Vladyko GV and Andreeva OT and Vervetchenko SG and Goretskaia IS and Klimashevskaia LM | [Antiviral properties of various pharmacologic groups of drugs]. | Antibiotiki i khimioterapiia = Antibiotics and chemoterapy [sic] | 37 | 11 | 05-Aug | rus | https://pubmed.ncbi.nlm.nih.gov/1338682/ |  |
| TH/DZH | title/abstract | did not have primary data on US in VHF | 1992 | Sabia PJ and Powers ER and Jayaweera AR and Ragosta M and Kaul S | Functional significance of collateral blood flow in patients with recent acute myocardial infarction. A study using myocardial contrast echocardiography. | Circulation | 85 | 6 | 2080-9 | eng | https://pubmed.ncbi.nlm.nih.gov/1591827/ | 10.1161/01.cir.85.6.2080 |
| TH/DZH | title/abstract | did not have primary data on US in VHF | 1992 | Sabia PJ and Powers ER and Ragosta M and Sarembock IJ and Burwell LR and Kaul S | An association between collateral blood flow and myocardial viability in patients with recent myocardial infarction. | The New England journal of medicine | 327 | 26 | 1825-31 | eng | https://pubmed.ncbi.nlm.nih.gov/1448120/ | 10.1056/NEJM199212243272601 |
| TH/DZH | title/abstract | did not have primary data on US in VHF | 1993 | Burke P and Di Virgilio MR and Luparia E and Piacenza M and Sabia A and Ba√π MG and Frigerio A | [Diagnostic imaging in non-palpable breast lesions. Targeted++ ultrasonography versus direct radiologic magnification]. | La Radiologia medica | 85 | 3 | 199-202 | ita | https://pubmed.ncbi.nlm.nih.gov/8493367/ |  |
| TH/DZH | title/abstract | did not have primary data on US in VHF | 1993 | Burkhardt U and Eggers HJ | [Viral infections--clinical pictures and laboratory diagnosis. Part 2: Coxsackie B virus, echovirus, enteroviruses, EBV, FSME virus and yellow fever virus]. | Fortschritte der Medizin | 111 | 32 | 510-2 | ger | https://pubmed.ncbi.nlm.nih.gov/8294077/ |  |
| TH/DZH | title/abstract | did not have primary data on US in VHF | 1993 | P√§iv√§nsalo M and Oikarinen H and Tikkakoski T and Puumala K and Suramo I | Radiological findings in bile duct carcinoma. | RoFo : Fortschritte auf dem Gebiete der Rontgenstrahlen und der Nuklearmedizin | 159 | 1 | 04-Sep | eng | https://pubmed.ncbi.nlm.nih.gov/8334256/ | 10.1055/s-2008-1032712 |
| TH/DZH | title/abstract | did not have primary data on US in VHF | 1993 | Ragosta M and Sabia PJ and Kaul S and DiMarco JP and Sarembock IJ and Powers ER | Effects of late (1 to 30 days) reperfusion after acute myocardial infarction on the signal-averaged electrocardiogram. | The American journal of cardiology | 71 | 1 | 19-23 | eng | https://pubmed.ncbi.nlm.nih.gov/8420230/ | 10.1016/0002-9149(93)90703-f |
| TH/DZH | title/abstract | did not have primary data on US in VHF | 1994 | Gowrishankar K and Rajajee S | Varied manifestations of viral myocarditis. | Indian journal of pediatrics | 61 | 1 | 75-80 | eng | https://pubmed.ncbi.nlm.nih.gov/7927602/ | 10.1007/BF02753563 |
| TH/DZH | title/abstract | did not have primary data on US in VHF | 1994 | Kaul S and Stratienko AA and Pollock SG and Marieb MA and Keller MW and Sabia PJ | Value of two-dimensional echocardiography for determining the basis of hemodynamic compromise in critically ill patients: a prospective study. | Journal of the American Society of Echocardiography : official publication of the American Society of Echocardiography | 7 | 6 | 598-606 | eng | https://pubmed.ncbi.nlm.nih.gov/7840987/ | 10.1016/s0894-7317(14)80082-5 |
| TH/DZH | title/abstract | did not have primary data on US in VHF | 1994 | Kontkanen M and Puustj√§rvi T and L√§hdevirta J | Myopic shift and its mechanism in nephropathia epidemica or Puumala virus infection. | The British journal of ophthalmology | 78 | 12 | 903-6 | eng | https://pubmed.ncbi.nlm.nih.gov/7819173/ | 10.1136/bjo.78.12.903 |
| TH/DZH | full text | full text not available | 1995 | Setiawan MW and Samsi TK and Pool TN and Sugianto D and Wulur H | Gallbladder wall thickening in dengue hemorrhagic fever: an ultrasonographic study. | Journal of clinical ultrasound : JCU | 23 | 6 | 357-62 | eng | https://pubmed.ncbi.nlm.nih.gov/7673451/ | 10.1002/jcu.1870230605 |
| TH/DZH | title/abstract | did not have primary data on US in VHF | 1996 | Kontkanen M and Puustj√§rvi T and Kauppi P and L√§hdevirta J | Ocular characteristics in nephropathia epidemica or Puumala virus infection. | Acta ophthalmologica Scandinavica | 74 | 6 | 621-5 | eng | https://pubmed.ncbi.nlm.nih.gov/9017055/ | 10.1111/j.1600-0420.1996.tb00748.x |
| TH/DZH | title/abstract | did not have primary data on US in VHF | 1997 | Di Pede F and Pucci PD and Zuin G and Cerisano GP and De Piccoli B and Taddeucci E and Turiano G and Sabia R and Piccolo E and Fazzini PF | Effects of transdermal nitroglycerin on left ventricular function after acute myocardial infarction. | Cardiologia (Rome, Italy) | 42 | 6 | 597-603 | eng | https://pubmed.ncbi.nlm.nih.gov/9234567/ |  |
| TH/DZH | full text | no patient level ultrasound data | 1997 | Linderholm M and Sandstr√∂m T and Rinnstr√∂m O and Groth S and Blomberg A and T√§rnvik A | Impaired pulmonary function in patients with hemorrhagic fever with renal syndrome. | Clinical infectious diseases : an official publication of the Infectious Diseases Society of America | 25 | 5 | 1084-9 | eng | https://pubmed.ncbi.nlm.nih.gov/9402362/ | 10.1086/516093 |
| TH/DZH | full text | no patient level ultrasound data | 1998 | Kabra SK and Juneja R and Madhulika and Jain Y and Singhal T and Dar L and Kothari SS and Broor S | Myocardial dysfunction in children with dengue haemorrhagic fever. | The National medical journal of India | 11 | 2 | 59-61 | eng | https://pubmed.ncbi.nlm.nih.gov/9624863/ |  |
| TH/DZH | title/abstract | did not have primary data on US in VHF | 1998 | Kontkanen M and Puustj√§rvi T | Hemorrhagic fever (Puumala virus infection) with ocular involvement. | Graefe's archive for clinical and experimental ophthalmology = Albrecht von Graefes Archiv fur klinische und experimentelle Ophthalmologie | 236 | 9 | 713-6 | eng | https://pubmed.ncbi.nlm.nih.gov/9782434/ | 10.1007/s004170050146 |
| TH/DZH | full text | INCLUDED: original patient level data on US in VHF | 1998 | Setiawan MW and Samsi TK and Wulur H and Sugianto D and Pool TN | Dengue haemorrhagic fever: ultrasound as an aid to predict the severity of the disease. | Pediatric radiology | 28 | 1 | 01-Apr | eng | https://pubmed.ncbi.nlm.nih.gov/9426264/ | 10.1007/s002470050281 |
| TH/DZH | full text | same data as Steiawan 1998 already included | 1998 | Setiawan MW and Samsi TK and Wulur H and Sugianto D and Pool TN | Epigastric pain and sonographic assessment of the pancreas in dengue hemorrhagic fever. | Journal of clinical ultrasound : JCU | 26 | 5 | 257-9 | eng | https://pubmed.ncbi.nlm.nih.gov/9608369/ | 10.1002/(sici)1097-0096(199806)26:5<257::aid-jcu5>3.0.co;2-b |
| TH/DZH | full text | only 17/108 patients received ultrasound with unclear selection | 1998 | Wali JP and Biswas A and Chandra S and Malhotra A and Aggarwal P and Handa R and Wig N and Bahl VK | Cardiac involvement in Dengue Haemorrhagic Fever. | International journal of cardiology | 64 | 1 | 31-6 | eng | https://pubmed.ncbi.nlm.nih.gov/9579814/ | 10.1016/s0167-5273(98)00008-4 |
| TH/DZH | title/abstract | did not have primary data on US in VHF | 1999 | Kabra SK and Jain Y and Singhal T and Ratageri VH | Dengue hemorrhagic fever: clinical manifestations and management. | Indian journal of pediatrics | 66 | 1 | 93-101 | eng | https://pubmed.ncbi.nlm.nih.gov/10798041/ | 10.1007/BF02752362 |
| TH/DZH | title/abstract | US in VHF, but case study or series | 1999 | Lauer B and Wiemer M and Kramer A and Pilaski J and Heering P and Horstkotte D and Schultheiss HP and Strauer BE | [Acute hantavirus infection caused by a genetically newly identified viral strain. Severe and complicated course of hemorrhagic fever with renal syndrome (nephropathia epidemica)]. | Medizinische Klinik (Munich, Germany : 1983) | 94 | 1 | 39-44 | ger | https://pubmed.ncbi.nlm.nih.gov/10081288/ | 10.1007/BF03044693 |
| TH/DZH | full text | full text not available | 1999 | Zharskiƒ≠ SL and Zharskaia IM | [Cardiovascular system in subjects who have previously suffered from hemorrhagic fever with renal syndrome]. | Terapevticheskii arkhiv | 71 | 11 | 23-Jun | rus | https://pubmed.ncbi.nlm.nih.gov/10626328/ |  |
| TH/DZH | full text | ultrasound results reported only for 3 patients | 2000 | Aker S and Ivens K and Pilaski J and Grabensee B and Heering P | [Acute renal failure in hantavirus infections]. | Medizinische Klinik (Munich, Germany : 1983) | 95 | 4 | 213-7 | ger | https://pubmed.ncbi.nlm.nih.gov/10808304/ | 10.1007/pl00002110 |
| TH/DZH | title/abstract | did not have primary data on US in VHF | 2000 | Gupta S and Singh SK and Taneja V and Goulatia RK and Bhagat A and Puliyel JM | Gall bladder wall edema in serology proven pediatric dengue hemorrhagic fever: a useful diagnostic finding which may help in prognostication. | Journal of tropical pediatrics | 46 | 3 | 179-81 | eng | https://pubmed.ncbi.nlm.nih.gov/10893923/ | 10.1093/tropej/46.3.179-a |
| TH/DZH | title/abstract | did not have primary data on US in VHF | 2000 | Khongphatthallayothin A and Chotivitayatarakorn P and Somchit S and Mitprasart A and Sakolsattayadorn S and Thisyakorn C | Morbitz type I second degree AV block during recovery from dengue hemorrhagic fever. | The Southeast Asian journal of tropical medicine and public health | 31 | 4 | 642-5 | eng | https://pubmed.ncbi.nlm.nih.gov/11414404/ |  |
| TH/DZH | title/abstract | did not have primary data on US in VHF | 2000 | Sopontammarak S and Pruekprasert P | Concomitant dengue hemorrhagic fever with Kawasaki disease. | The Southeast Asian journal of tropical medicine and public health | 31 | 1 | 190-2 | eng | https://pubmed.ncbi.nlm.nih.gov/11023092/ |  |
| TH/DZH | full text | only 5/40 dengue cases confirmed | 2000 | Thulkar S and Sharma S and Srivastava DN and Sharma SK and Berry M and Pandey RM | Sonographic findings in grade III dengue hemorrhagic fever in adults. | Journal of clinical ultrasound : JCU | 28 | 1 | 34-7 | eng | https://pubmed.ncbi.nlm.nih.gov/10602103/ | 10.1002/(sici)1097-0096(200001)28:1<34::aid-jcu5>3.0.co;2-d |
| TH/DZH | title/abstract | US in VHF, but case study or series | 2000 | Van Troys H and Gras C and Coton T and Deparis X and Tolou H and Durand JP | [Imported dengue hemorrhagic fever: aprops of 1 case presenting with signs of acute alithiasic cholecystitis]. | Medecine tropicale : revue du Corps de sante colonial | 60 | 3 | 278-80 | fre | https://pubmed.ncbi.nlm.nih.gov/11258064/ |  |
| TH/DZH | title/abstract | did not have primary data on US in VHF | 2001 | Bolzan HE and Spatola J and Gonz√°lez J and Luna R and Garc√≠a G | [Precut Vater's papilla. Prospective evaluation of frequency of use, effectiveness, complication and mortality. Cooperative study in the northwest of the province of Buenos Aires]. | Acta gastroenterologica Latinoamericana | 31 | 4 | 323-7 | spa | https://pubmed.ncbi.nlm.nih.gov/11766544/ |  |
| TH/DZH | title/abstract | did not have primary data on US in VHF | 2001 | Cam BV and Fonsmark L and Hue NB and Phuong NT and Poulsen A and Heegaard ED | Prospective case-control study of encephalopathy in children with dengue hemorrhagic fever. | The American journal of tropical medicine and hygiene | 65 | 6 | 848-51 | eng | https://pubmed.ncbi.nlm.nih.gov/11791985/ | 10.4269/ajtmh.2001.65.848 |
| TH/DZH | full text | full text not available, from title/abstract appears no patient level ulrasound data | 2001 | del Valle D√≠az S and Pi√±era Mart√≠nez M and Guasch Saent-F√©lix F | [Reactive hepatitis caused by the hemorrhagic dengue virus]. | Revista cubana de medicina tropical | 53 | 1 | 28-31 | spa | https://pubmed.ncbi.nlm.nih.gov/11826534/ |  |
| TH/DZH | full text | INCLUDED: original patient level data on US in VHF | 2001 | Kim YO and Chun KA and Choi JY and Yoon SA and Yang CW and Kim KT and Bang BK | Sonographic evaluation of gallbladder-wall thickening in hemorrhagic fever with renal syndrome: prediction of disease severity. | Journal of clinical ultrasound : JCU | 29 | 5 | 286-9 | eng | https://pubmed.ncbi.nlm.nih.gov/11486323/ | 10.1002/jcu.1035 |
| TH/DZH | full text | INCLUDED: original patient level data on US in VHF | 2002 | Paakkala A and Kallio T and Huhtala H and Apuli P and Paakkala T and Pasternack A and Mustonen J | Renal ultrasound findings and their clinical associations in nephropathia epidemica. Analysis of quantitative parameters. | Acta radiologica (Stockholm, Sweden : 1987) | 43 | 3 | 320-5 | eng | https://pubmed.ncbi.nlm.nih.gov/12100331/ | 10.1080/j.1600-0455.2002.430315.x |
| TH/DZH | full text | full text not available | 2002 | Pimenov LT and Dudarev MV and Eshmakov SV | [Clinicofunctional characteristics of the heart in hemorrhagic fever with renal syndrome]. | Klinicheskaia meditsina | 80 | 10 | 28-31 | rus | https://pubmed.ncbi.nlm.nih.gov/12471834/ |  |
| TH/DZH | title/abstract | did not have primary data on US in VHF | 2002 | Rapp C and Debord T and Imbert P and Lambotte O and Rou√© R | [Splenic rupture in infectious disease: splenectomy or conservative treatment? Report of three cases]. | La Revue de medecine interne | 23 | 1 | 85-91 | fre | https://pubmed.ncbi.nlm.nih.gov/11859699/ | 10.1016/s0248-8663(01)00518-5 |
| TH/DZH | title/abstract | US in VHF, but case study or series | 2002 | Rueda E and M√©ndez A and Gonz√°lez G | [Hemophagocytic syndrome associated with dengue hemorrhagic fever]. | Biomedica : revista del Instituto Nacional de Salud | 22 | 2 | 160-6 | spa | https://pubmed.ncbi.nlm.nih.gov/12152482/ |  |
| TH/DZH | title/abstract | did not have primary data on US in VHF | 2002 | Sehgal A and Gupta S and Tyagi V and Bahl S and Singh SK and Puliyel JM | Gall bladder wall edema is not pathogenic of dengue infection. | Journal of tropical pediatrics | 48 | 5 | 315-6 | eng | https://pubmed.ncbi.nlm.nih.gov/12405179/ | 10.1093/tropej/48.5.315 |
| TH/DZH | title/abstract | did not have primary data on US in VHF | 2003 | Cruz-Villegas V and Berrocal AM and Davis JL | Bilateral choroidal effusions associated with dengue fever. | Retina (Philadelphia, Pa.) | 23 | 4 | 576-8 | eng | https://pubmed.ncbi.nlm.nih.gov/12972783/ | 10.1097/00006982-200308000-00031 |
| TH/DZH | title/abstract | did not have primary data on US in VHF | 2003 | Just-N√ºbling G and Korn S and Ludwig B and Stephan C and Doerr HW and Preiser W | Primary cytomegalovirus infection in an outpatient setting--laboratory markers and clinical aspects. | Infection | 31 | 5 | 318-23 | eng | https://pubmed.ncbi.nlm.nih.gov/14556056/ | 10.1007/s15010-003-3129-y |
| TH/DZH | full text | no patient level ultrasound data | 2003 | Khongphatthanayothin A and Suesaowalak M and Muangmingsook S and Bhattarakosol P and Pancharoen C | Hemodynamic profiles of patients with dengue hemorrhagic fever during toxic stage: an echocardiographic study. | Intensive care medicine | 29 | 4 | 570-4 | eng | https://pubmed.ncbi.nlm.nih.gov/12595978/ | 10.1007/s00134-003-1671-9 |
| TH/DZH | title/abstract | did not have primary data on US in VHF | 2003 | Rohani P and Green CJ and Mantilla-Beniers NB and Grenfell BT | Ecological interference between fatal diseases. | Nature | 422 | 6934 | 885-8 | eng | https://pubmed.ncbi.nlm.nih.gov/12712203/ | 10.1038/nature01542 |
| TH/DZH | full text | full text not available | 2003 | Tulumoviƒá D and Hukiƒá M and Mesiƒá E and Saboviƒá S and Tulumoviƒá A | [Ultrasound findings on abdominal organs in the acute phase of hemorrhagic fever with renal syndrome]. | Acta medica Croatica : casopis Hravatske akademije medicinskih znanosti | 57 | 5 | 427-31 | hrv | https://pubmed.ncbi.nlm.nih.gov/15011472/ |  |
| TH/DZH | title/abstract | US in VHF, but case study or series | 2004 | Kamantseva SM and Nafeeva EN and Nafeev AA | [Diagnosis of hemorrhagic fever with renal syndrome and bacterial endocarditis]. | Meditsinskaia parazitologiia i parazitarnye bolezni |  | 2 | 47-9 | rus | https://pubmed.ncbi.nlm.nih.gov/15193054/ |  |
| TH/DZH | full text | ultrasound results only for four patients and renal only | 2004 | Matthaeus T and Fries J and Weber M and Schulze-Lohoff E | [Glomerular-type proteinuria in hantavirus nephritis]. | Medizinische Klinik (Munich, Germany : 1983) | 99 | 5 | 223-7 | ger | https://pubmed.ncbi.nlm.nih.gov/15146285/ | 10.1007/s00063-004-1034-5 |
| TH/DZH | full text | same data as Paakala 2002 already included | 2004 | Paakkala A and Kallio T and Huhtala H and Apuli P and Paakkala T and Mustonen J | Value of ultrasonography in acute renal failure: analysis of qualitative features in patients with nephropathia epidemica. | Acta radiologica (Stockholm, Sweden : 1987) | 45 | 7 | 785-90 | eng | https://pubmed.ncbi.nlm.nih.gov/15624524/ | 10.1080/02841850410001295 |
| TH/DZH | full text | INCLUDED: original patient level data on US in VHF | 2004 | Wu KL and Changchien CS and Kuo CH and Chiu KW and Lu SN and Kuo CM and Chiu YC and Chou YP and Chuah SK | Early abdominal sonographic findings in patients with dengue fever. | Journal of clinical ultrasound : JCU | 32 | 8 | 386-8 | eng | https://pubmed.ncbi.nlm.nih.gov/15372445/ | 10.1002/jcu.20060 |
| TH/DZH | full text | ultrasound only completed in selected patients | 2004 | Zulkarnain I | Gallbladder edema in Dengue hemorrhagic fever and its association with haematocrit levels and type of infections. | Acta medica Indonesiana | 36 | 2 | 84-6 | eng | https://pubmed.ncbi.nlm.nih.gov/15931700/ |  |
| TH/DZH | title/abstract | did not have primary data on US in VHF | 2005 | Beeching N | Fever in the returning traveller. | Medicine (Abingdon, England : UK ed.) | 33 | 7 | 03-Jun | eng | https://pubmed.ncbi.nlm.nih.gov/32308535/ | 10.1383/medc.2005.33.7.3 |
| TH/DZH | title/abstract | did not have primary data on US in VHF | 2005 | Bolz√°n H and Spatola J and Chiarenza C | [Endoscopic resection of esophageal leiomyoma with elastic band ligation]. | Acta gastroenterologica Latinoamericana | 35 | 3 | 165-8 | spa | https://pubmed.ncbi.nlm.nih.gov/16333975/ |  |
| TH/DZH | full text | ultrasound only completed in selected patients | 2005 | Gonz√°lez D and Castro OE and Kour√≠ G and Perez J and Martinez E and Vazquez S and Rosario D and Cancio R and Guzman MG | Classical dengue hemorrhagic fever resulting from two dengue infections spaced 20 years or more apart: Havana, Dengue 3 epidemic, 2001-2002. | International journal of infectious diseases : IJID : official publication of the International Society for Infectious Diseases | 9 | 5 | 280-5 | eng | https://pubmed.ncbi.nlm.nih.gov/16023878/ | 10.1016/j.ijid.2004.07.012 |
| TH/DZH | title/abstract | did not have primary data on US in VHF | 2005 | Hanta I and Ucar G and Kuleci S and Ozbek S and Kocabas A | Superior vena cava syndrome: a rare clinical manifestation of Behcet's disease. | Clinical rheumatology | 24 | 3 | 314-5 | eng | https://pubmed.ncbi.nlm.nih.gov/15349796/ | 10.1007/s10067-004-0958-8 |
| TH/DZH | full text | full text not available | 2005 | Hukiƒá M and Tulumoviƒá D and Calkiƒá L | [The renal failure and capillary leak during the acute stage of (Dobrava) DOB and PUU (Puumala) infection]. | Medicinski arhiv | 59 | 4 | 227-30 | bos | https://pubmed.ncbi.nlm.nih.gov/16018388/ |  |
| TH/DZH | full text | no patient level ultrasound data | 2005 | Khongphatthanayothin A and Lertsapcharoen P and Supachokchaiwattana P and Satupan P and Thongchaiprasit K and Poovorawan Y and Thisyakorn C | Hepatosplanchnic circulatory dysfunction in acute hepatic infection: the case of dengue hemorrhagic fever. | Shock (Augusta, Ga.) | 24 | 5 | 407-11 | eng | https://pubmed.ncbi.nlm.nih.gov/16247324/ | 10.1097/01.shk.0000180981.70870.8c |
| TH/DZH | title/abstract | did not have primary data on US in VHF | 2005 | Mekmullica J and Pancharoen C and Deerojanawong J and Lertsapcharoen P and Thisyakorn U and Thisyakorn C | Concomitant dengue infection and Kawasaki disease in an infant: a case report and literature review. | Journal of the Medical Association of Thailand = Chotmaihet thangphaet | 88 | 3 | 436-9 | eng | https://pubmed.ncbi.nlm.nih.gov/15962658/ |  |
| TH/DZH | full text | no patient level ultrasound data | 2005 | Paakkala A and Dastidar P and Ryymin P and Huhtala H and Mustonen J | Renal MRI findings and their clinical associations in nephropathia epidemica: analysis of quantitative findings. | European radiology | 15 | 5 | 968-74 | eng | https://pubmed.ncbi.nlm.nih.gov/15185121/ | 10.1007/s00330-004-2363-8 |
| TH/DZH | title/abstract | did not have primary data on US in VHF | 2005 | Saxena AK | Dengue in travelers. | The New England journal of medicine | 353 | 23 | 2511-3; author reply 2511-3 | eng | https://pubmed.ncbi.nlm.nih.gov/16353300/ |  |
| TH/DZH | full text | INCLUDED: original patient level data on US in VHF | 2005 | Venkata Sai PM and Dev B and Krishnan R | Role of ultrasound in dengue fever. | The British journal of radiology | 78 | 929 | 416-8 | eng | https://pubmed.ncbi.nlm.nih.gov/15845934/ | 10.1259/bjr/54704044 |
| TH/DZH | full text | INCLUDED: original patient level data on US in VHF | 2006 | Balasubramanian S and Janakiraman L and Kumar SS and Muralinath S and Shivbalan S | A reappraisal of the criteria to diagnose plasma leakage in dengue hemorrhagic fever. | Indian pediatrics | 43 | 4 | 334-9 | eng | https://pubmed.ncbi.nlm.nih.gov/16651672/ |  |
| TH/DZH | title/abstract | did not have primary data on US in VHF | 2006 | Chieregato A and Sabia G and Tanfani A and Compagnone C and Tagliaferri F and Targa L | Xenon-CT and transcranial Doppler in poor-grade or complicated aneurysmatic subarachnoid hemorrhage patients undergoing aggressive management of intracranial hypertension. | Intensive care medicine | 32 | 8 | 1143-50 | eng | https://pubmed.ncbi.nlm.nih.gov/16783552/ | 10.1007/s00134-006-0226-2 |
| TH/DZH | full text | full text not available | 2006 | Lee MS and Hwang KP and Chen TC and Lu PL and Chen TP | Clinical characteristics of dengue and dengue hemorrhagic fever in a medical center of southern Taiwan during the 2002 epidemic. | Journal of microbiology, immunology, and infection = Wei mian yu gan ran za zhi | 39 | 2 | 121-9 | eng | https://pubmed.ncbi.nlm.nih.gov/16604244/ |  |
| TH/DZH | title/abstract | did not have primary data on US in VHF | 2006 | Neo HY and Wong RC and Seto KY and Yip JW and Yang H and Ling LH | Noncompaction cardiomyopathy presenting with congestive heart failure during intercurrent dengue viral illness: importance of phenotypic recognition. | International journal of cardiology | 107 | 1 | 123-5 | eng | https://pubmed.ncbi.nlm.nih.gov/16337512/ | 10.1016/j.ijcard.2005.01.064 |
| TH/DZH | full text | INCLUDED: original patient level data on US in VHF | 2006 | Quiroz-Moreno R and M√©ndez GF and Ovando-Rivera KM | [Clinical utility of ultrasound in the identification of dengue hemorrhagic fever]. | Revista medica del Instituto Mexicano del Seguro Social | 44 | 3 | 243-8 | spa | https://pubmed.ncbi.nlm.nih.gov/16870119/ |  |
| TH/DZH | title/abstract | did not have primary data on US in VHF | 2006 | Screaton G and Mongkolsapaya J | T cell responses and dengue haemorrhagic fever. | Novartis Foundation symposium | 277 |  | 164-71; discussion 171-6, 251-3 | eng | https://pubmed.ncbi.nlm.nih.gov/17319161/ |  |
| TH/DZH | full text | no patient level ultrasound data, case series slected for acalculous cholecystitis | 2006 | Sharma N and Mahi S and Bhalla A and Singh V and Varma S and Ratho RK | Dengue fever related acalculous cholecystitis in a North Indian tertiary care hospital. | Journal of gastroenterology and hepatology | 21 | 4 | 664-7 | eng | https://pubmed.ncbi.nlm.nih.gov/16677150/ | 10.1111/j.1440-1746.2006.04295.x |
| TH/DZH | title/abstract | did not have primary data on US in VHF | 2006 | Suzano CE and Amaral E and Sato HK and Papaiordanou PM | The effects of yellow fever immunization (17DD) inadvertently used in early pregnancy during a mass campaign in Brazil. | Vaccine | 24 | 9 | 1421-6 | eng | https://pubmed.ncbi.nlm.nih.gov/16236402/ | 10.1016/j.vaccine.2005.09.033 |
| TH/DZH | title/abstract | did not have primary data on US in VHF | 2007 | Bhatia V and Parida AK and Arora P and Mittal A and Pandey AK and Singh G and Vaishnava GC and Kaul U | Electrocardiographic and echocardiographic findings during the recent outbreak of viral fever in National Capital Region. | Indian heart journal | 59 | 4 | 360-2 | eng | https://pubmed.ncbi.nlm.nih.gov/19126944/ |  |
| TH/DZH | full text | INCLUDED: original patient level data on US in VHF | 2007 | Brito CA and Albuquerque Mde F and Lucena-Silva N | [Plasma leakage detection in severe dengue: when serum albumin quantification plays a role?]. | Revista da Sociedade Brasileira de Medicina Tropical | 40 | 2 | 220-3 | por | https://pubmed.ncbi.nlm.nih.gov/17568893/ | 10.1590/s0037-86822007000200015 |
| TH/DZH | full text | no patient level ultrasound data | 2007 | Colbert JA and Gordon A and Roxelin R and Silva S and Silva J and Rocha C and Harris E | Ultrasound measurement of gallbladder wall thickening as a diagnostic test and prognostic indicator for severe dengue in pediatric patients. | The Pediatric infectious disease journal | 26 | 9 | 850-2 | eng | https://pubmed.ncbi.nlm.nih.gov/17721386/ | 10.1097/INF.0b013e3180619692 |
| TH/DZH | title/abstract | US in VHF, but case study or series | 2007 | Jaufeerally FR and Surrun SK and Chang PE | Acute acalculous cholecystitis in dengue hemorrhagic fever. | Indian journal of medical sciences | 61 | 11 | 613-4 | eng | https://pubmed.ncbi.nlm.nih.gov/18025749/ |  |
| TH/DZH | title/abstract | US in VHF, but case study or series | 2007 | Joshipura VP and Soni HN and Patel NR and Haribhakti SP | Dengue fever presenting as acute acalculus cholecystitis. | Journal of the Indian Medical Association | 105 | 6 | 338-9 | eng | https://pubmed.ncbi.nlm.nih.gov/18232180/ |  |
| TH/DZH | full text | case series of patients with concern for appendicitis | 2007 | Premaratna R and Bailey MS and Ratnasena BG and de Silva HJ | Dengue fever mimicking acute appendicitis. | Transactions of the Royal Society of Tropical Medicine and Hygiene | 101 | 7 | 683-5 | eng | https://pubmed.ncbi.nlm.nih.gov/17368695/ | 10.1016/j.trstmh.2007.02.006 |
| TH/DZH | title/abstract | did not have primary data on US in VHF | 2007 | Rao HY and Wei L and Zhao JM and Sun DG | Thirteen to twenty-seven-year follow-up of patients with hepatitis C while transfusing or donating plasma. | Hepato-gastroenterology | 54 | 75 | 826-9 | eng | https://pubmed.ncbi.nlm.nih.gov/17591072/ |  |
| TH/DZH | title/abstract | US in VHF, but case study or series | 2007 | Saikia N and Talukdar R and Singal DK and Chaudhary D and Bhullar SS and Tandon RK | Hepatic calcification following dengue virus-induced fulminant hepatic failure. | Indian journal of gastroenterology : official journal of the Indian Society of Gastroenterology | 26 | 2 | 90-2 | eng | https://pubmed.ncbi.nlm.nih.gov/17558076/ |  |
| TH/DZH | full text | INCLUDED: original patient level data on US in VHF | 2007 | Srikiatkhachorn A and Krautrachue A and Ratanaprakarn W and Wongtapradit L and Nithipanya N and Kalayanarooj S and Nisalak A and Thomas SJ and Gibbons RV and Mammen MP Jr and Libraty DH and Ennis FA and Rothman AL and Green S | Natural history of plasma leakage in dengue hemorrhagic fever: a serial ultrasonographic study. | The Pediatric infectious disease journal | 26 | 4 | 283-90; discussion 291-2 | eng | https://pubmed.ncbi.nlm.nih.gov/17414388/ | 10.1097/01.inf.0000258612.26743.10 |
| TH/DZH | title/abstract | did not have primary data on US in VHF | 2008 | Chieregato A and Battaglia R and Sabia G and Compagnone C and Cocciolo F and Tagliaferri F and Pascarella R and Pasquini U and Frattarelli M and Targa L | A diagnostic flowchart, including TCD, Xe-CT and angiography, to improve the diagnosis of vasospasm critically affecting cerebral blood flow in patients with subarachnoid haemorrhage, sedated and ventilated. | Acta neurochirurgica. Supplement | 104 |  | 251-3 | eng | https://pubmed.ncbi.nlm.nih.gov/19382372/ | 10.1007/978-3-211-75718-5_50 |
| TH/DZH | full text | full text not available | 2008 | Dudarev MV and Pimenov LT | [Long-term outcomes and formation of chronic disease of the kidneys in patients with a history of hemorrhagic fever with renal syndrome]. | Terapevticheskii arkhiv | 80 | 6 | 59-62 | rus | https://pubmed.ncbi.nlm.nih.gov/18655478/ |  |
| TH/DZH | title/abstract | US in VHF, but case study or series | 2008 | Freitas GP | [Abdominal pain and thickening of the gallbladder wall]. | Acta gastroenterologica Latinoamericana | 38 | 3 | 172, 231 | spa | https://pubmed.ncbi.nlm.nih.gov/18979893/ |  |
| TH/DZH | title/abstract | US in VHF, but case study or series | 2008 | Georges CG and Artunc F and Weyrich P and Friedrich B and Wolf SC | [Nephropathia epidemica as the result of a Puumala virus infection in a pregnant patient]. | Deutsche medizinische Wochenschrift (1946) | 133 | 37 | 1830-2 | ger | https://pubmed.ncbi.nlm.nih.gov/18770481/ | 10.1055/s-0028-1082803 |
| TH/DZH | title/abstract | US in VHF, but case study or series | 2008 | Karunatilake H and Vithiya K and Arasalingam A and Malavan R and Kumara DS | Acalculous cholecystitis and dengue fever. | The Ceylon medical journal | 53 | 1 | 30 | eng | https://pubmed.ncbi.nlm.nih.gov/18590271/ |  |
| TH/DZH | title/abstract | did not have primary data on US in VHF | 2008 | Moretta G and Locatelli AJ and Gadola L and De Arteaga J and Sol√° L and Caporale N and Schargorodsky J and Ducasse E and Pastorino G and Marchetta N and Espeche W and Ortiz Z | Rio de La Plata study: a multicenter, cross-sectional study on cardiovascular risk factors and heart failure prevalence in peritoneal dialysis patients in Argentina and Uruguay. | Kidney international. Supplement |  | 108 | S159-64 | eng | https://pubmed.ncbi.nlm.nih.gov/18379540/ | 10.1038/sj.ki.5002618 |
| TH/DZH | full text | no true patients included | 2008 | Statler J and Mammen M and Lyons A and Sun W | Sonographic findings of healthy volunteers infected with dengue virus. | Journal of clinical ultrasound : JCU | 36 | 7 | 413-7 | eng | https://pubmed.ncbi.nlm.nih.gov/18446859/ | 10.1002/jcu.20476 |
| TH/DZH | full text | ultrasound only completed in selected patients | 2009 | Bhatty S and Shaikh NA and Fatima M and Sumbhuani AK | Acute acalculous cholecystitis in dengue fever. | JPMA. The Journal of the Pakistan Medical Association | 59 | 8 | 519-21 | eng | https://pubmed.ncbi.nlm.nih.gov/19757695/ |  |
| TH/DZH | full text | INCLUDED: original patient level data on US in VHF | 2009 | Engin A and Yilmaz MB and Elaldi N and Erdem A and Yalta K and Tandogan I and Kaya S and Bakir M and Dokmetas I | Crimean-Congo hemorrhagic fever: does it involve the heart? | International journal of infectious diseases : IJID : official publication of the International Society for Infectious Diseases | 13 | 3 | 369-73 | eng | https://pubmed.ncbi.nlm.nih.gov/18980852/ | 10.1016/j.ijid.2008.07.019 |
| TH/DZH | title/abstract | did not have primary data on US in VHF | 2009 | Hoffman L and Pierce D and Puumala S | Clinical predictors of injuries not identified by focused abdominal sonogram for trauma (FAST) examinations. | The Journal of emergency medicine | 36 | 3 | 271-9 | eng | https://pubmed.ncbi.nlm.nih.gov/18375089/ | 10.1016/j.jemermed.2007.09.035 |
| TH/DZH | title/abstract | did not have primary data on US in VHF | 2009 | Jin HY and Kang SM and Kim SY and Park JH and Baek HS and Park TS | A case of Graves' disease combined with hantaan virus infection. | Journal of Korean medical science | 24 | 1 | 158-61 | eng | https://pubmed.ncbi.nlm.nih.gov/19270831/ | 10.3346/jkms.2009.24.1.158 |
| TH/DZH | title/abstract | US in VHF, but case study or series | 2009 | Mahmood NS and Suresh HB and D'Souza S | Splenic necrosis as a rare complication of dengue fever. | Journal of clinical ultrasound : JCU | 37 | 9 | 527 | eng | https://pubmed.ncbi.nlm.nih.gov/19757423/ | 10.1002/jcu.20627 |
| TH/DZH | full text | INCLUDED: original patient level data on US in VHF | 2009 | Makela S and Kokkonen L and Ala-Houhala I and Groundstroem K and Harmoinen A and Huhtala H and Hurme M and Paakkala A and Porsti I and Virtanen V and Vaheri A and Mustonen J | More than half of the patients with acute Puumala hantavirus infection have abnormal cardiac findings. | Scandinavian journal of infectious diseases | 41 | 1 | 57-62 | eng | https://pubmed.ncbi.nlm.nih.gov/18932105/ | 10.1080/00365540802502629 |
| TH/DZH | title/abstract | did not have primary data on US in VHF | 2009 | Matsumura Y and Shimizu T | [Case of imported scrub typhus contracted in Myanmar]. | Kansenshogaku zasshi. The Journal of the Japanese Association for Infectious Diseases | 83 | 3 | 256-60 | jpn | https://pubmed.ncbi.nlm.nih.gov/19522310/ | 10.11150/kansenshogakuzasshi.83.256 |
| TH/DZH | title/abstract | US in VHF, but case study or series | 2009 | Nasim A | Dengue fever presenting as acute acalculous cholecystitis. | Journal of the College of Physicians and Surgeons--Pakistan : JCPSP | 19 | 8 | 531-3 | eng | https://pubmed.ncbi.nlm.nih.gov/19651022/ |  |
| TH/DZH | full text | ultrasound only completed in selected patients (with myocarditis) | 2009 | Salgado DM and Panqueba CA and Castro D and R Vega M and Rodr√≠guez JA | [Myocarditis in children affected by dengue hemorrhagic fever in a teaching hospital in Colombia]. | Revista de salud publica (Bogota, Colombia) | 11 | 4 | 591-600 | spa | https://pubmed.ncbi.nlm.nih.gov/20169215/ | 10.1590/s0124-00642009000400010 |
| TH/DZH | title/abstract | did not have primary data on US in VHF | 2010 | Handa T and Nagai S and Ueda S and Chin K and Ito Y and Watanabe K and Tanizawa K and Tamaya M and Mishima M and Izumi T | Significance of plasma NT-proBNP levels as a biomarker in the assessment of cardiac involvement and pulmonary hypertension in patients with sarcoidosis. | Sarcoidosis, vasculitis, and diffuse lung diseases : official journal of WASOG | 27 | 1 | 27-35 | eng | https://pubmed.ncbi.nlm.nih.gov/21086902/ |  |
| TH/DZH | full text | full text not available, from title/abstract appears ultrasound completed only in selected patients | 2010 | Jhamb R and Kumar A and Ranga GS and Rathi N | Unusual manifestations in dengue outbreak 2009, Delhi, India. | The Journal of communicable diseases | 42 | 4 | 255-61 | eng | https://pubmed.ncbi.nlm.nih.gov/22471194/ |  |
| TH/DZH | title/abstract | did not have primary data on US in VHF | 2010 | Misra UK and Kalita J and Phadke RV and Wadwekar V and Boruah DK and Srivastava A and Maurya PK and Bhattacharyya A | Usefulness of various MRI sequences in the diagnosis of viral encephalitis. | Acta tropica | 116 | 3 | 206-11 | eng | https://pubmed.ncbi.nlm.nih.gov/20816658/ | 10.1016/j.actatropica.2010.08.007 |
| TH/DZH | full text | INCLUDED: original patient level data on US in VHF | 2010 | Oliveira GA and Machado RC and Horvat JV and Gomes LE and Guerra LR and Vandesteen L and Oliveira FT and Lousada NS and Moreira-Silva S and de Fatima Ceolin M | Transient reticular gallbladder wall thickening in severe dengue fever: a reliable sign of plasma leakage. | Pediatric radiology | 40 | 5 | 720-4 | eng | https://pubmed.ncbi.nlm.nih.gov/20012951/ | 10.1007/s00247-009-1489-x |
| TH/DZH | title/abstract | did not have primary data on US in VHF | 2010 | Potts JA and Thomas SJ and Srikiatkhachorn A and Supradish PO and Li W and Nisalak A and Nimmannitya S and Endy TP and Libraty DH and Gibbons RV and Green S and Rothman AL and Kalayanarooj S | Classification of dengue illness based on readily available laboratory data. | The American journal of tropical medicine and hygiene | 83 | 4 | 781-8 | eng | https://pubmed.ncbi.nlm.nih.gov/20889865/ | 10.4269/ajtmh.2010.10-0135 |
| TH/DZH | full text | no patient level ultrasound data | 2010 | Salgado DM and Eltit JM and Mansfield K and Panqueba C and Castro D and Vega MR and Xhaja K and Schmidt D and Martin KJ and Allen PD and Rodriguez JA and Dinsmore JH and L√≥pez JR and Bosch I | Heart and skeletal muscle are targets of dengue virus infection. | The Pediatric infectious disease journal | 29 | 3 | 238-42 | eng | https://pubmed.ncbi.nlm.nih.gov/20032806/ | 10.1097/INF.0b013e3181bc3c5b |
| TH/DZH | full text | no patient level ultrasound data | 2010 | Shamim M | Frequency, pattern and management of acute abdomen in dengue fever in Karachi, Pakistan. | Asian journal of surgery | 33 | 3 | 107-13 | eng | https://pubmed.ncbi.nlm.nih.gov/21163407/ | 10.1016/S1015-9584(10)60019-X |
| TH/DZH | full text | ultrasound only completed in selected patients (imaging mostly CXR based) | 2011 | Ejaz K and Khursheed M and Raza A | Pleural effusion in dengue. Karachi perspective. | Saudi medical journal | 32 | 1 | 46-9 | eng | https://pubmed.ncbi.nlm.nih.gov/21212916/ |  |
| TH/DZH | full text | INCLUDED: original patient level data on US in VHF | 2011 | Gul I and Kaya A and G√ºven AS and Karapinar H and K√º√ß√ºkdurmaz Z and Yilmaz A and I√ßaƒüasioƒülu FD and Tandoƒüan I | Cardiac findings in children with Crimean-Congo hemorrhagic fever. | Medical science monitor : international medical journal of experimental and clinical research | 17 | 8 | CR457-460 | eng | https://pubmed.ncbi.nlm.nih.gov/21804465/ | 10.12659/MSM.881907 |
| TH/DZH | title/abstract | did not have primary data on US in VHF | 2011 | Kalayanarooj S | Dengue classification: current WHO vs. the newly suggested classification for better clinical application? | Journal of the Medical Association of Thailand = Chotmaihet thangphaet | 94 |  | S74-84 | eng | https://pubmed.ncbi.nlm.nih.gov/22043757/ |  |
| TH/DZH | title/abstract | US in VHF, but case study or series | 2011 | Marasinghe JP and Sriyasinghe RY and Wijewantha VI and Gunaratne KA and Wijeyaratne CN | Acute acalculous cholecystitis due to dengue hemorrhagic fever during pregnancy. | The journal of obstetrics and gynaecology research | 37 | 10 | 1489-92 | eng | https://pubmed.ncbi.nlm.nih.gov/21564414/ | 10.1111/j.1447-0756.2011.01537.x |
| TH/DZH | full text | INCLUDED: original patient level data on US in VHF | 2011 | Motla M and Manaktala S and Gupta V and Aggarwal M and Bhoi SK and Aggarwal P and Goel A | Sonographic evidence of ascites, pleura-pericardial effusion and gallbladder wall edema for dengue fever. | Prehospital and disaster medicine | 26 | 5 | 335-41 | eng | https://pubmed.ncbi.nlm.nih.gov/22030127/ | 10.1017/S1049023X11006637 |
| TH/DZH | title/abstract | US in VHF, but case study or series | 2011 | Tayeb B and Piot C and Roubille F | Acute pericarditis after dengue fever. | Annales de cardiologie et d'angeiologie | 60 | 4 | 240-2 | eng | https://pubmed.ncbi.nlm.nih.gov/21664601/ | 10.1016/j.ancard.2011.05.008 |
| TH/DZH | title/abstract | did not have primary data on US in VHF | 2011 | Valotto CF and Silva HH and Cavasin G and Geris R and Rodrigues Filho E and da Silva IG | [Ultrastructural alterations in larvae of Aedes aegypti subject to labdane diterpene isolated from Copaifera reticulata (Leguminosae) and a fraction enriched with tannins of Magonia pubescens (Sapindaceae)]. | Revista da Sociedade Brasileira de Medicina Tropical | 44 | 2 | 194-200 | por | https://pubmed.ncbi.nlm.nih.gov/21468474/ | 10.1590/s0037-86822011005000010 |
| TH/DZH | title/abstract | did not have primary data on US in VHF | 2011 | Wiwanitkit V | Sonographic evidence in dengue. | Prehospital and disaster medicine | 26 | 6 | 427 | eng | https://pubmed.ncbi.nlm.nih.gov/22433457/ | 10.1017/S1049023X11006753 |
| TH/DZH | title/abstract | did not have primary data on US in VHF | 2011 | Zaki SA | Pleural effusion and ultrasonography in dengue Fever. | Indian journal of community medicine : official publication of Indian Association of Preventive & Social Medicine | 36 | 2 | 163 | eng | https://pubmed.ncbi.nlm.nih.gov/21976806/ | 10.4103/0970-0218.84140 |
| TH/DZH | title/abstract | did not have primary data on US in VHF | 2011 | Zimmermann A and Lorenz B and Schmidt W | [Bilateral acute angle-closure glaucoma due to an infection with Hantavirus]. | Der Ophthalmologe : Zeitschrift der Deutschen Ophthalmologischen Gesellschaft | 108 | 8 | 753-8 | ger | https://pubmed.ncbi.nlm.nih.gov/21327947/ | 10.1007/s00347-010-2311-8 |
| TH/DZH | title/abstract | did not have primary data on US in VHF | 2012 | Almi≈ü H and Yakƒ±ncƒ± C | [A case of brucellosis misdiagnosed as Crimean-Congo hemorrhagic fever]. | Mikrobiyoloji bulteni | 46 | 3 | 475-9 | tur | https://pubmed.ncbi.nlm.nih.gov/22951660/ |  |
| TH/DZH | full text | INCLUDED: original patient level data on US in VHF | 2012 | Jagadishkumar K and Jain P and Manjunath VG and Umesh L | Hepatic involvement in dengue Fever in children. | Iranian journal of pediatrics | 22 | 2 | 231-6 | eng | https://pubmed.ncbi.nlm.nih.gov/23056891/ |  |
| TH/DZH | full text | no patient level ultrasound data | 2012 | Khositseth A and Tangnararatchakit K and Chuansumrit A and Wanitkun S and Kuptanon T and Chaiyaratana W and Yoksan S | Cardiovascular change in children with dengue shock syndrome. | Journal of pediatric intensive care | 1 | 3 | 153-160 | eng | https://pubmed.ncbi.nlm.nih.gov/31214401/ | 10.3233/PIC-2012-025 |
| TH/DZH | full text | INCLUDED: original patient level data on US in VHF | 2012 | Meltzer E and Heyman Z and Bin H and Schwartz E | Capillary leakage in travelers with dengue infection: implications for pathogenesis. | The American journal of tropical medicine and hygiene | 86 | 3 | 536-9 | eng | https://pubmed.ncbi.nlm.nih.gov/22403332/ | 10.4269/ajtmh.2012.10-0670 |
| TH/DZH | title/abstract | did not have primary data on US in VHF | 2012 | Seppelt IM and Orde SR | Why guess when you can see? Heart function and fluid management in dengue shock. | Critical care medicine | 40 | 2 | 675-6 | eng | https://pubmed.ncbi.nlm.nih.gov/22249046/ | 10.1097/CCM.0b013e31823d77e9 |
| TH/DZH | title/abstract | did not have primary data on US in VHF | 2012 | Taraphdar D and Sarkar A and Mukhopadhyay BB and Chakrabarti S and Chatterjee S | Rapid spread of chikungunya virus following its resurgence during 2006 in West Bengal, India. | Transactions of the Royal Society of Tropical Medicine and Hygiene | 106 | 3 | 160-6 | eng | https://pubmed.ncbi.nlm.nih.gov/22265077/ | 10.1016/j.trstmh.2011.10.016 |
| TH/DZH | full text | no patient level ultrasound data | 2012 | Yacoub S and Griffiths A and Chau TT and Simmons CP and Wills B and Hien TT and Henein M and Farrar J | Cardiac function in Vietnamese patients with different dengue severity grades. | Critical care medicine | 40 | 2 | 477-83 | eng | https://pubmed.ncbi.nlm.nih.gov/21946658/ | 10.1097/CCM.0b013e318232d966 |
| TH/DZH | title/abstract | did not have primary data on US in VHF | 2013 | Barr KL and Messenger AM and Anderson BD and Friary JA and Heil GL and Reece K and Gray GC | Recovery of live virus after storage at ambient temperature using ViveST‚Ñ¢. | Journal of clinical virology : the official publication of the Pan American Society for Clinical Virology | 56 | 1 | 57-61 | eng | https://pubmed.ncbi.nlm.nih.gov/23046621/ | 10.1016/j.jcv.2012.09.005 |
| TH/DZH | full text | INCLUDED: original patient level data on US in VHF | 2013 | Bharath Kumar Reddy KR and Laksmana RR and Veerappa BG and Shivananda | Ultrasonography as a tool in predicting the severity of dengue fever in children--a useful aid in a developing country. | Pediatric radiology | 43 | 8 | 971-7 | eng | https://pubmed.ncbi.nlm.nih.gov/23455371/ | 10.1007/s00247-013-2642-0 |
| TH/DZH | title/abstract | US in VHF, but case study or series | 2013 | Chandrashekar NK and Krishnappa R and Reddy CS and Narayan A | Hemoperitoneum in dengue Fever with normal coagulation profile. | Journal of global infectious diseases | 5 | 1 | 29-30 | eng | https://pubmed.ncbi.nlm.nih.gov/23599615/ | 10.4103/0974-777X.107172 |
| TH/DZH | title/abstract | US in VHF, but case study or series | 2013 | Fr√∂hlich R and R√∂mmele U | [Acalculous cholecystitis in hantavirus infections]. | Deutsche medizinische Wochenschrift (1946) | 138 | 23 | 1255-8 | ger | https://pubmed.ncbi.nlm.nih.gov/23720184/ | 10.1055/s-0033-1343197 |
| TH/DZH | title/abstract | did not have primary data on US in VHF | 2013 | Ghatak T and Singh RK and Baronia AK | Spontaneous central vein thrombosis in a patient with activated protein C resistance and dengue infection: An association or causation? | Journal of anaesthesiology, clinical pharmacology | 29 | 4 | 547-9 | eng | https://pubmed.ncbi.nlm.nih.gov/24249996/ | 10.4103/0970-9185.119145 |
| TH/DZH | title/abstract | US in VHF, but case study or series | 2013 | Hong WX and Zhao H and Deng YQ and Jiang T and Yu XD and Song KY and Wang J and Tan XH and Yu M and Zhu SY and Qin ED and Tang XP and Zhang FC and Qin CF | Severe dengue due to secondary DENV-1 infection in Mainland China. | Journal of clinical virology : the official publication of the Pan American Society for Clinical Virology | 57 | 2 | 184-6 | eng | https://pubmed.ncbi.nlm.nih.gov/23490400/ | 10.1016/j.jcv.2013.02.012 |
| TH/DZH | title/abstract | US in VHF, but case study or series | 2013 | Khongphatthanayothin A and Mahayosnond A and Poovorawan Y | Possible cause of liver failure in patient with dengue shock syndrome. | Emerging infectious diseases | 19 | 7 | 1161-3 | eng | https://pubmed.ncbi.nlm.nih.gov/23763890/ | 10.3201/eid1907.121820 |
| TH/DZH | title/abstract | US in VHF, but case study or series | 2013 | Mac√© G and Feyeux C and Mollard N and Chantegret C and Audia S and Rebibou JM and Spagnolo G and Bour JB and Denoyel GA and Sagot P and Reynes JM | Severe Seoul hantavirus infection in a pregnant woman, France, October 2012. | Euro surveillance : bulletin Europeen sur les maladies transmissibles = European communicable disease bulletin | 18 | 17 | 20464 | eng | https://pubmed.ncbi.nlm.nih.gov/23647626/ |  |
| TH/DZH | title/abstract | US in VHF, but case study or series | 2013 | Mar√≠n J and Vilcarromero S and Forshey BM and Celis-Salinas JC and Ramal-Asayag C and Morrison AC and Laguna-Torres A and Casap√≠a M and Halsey ES | [Acute gastrointestinal involvement in dengue disease by serotype 4: a case report and literature review]. | Revista chilena de infectologia : organo oficial de la Sociedad Chilena de Infectologia | 30 | 5 | 541-7 | spa | https://pubmed.ncbi.nlm.nih.gov/24248170/ | 10.4067/S0716-10182013000500012 |
| TH/DZH | title/abstract | US in VHF, but case study or series | 2013 | McFarlane ME and Plummer JM and Leake PA and Powell L and Chand V and Chung S and Tulloch K | Dengue fever mimicking acute appendicitis: A case report. | International journal of surgery case reports | 4 | 11 | 1032-4 | eng | https://pubmed.ncbi.nlm.nih.gov/24096347/ | 10.1016/j.ijscr.2013.08.017 |
| TH/DZH | full text | INCLUDED: original patient level data on US in VHF | 2013 | Michels M and Sumardi U and de Mast Q and Jusuf H and Puspita M and Dewi IM and Sinarta S and Alisjahbana B and van der Ven AJ | The predictive diagnostic value of serial daily bedside ultrasonography for severe dengue in Indonesian adults. | PLoS neglected tropical diseases | 7 | 6 | e2277 | eng | https://pubmed.ncbi.nlm.nih.gov/23785539/ | 10.1371/journal.pntd.0002277 |
| TH/DZH | full text | ultrasound only completed in selected patients (those with elevated biokmarkers) | 2013 | Miranda CH and Borges Mde C and Matsuno AK and Vilar FC and Gali LG and Volpe GJ and Schmidt A and Pazin-Filho A and Silva FM and Castro-Jorge LA and Oliveira MF and Saggioro F and Martines RB and Fonseca BA | Evaluation of cardiac involvement during dengue viral infection. | Clinical infectious diseases : an official publication of the Infectious Diseases Society of America | 57 | 6 | 812-9 | eng | https://pubmed.ncbi.nlm.nih.gov/23784923/ | 10.1093/cid/cit403 |
| TH/DZH | title/abstract | did not have primary data on US in VHF | 2013 | Mohammadian E and Junin R and Rahmani O and Idris AK | Effects of sonication radiation on oil recovery by ultrasonic waves stimulated water-flooding. | Ultrasonics | 53 | 2 | 607-14 | eng | https://pubmed.ncbi.nlm.nih.gov/23137783/ | 10.1016/j.ultras.2012.10.006 |
| TH/DZH | title/abstract | US in VHF, but case study or series | 2013 | Patra S and Bhardwaj G and Manohar JS and Srinivasa KH and Kharge J and Manjunath CN | Acute myocardial infarction being the presentation of dengue myocarditis. | Journal of cardiovascular disease research | 4 | 2 | 159-61 | eng | https://pubmed.ncbi.nlm.nih.gov/24027378/ | 10.1016/j.jcdr.2013.03.001 |
| TH/DZH | title/abstract | did not have primary data on US in VHF | 2013 | Picard NA and Zanardi CA | Brain motion in patients with skull defects: B-mode ultrasound observations on respiration-induced movements. | Acta neurochirurgica | 155 | 11 | 2149-57 | eng | https://pubmed.ncbi.nlm.nih.gov/24009045/ | 10.1007/s00701-013-1838-2 |
| TH/DZH | full text | no patient level ultrasound data | 2013 | Rasmuson J and Lindqvist P and S√∂rensen K and Hedstr√∂m M and Blomberg A and Ahlm C | Cardiopulmonary involvement in Puumala hantavirus infection. | BMC infectious diseases | 13 |  | 501 | eng | https://pubmed.ncbi.nlm.nih.gov/24160911/ | 10.1186/1471-2334-13-501 |
| TH/DZH | full text | no patient level ultrasound data | 2013 | Sengupta SP and Nugurwar A and Jaju R and Khandheria BK | Left ventricular myocardial performance in patients with dengue hemorrhagic fever and thrombocytopenia as assessed by two-dimensional speckle tracking echocardiography. | Indian heart journal | 65 | 3 | 276-82 | eng | https://pubmed.ncbi.nlm.nih.gov/23809381/ | 10.1016/j.ihj.2013.04.017 |
| TH/DZH | full text | no patient level ultrasound data | 2013 | Yadav DK and Choudhary S and Gupta PK and Beniwal MK and Agarwal S and Shukla U and Dubey NK and Sankar J and Kumar P | The Tei index and asymptomatic myocarditis in children with severe dengue. | Pediatric cardiology | 34 | 6 | 1307-13 | eng | https://pubmed.ncbi.nlm.nih.gov/23397334/ | 10.1007/s00246-013-0639-y |
| TH/DZH | title/abstract | did not have primary data on US in VHF | 2013 | Zhang G and Cowled C and Shi Z and Huang Z and Bishop-Lilly KA and Fang X and Wynne JW and Xiong Z and Baker ML and Zhao W and Tachedjian M and Zhu Y and Zhou P and Jiang X and Ng J and Yang L and Wu L and Xiao J and Feng Y and Chen Y and Sun X and Zhang Y and Marsh GA and Crameri G and Broder CC and Frey KG and Wang LF and Wang J | Comparative analysis of bat genomes provides insight into the evolution of flight and immunity. | Science (New York, N.Y.) | 339 | 6118 | 456-60 | eng | https://pubmed.ncbi.nlm.nih.gov/23258410/ | 10.1126/science.1230835 |
| TH/DZH | title/abstract | US in VHF, but case study or series | 2014 | Bendwal S and Malviya K and Jatav OP and Malviya K | Cardiac tamponade presenting as early manifestation in dengue fever. | The Journal of the Association of Physicians of India | 62 | 3 | 257-9 | eng | https://pubmed.ncbi.nlm.nih.gov/25327070/ |  |
| TH/DZH | full text | no patient level ultrasound data | 2014 | Eze KC and Salami TA and Kpolugbo JU | Acute abdominal pain in patients with lassa fever: Radiological assessment and diagnostic challenges. | Nigerian medical journal : journal of the Nigeria Medical Association | 55 | 3 | 195-200 | eng | https://pubmed.ncbi.nlm.nih.gov/25013248/ | 10.4103/0300-1652.132037 |
| TH/DZH | title/abstract | did not have primary data on US in VHF | 2014 | Fan J and Wei W and Bai Z and Fan C and Li S and Liu Q and Yang K | A systematic review and meta-analysis of dengue risk with temperature change. | International journal of environmental research and public health | 12 | 1 | Jan-15 | eng | https://pubmed.ncbi.nlm.nih.gov/25546270/ | 10.3390/ijerph120100001 |
| TH/DZH | title/abstract | did not have primary data on US in VHF | 2014 | Fong EJ and Johnston AC and Notton T and Jung SY and Rose KA and Weinberger LS and Shusteff M | Acoustic focusing with engineered node locations for high-performance microfluidic particle separation. | The Analyst | 139 | 5 | 1192-200 | eng | https://pubmed.ncbi.nlm.nih.gov/24448925/ | 10.1039/c4an00034j |
| TH/DZH | title/abstract | did not have primary data on US in VHF | 2014 | Guadalajara-Boo JF and Ruiz-Esparza ME and Aranda Frausto A and Soto Abraham MV and Gaspar-Hern√°ndez J | Histologic and angiographic imaging of acute shock dengue myocarditis. | Revista espanola de cardiologia (English ed.) | 67 | 3 | 226-7 | eng | https://pubmed.ncbi.nlm.nih.gov/24774402/ | 10.1016/j.rec.2013.09.009 |
| TH/DZH | title/abstract | did not have primary data on US in VHF | 2014 | Hamidi H and Mohammadian E and Junin R and Rafati R and Manan M and Azdarpour A and Junid M | A technique for evaluating the oil/heavy-oil viscosity changes under ultrasound in a simulated porous medium. | Ultrasonics | 54 | 2 | 655-62 | eng | https://pubmed.ncbi.nlm.nih.gov/24075416/ | 10.1016/j.ultras.2013.09.006 |
| TH/DZH | title/abstract | did not have primary data on US in VHF | 2014 | Honigsbaum M | Ebola: epidemic echoes and the chronicle of a tragedy foretold. | Lancet (London, England) | 384 | 9956 | 1740-1 | eng | https://pubmed.ncbi.nlm.nih.gov/25473684/ | 10.1016/s0140-6736(14)62063-8 |
| TH/DZH | full text | same data as Michels 2013 already included | 2014 | Michels M and Alisjahbana B and De Groot PG and Indrati AR and Fijnheer R and Puspita M and Dewi IM and van de Wijer L and de Boer EM and Roest M and van der Ven AJ and de Mast Q | Platelet function alterations in dengue are associated with plasma leakage. | Thrombosis and haemostasis | 112 | 2 | 352-62 | eng | https://pubmed.ncbi.nlm.nih.gov/24695691/ | 10.1160/TH14-01-0056 |
| TH/DZH | title/abstract | did not have primary data on US in VHF | 2014 | Nau JY | [October 2014, distant echoes of the Ebola epidemic]. | Revue medicale suisse | 10 | 450 | 2170-1 | fre | https://pubmed.ncbi.nlm.nih.gov/25549381/ |  |
| TH/DZH | full text | ultrasound only completed in selected patients | 2014 | Santhosh VR and Patil PG and Srinath MG and Kumar A and Jain A and Archana M | Sonography in the diagnosis and assessment of dengue Fever. | Journal of clinical imaging science | 4 |  | 14 | eng | https://pubmed.ncbi.nlm.nih.gov/24744971/ | 10.4103/2156-7514.129260 |
| TH/DZH | full text | no primary data of ultrasound | 2014 | Soller B and Srikiatkachorn A and Zou F and Rothman AL and Yoon IK and Gibbons RV and Kalayanarooj S and Thomas SJ and Green S | Preliminary evaluation of near infrared spectroscopy as a method to detect plasma leakage in children with dengue hemorrhagic fever. | BMC infectious diseases | 14 |  | 396 | eng | https://pubmed.ncbi.nlm.nih.gov/25033831/ | 10.1186/1471-2334-14-396 |
| TH/DZH | full text | INCLUDED: original patient level data on US in VHF | 2014 | Tufan ZK and Yigit H and Kacar M and Bulut C and Canpolat G and Hatipoglu CA and Kinikli S and Kosar P and Demiroz AP | Sonographic findings in patients with Crimean-Congo hemorrhagic fever. | Journal of ultrasound in medicine : official journal of the American Institute of Ultrasound in Medicine | 33 | 11 | 1999-2003 | eng | https://pubmed.ncbi.nlm.nih.gov/25336488/ | 10.7863/ultra.33.11.1999 |
| TH/DZH | full text | ultrasound only completed in selected patients (105/811) | 2014 | van de Weg CA and Pannuti CS and van den Ham HJ and de Ara√∫jo ES and Boas LS and Felix AC and Carvalho KI and Levi JE and Romano CM and Centrone CC and Rodrigues CL and Luna E and van Gorp EC and Osterhaus AD and Kallas EG and Martina BE | Serum angiopoietin-2 and soluble VEGF receptor 2 are surrogate markers for plasma leakage in patients with acute dengue virus infection. | Journal of clinical virology : the official publication of the Pan American Society for Clinical Virology | 60 | 4 | 328-35 | eng | https://pubmed.ncbi.nlm.nih.gov/24928471/ | 10.1016/j.jcv.2014.05.001 |
| TH/DZH | title/abstract | did not have primary data on US in VHF | 2014 | Vera MI and Mero√±o T and Urrutia MA and Parisi C and Morosan Y and Rosmarin M and Schnitman M and Brites F and Grisendi S and Serrano MS and Luciani W and Serrano L and Zuk C and De Barrio G and Cejas C and Faingold MC and Brenta G | Differential profile of ultrasound findings associated with malignancy in mixed and solid thyroid nodules in an elderly female population. | Journal of thyroid research | 2014 |  | 761653 | eng | https://pubmed.ncbi.nlm.nih.gov/25050189/ | 10.1155/2014/761653 |
| TH/DZH | full text | INCLUDED: original patient level data on US in VHF | 2014 | Ziraman I and Celikbas A and Ergonul O and Degirmenci T and Uyanik SA and Koparal S and Dokuzoguz B | Crimean-Congo hemorrhagic fever: aid of abdominal ultrasonography in prediction of severity. | Vector borne and zoonotic diseases (Larchmont, N.Y.) | 14 | 11 | 817-20 | eng | https://pubmed.ncbi.nlm.nih.gov/25409273/ | 10.1089/vbz.2014.1662 |
| TH/DZH | title/abstract | did not have primary data on US in VHF | 2015 | Anticona Huaynate CF and Pajuelo Traveza√±o MJ and Correa M and Mayta Malpartida H and Oberhelman R and Murphy LL and Paz-Soldan VA | Diagnostics barriers and innovations in rural areas: insights from junior medical doctors on the frontlines of rural care in Peru. | BMC health services research | 15 |  | 454 | eng | https://pubmed.ncbi.nlm.nih.gov/26438342/ | 10.1186/s12913-015-1114-7 |
| TH/DZH | title/abstract | did not have primary data on US in VHF | 2015 | Arora S and Nathaniel SD and Paul JC and Hansdak SG | Acute liver failure in dengue haemorrhagic fever. | BMJ case reports | 2015 |  |  | eng | https://pubmed.ncbi.nlm.nih.gov/26009600/ | 10.1136/bcr-2015-209443 |
| TH/DZH | full text | INCLUDED: original patient level data on US in VHF | 2015 | Arshad K and Sheikh S and Naqvi SU and Sarwar I and Javaid S and Asghar M and Butt MA | FREQUENCY OF SPLENOMEGALY IN DENGUE FEVER IN CHILDREN. | Journal of Ayub Medical College, Abbottabad : JAMC | 27 | 2 | 356-9 | eng | https://pubmed.ncbi.nlm.nih.gov/26411115/ |  |
| TH/DZH | title/abstract | US in VHF, but case study or series | 2015 | Badve SV and Patil S and Rathod NM and Jumrani CK | Dengue Fever and Takotsubo Cardiomyopathy. | The Journal of the Association of Physicians of India | 63 | 7 | 67-70 | eng | https://pubmed.ncbi.nlm.nih.gov/26731834/ |  |
| TH/DZH | title/abstract | did not have primary data on US in VHF | 2015 | Cellarier GR and Bordes J and Karkowski L and Gagnon N and Billhot M and Cournac JM and Rousseau C and De Greslan T and Mac Nab C and Dubrous P and Duron S and Moroge S and Quentin B | Safety, feasibility, and interest of transthoracic echocardiography in a deployed French military Ebola virus disease treatment center in Guinea. | Intensive care medicine | 41 | 8 | 1491-2 | eng | https://pubmed.ncbi.nlm.nih.gov/25952824/ | 10.1007/s00134-015-3821-2 |
| TH/DZH | title/abstract | did not have primary data on US in VHF | 2015 | Cho IH and Chang JH and Choo EJ | Bilateral simultaneous angle-closure glaucoma associated with septic condition of Korean hemorrhagic fever (KHF). | Journal of glaucoma | 24 | 1 | 81-3 | eng | https://pubmed.ncbi.nlm.nih.gov/23429623/ | 10.1097/IJG.0b013e318287ac5d |
| TH/DZH | title/abstract | did not have primary data on US in VHF | 2015 | Danthanarayana N and Williams DT and Williams SH and Thevanesam V and Speers DJ and Fernando MS | Acute meningoencephalitis associated with echovirus 9 infection in Sri Lanka, 2009. | Journal of medical virology | 87 | 12 | 2033-9 | eng | https://pubmed.ncbi.nlm.nih.gov/25983131/ | 10.1002/jmv.24267 |
| TH/DZH | title/abstract | did not have primary data on US in VHF | 2015 | Ibrahim F and Thio TH and Faisal T and Neuman M | The application of biomedical engineering techniques to the diagnosis and management of tropical diseases: a review. | Sensors (Basel, Switzerland) | 15 | 3 | 6947-95 | eng | https://pubmed.ncbi.nlm.nih.gov/25806872/ | 10.3390/s150306947 |
| TH/DZH | title/abstract | did not have primary data on US in VHF | 2015 | Johnston AM and Lewis SE | Decontaminating Ebola-infected ultrasound probes. | Anaesthesia | 70 | 5 | 628-9 | eng | https://pubmed.ncbi.nlm.nih.gov/25866043/ | 10.1111/anae.13060 |
| TH/DZH | full text | no patient level ultrasound data | 2015 | Kirawittaya T and Yoon IK and Wichit S and Green S and Ennis FA and Gibbons RV and Thomas SJ and Rothman AL and Kalayanarooj S and Srikiatkhachorn A | Evaluation of Cardiac Involvement in Children with Dengue by Serial Echocardiographic Studies. | PLoS neglected tropical diseases | 9 | 7 | e0003943 | eng | https://pubmed.ncbi.nlm.nih.gov/26226658/ | 10.1371/journal.pntd.0003943 |
| TH/DZH | full text | no ultrasound findings reported, included only patient selected for elevated lipase | 2015 | Kitterer D and Artunc F and Segerer S and Alscher MD and Braun N and Latus J | Evaluation of lipase levels in patients with nephropathia epidemica--no evidence for acute pancreatitis. | BMC infectious diseases | 15 |  | 286 | eng | https://pubmed.ncbi.nlm.nih.gov/26204892/ | 10.1186/s12879-015-1031-8 |
| TH/DZH | full text | same data as Michels 2013 already included | 2015 | Michels M and Japtok L and Alisjahbana B and Wisaksana R and Sumardi U and Puspita M and Kleuser B and de Mast Q and van der Ven AJ | Decreased plasma levels of the endothelial protective sphingosine-1-phosphate are associated with dengue-induced plasma leakage. | The Journal of infection | 71 | 4 | 480-7 | eng | https://pubmed.ncbi.nlm.nih.gov/26183296/ | 10.1016/j.jinf.2015.06.014 |
| TH/DZH | title/abstract | did not have primary data on US in VHF | 2015 | Minkoff H and Ecker J | Physicians' obligations to patients infected with Ebola: echoes of acquired immune deficiency syndrome. | American journal of obstetrics and gynecology | 212 | 4 | 456.e1-4 | eng | https://pubmed.ncbi.nlm.nih.gov/25530596/ | 10.1016/j.ajog.2014.12.026 |
| TH/DZH | title/abstract | did not have primary data on US in VHF | 2015 | Moreno CC and Kraft CS and Vanairsdale S and Kandiah P and Klopman MA and Ribner BS and Tridandapani S | Performance of bedside diagnostic ultrasound in an Ebola isolation unit: the Emory University Hospital experience. | AJR. American journal of roentgenology | 204 | 6 | 1157-9 | eng | https://pubmed.ncbi.nlm.nih.gov/25730332/ | 10.2214/AJR.15.14344 |
| TH/DZH | title/abstract | US in VHF, but case study or series | 2015 | Nicolas JB | Acalculous cholecystitis associated with hemorrhagic fever with renal syndrome. | Acta clinica Belgica | 70 | 5 | 377-81 | eng | https://pubmed.ncbi.nlm.nih.gov/25982259/ | 10.1179/2295333715Y.0000000028 |
| TH/DZH | title/abstract | did not have primary data on US in VHF | 2015 | Opderbeck T | A reply. | Anaesthesia | 70 | 5 | 629 | eng | https://pubmed.ncbi.nlm.nih.gov/25866045/ | 10.1111/anae.13072 |
| TH/DZH | full text | no patient level ultrasound data | 2015 | Premaratna R and Dissanayake D and Silva FH and Dassanayake M and de Silva HJ | Secondary bacteraemia in adult patients with prolonged dengue fever. | The Ceylon medical journal | 60 | 1 | 10-Feb | eng | https://pubmed.ncbi.nlm.nih.gov/25804911/ | 10.4038/cmj.v60i1.7165 |
| TH/DZH | title/abstract | did not have primary data on US in VHF | 2015 | Sagar PL | Nurses leading the fight against Ebola virus disease. | Journal of transcultural nursing : official journal of the Transcultural Nursing Society | 26 | 3 | 322-6 | eng | https://pubmed.ncbi.nlm.nih.gov/25712149/ | 10.1177/1043659615574326 |
| TH/DZH | full text | carotid doppler only | 2015 | Salk I and Kaya A and Cetin G and Egilmez H and Haydar Atalar M and Cetin A | Doppler ultrasonographic evaluation of the carotid and vertebral arteries in children with Crimean-Congo hemorrhagic fever. | Minerva pediatrica | 67 | 4 | 299-305 | eng | https://pubmed.ncbi.nlm.nih.gov/25243496/ |  |
| TH/DZH | title/abstract | did not have primary data on US in VHF | 2015 | Shivanthan MC and Navinan MR and Constantine GR and Rajapakse S | Cardiac involvement in dengue infection. | Journal of infection in developing countries | 9 | 4 | 338-46 | eng | https://pubmed.ncbi.nlm.nih.gov/25881521/ | 10.3855/jidc.6200 |
| TH/DZH | title/abstract | US in VHF, but case study or series | 2015 | Tahir H and Daruwalla V and Hayat S | Myocarditis leading to severe dilated cardiomyopathy in a patient with dengue Fever. | Case reports in cardiology | 2015 |  | 319312 | eng | https://pubmed.ncbi.nlm.nih.gov/25802766/ | 10.1155/2015/319312 |
| TH/DZH | title/abstract | did not have primary data on US in VHF | 2015 | Tan MW and Sklar GE | Pentoxifylline-Induced Thrombocytopenia: A Case Report. | Journal of pharmacy practice | 28 | 6 | 572-6 | eng | https://pubmed.ncbi.nlm.nih.gov/26429719/ | 10.1177/0897190015608274 |
| TH/DZH | full text | INCLUDED: original patient level data on US in VHF | 2015 | Taylor WR and Fox A and Pham KT and Le HNM and Tran NTH and Tran GV and Nguyen BT and Nguyen MV and Nguyen LT and Yacoub S and Nguyen HTT and Nguyen HH and Nguyen HD and Wertheim H and Horby P and Farrar J and Trinh LTM and Nguyen KV | Dengue in adults admitted to a referral hospital in Hanoi, Vietnam. | The American journal of tropical medicine and hygiene | 92 | 6 | 1141-1149 | eng | https://pubmed.ncbi.nlm.nih.gov/25918201/ | 10.4269/ajtmh.14-0472 |
| TH/DZH | title/abstract | did not have primary data on US in VHF | 2015 | Wiwanitkit V and Tambo E and Ugwu EC and Ngogang JY and Zhou XN | Are surveillance response systems enough to effectively combat and contain the Ebola outbreak? | Infectious diseases of poverty | 4 | 1 | 7 | eng | https://pubmed.ncbi.nlm.nih.gov/25671127/ | 10.1186/2049-9957-4-7 |
| TH/DZH | title/abstract | US in VHF, but case study or series | 2015 | Wolf T and Kann G and Becker S and Stephan C and Brodt HR and de Leuw P and Gr√ºnewald T and Vogl T and Kempf VA and Keppler OT and Zacharowski K | Severe Ebola virus disease with vascular leakage and multiorgan failure: treatment of a patient in intensive care. | Lancet (London, England) | 385 | 9976 | 1428-35 | eng | https://pubmed.ncbi.nlm.nih.gov/25534190/ | 10.1016/S0140-6736(14)62384-9 |
| TH/DZH | full text | INCLUDED: original patient level data on US in VHF | 2016 | Arora M and Patil RS | Cardiac Manifestation in Dengue Fever. | The Journal of the Association of Physicians of India | 64 | 7 | 40-44 | eng | https://pubmed.ncbi.nlm.nih.gov/27759341/ |  |
| TH/DZH | full text | INCLUDED: original patient level data on US in VHF | 2016 | Bandyopadhyay D and Chattaraj S and Hajra A and Mukhopadhyay S and Ganesan V | A Study on Spectrum of Hepatobiliary Dysfunctions and Pattern of Liver Involvement in Dengue Infection. | Journal of clinical and diagnostic research : JCDR | 10 | 5 | OC21-6 | eng | https://pubmed.ncbi.nlm.nih.gov/27437266/ | 10.7860/JCDR/2016/16946.7784 |
| TH/DZH | title/abstract | did not have primary data on US in VHF | 2016 | Beckmann K and Steffen F and Ohlerth S and Kircher PR and Carrera I | THREE TESLA MAGNETIC RESONANCE IMAGING FINDINGS IN 12 CASES OF CANINE CENTRAL EUROPEAN TICK-BORNE MENINGOENCEPHALOMYELITIS. | Veterinary radiology & ultrasound : the official journal of the American College of Veterinary Radiology and the International Veterinary Radiology Association | 57 | 1 | 41-8 | eng | https://pubmed.ncbi.nlm.nih.gov/26466523/ | 10.1111/vru.12303 |
| TH/DZH | title/abstract | did not have primary data on US in VHF | 2016 | Calvet G and Aguiar RS and Melo ASO and Sampaio SA and de Filippis I and Fabri A and Araujo ESM and de Sequeira PC and de Mendon√ßa MCL and de Oliveira L and Tschoeke DA and Schrago CG and Thompson FL and Brasil P and Dos Santos FB and Nogueira RMR and Tanuri A and de Filippis AMB | Detection and sequencing of Zika virus from amniotic fluid of fetuses with microcephaly in Brazil: a case study. | The Lancet. Infectious diseases | 16 | 6 | 653-660 | eng | https://pubmed.ncbi.nlm.nih.gov/26897108/ | 10.1016/S1473-3099(16)00095-5 |
| TH/DZH | full text | ultrasound only completed in selected patients (85/400) | 2016 | Chandak S and Kumar A | Can Radiology Play a Role in Early Diagnosis of Dengue Fever? | North American journal of medical sciences | 8 | 2 | 100-5 | eng | https://pubmed.ncbi.nlm.nih.gov/27042608/ | 10.4103/1947-2714.177316 |
| TH/DZH | title/abstract | US in VHF, but case study or series | 2016 | Chou MT and Yu WL | Takotsubo cardiomyopathy in a patient with dengue fever. | Journal of the Formosan Medical Association = Taiwan yi zhi | 115 | 9 | 818-9 | eng | https://pubmed.ncbi.nlm.nih.gov/27444241/ | 10.1016/j.jfma.2016.06.010 |
| TH/DZH | title/abstract | did not have primary data on US in VHF | 2016 | Deng Y and Zeng L and Bao W and Xu P and Zhong G | [Experience of integrated traditional Chinese and Western medicine in first case of imported Zika virus disease in China]. | Zhonghua wei zhong bing ji jiu yi xue | 28 | 2 | 106-9 | chi | https://pubmed.ncbi.nlm.nih.gov/26911940/ | 10.3760/cma.j.issn.2095-4352.2016.02.005 |
| TH/DZH | full text | no patient level ultrasound data | 2016 | Fernando S and Wijewickrama A and Gomes L and Punchihewa CT and Madusanka SD and Dissanayake H and Jeewandara C and Peiris H and Ogg GS and Malavige GN | Patterns and causes of liver involvement in acute dengue infection. | BMC infectious diseases | 16 |  | 319 | eng | https://pubmed.ncbi.nlm.nih.gov/27391896/ | 10.1186/s12879-016-1656-2 |
| TH/DZH | title/abstract | did not have primary data on US in VHF | 2016 | Gupta K and Banerjee A and Saggar K and Ahluwalia A and Saggar K | A prospective study of magnetic resonance imaging patterns of central nervous system infections in pediatric age group and young adults and their clinico-biochemical correlation. | Journal of pediatric neurosciences | 11 | 1 | 46-51 | eng | https://pubmed.ncbi.nlm.nih.gov/27195033/ | 10.4103/1817-1745.181244 |
| TH/DZH | full text | INCLUDED: original patient level data on US in VHF | 2016 | Khurram M and Qayyum W and Umar M and Jawad M and Mumtaz S and Bushra Khaar HT | Ultrasonographic pattern of plasma leak in dengue haemorrhagic fever. | JPMA. The Journal of the Pakistan Medical Association | 66 | 3 | 260-4 | eng | https://pubmed.ncbi.nlm.nih.gov/26968273/ |  |
| TH/DZH | title/abstract | US in VHF, but case study or series | 2016 | Kuna A and Wroczy≈Ñska A and Gajewski M and Felczak-Korzybska I and Nahorski WL | A case of acalculous cholecystitis in the course of dengue fever in a traveller returned from Brazil. | International maritime health | 67 | 1 | 38-41 | eng | https://pubmed.ncbi.nlm.nih.gov/27029928/ | 10.5603/IMH.2016.0008 |
| TH/DZH | full text | no patient level ultrasound data | 2016 | Li Y and Hu Z and Huang Y and Li J and Hong W and Qin Z and Tong Y and Li J and Lv M and Li M and Zheng X and Hu J and Hua J and Zhang F and Xu DL | Characterization of the Myocarditis during the worst outbreak of dengue infection in China. | Medicine | 95 | 27 | e4051 | eng | https://pubmed.ncbi.nlm.nih.gov/27399087/ | 10.1097/MD.0000000000004051 |
| TH/DZH | title/abstract | did not have primary data on US in VHF | 2016 | Melo AS and Aguiar RS and Amorim MM and Arruda MB and Melo FO and Ribeiro ST and Batista AG and Ferreira T and Dos Santos MP and Sampaio VV and Moura SR and Rabello LP and Gonzaga CE and Malinger G and Ximenes R and de Oliveira-Szejnfeld PS and Tovar-Moll F and Chimelli L and Silveira PP and Delvechio R and Higa L and Campanati L and Nogueira RM and Filippis AM and Szejnfeld J and Voloch CM and Ferreira OC Jr and Brindeiro RM and Tanuri A | Congenital Zika Virus Infection: Beyond Neonatal Microcephaly. | JAMA neurology | 73 | 12 | 1407-1416 | eng | https://pubmed.ncbi.nlm.nih.gov/27695855/ | 10.1001/jamaneurol.2016.3720 |
| TH/DZH | title/abstract | did not have primary data on US in VHF | 2016 | Mykhaylichenko VY and Kubyshkin AV and Samarin SA and Fomochkina II and Anisimova LV | Experimental induction of reparative morphogenesis and adaptive reserves in the ischemic myocardium using multipotent mesenchymal bone marrow-derived stem cells. | Pathophysiology : the official journal of the International Society for Pathophysiology | 23 | 2 | 95-104 | eng | https://pubmed.ncbi.nlm.nih.gov/27102896/ | 10.1016/j.pathophys.2016.04.002 |
| TH/DZH | title/abstract | did not have primary data on US in VHF | 2016 | Oliveira Melo AS and Malinger G and Ximenes R and Szejnfeld PO and Alves Sampaio S and Bispo de Filippis AM | Zika virus intrauterine infection causes fetal brain abnormality and microcephaly: tip of the iceberg? | Ultrasound in obstetrics & gynecology : the official journal of the International Society of Ultrasound in Obstetrics and Gynecology | 47 | 1 | 06-Jul | eng | https://pubmed.ncbi.nlm.nih.gov/26731034/ | 10.1002/uog.15831 |
| TH/DZH | title/abstract | did not have primary data on US in VHF | 2016 | Ozmen C and Deniz A and Akilli RE and Deveci OS and Cagliyan CE and Aktas H and Celik Aƒ∞ and Akpinar AA and Disel NR and Balli HT and Hanta ƒ∞ and Demir M and Usal A and Kanadasi M | Ultrasound Accelerated Thrombolysis May Be an Effective and Safe Treatment Modality for Intermediate Risk/Submassive Pulmonary Embolism. | International heart journal | 57 | 1 | 91-5 | eng | https://pubmed.ncbi.nlm.nih.gov/26673444/ | 10.1536/ihj.15-271 |
| TH/DZH | full text | INCLUDED: original patient level data on US in VHF | 2016 | Pothapregada S and Kullu P and Kamalakannan B and Thulasingam M | Is Ultrasound a Useful Tool to Predict Severe Dengue Infection? | Indian journal of pediatrics | 83 | 6 | 500-4 | eng | https://pubmed.ncbi.nlm.nih.gov/26846603/ | 10.1007/s12098-015-2013-y |
| TH/DZH | title/abstract | US in VHF, but case study or series | 2016 | Rao SV and Jacob GG and Raju NA and Ancheri SA | Spontaneous arterial hemorrhage as a complication of dengue. | Indian journal of critical care medicine : peer-reviewed, official publication of Indian Society of Critical Care Medicine | 20 | 5 | 302-4 | eng | https://pubmed.ncbi.nlm.nih.gov/27275081/ | 10.4103/0972-5229.182201 |
| TH/DZH | title/abstract | did not have primary data on US in VHF | 2016 | Schuler-Faccini L and Ribeiro EM and Feitosa IM and Horovitz DD and Cavalcanti DP and Pessoa A and Doriqui MJ and Neri JI and Neto JM and Wanderley HY and Cernach M and El-Husny AS and Pone MV and Serao CL and Sanseverino MT | Possible Association Between Zika Virus Infection and Microcephaly - Brazil, 2015. | MMWR. Morbidity and mortality weekly report | 65 | 3 | 59-62 | eng | https://pubmed.ncbi.nlm.nih.gov/26820244/ | 10.15585/mmwr.mm6503e2 |
| TH/DZH | full text | ultrasound only completed in selected patients (18/100) | 2016 | Sheetal S and Jacob E | A Study on the Cardiac Manifestations of Dengue. | The Journal of the Association of Physicians of India | 64 | 5 | 30-34 | eng | https://pubmed.ncbi.nlm.nih.gov/27735146/ |  |
| TH/DZH | title/abstract | US in VHF, but case study or series | 2016 | Sudulagunta SR and Sodalagunta MB and Sepehrar M and Bangalore Raja SK and Nataraju AS and Kumbhat M and Sathyanarayana D and Gummadi S and Burra HK | Dengue shock syndrome. | Oxford medical case reports | 2016 | 11 | omw074 | eng | https://pubmed.ncbi.nlm.nih.gov/28031845/ | 10.1093/omcr/omw074 |
| TH/DZH | full text | no patient level ultrasound data | 2016 | Thanachartwet V and Wattanathum A and Sahassananda D and Wacharasint P and Chamnanchanunt S and Khine Kyaw E and Jittmittraphap A and Naksomphun M and Surabotsophon M and Desakorn V | Dynamic Measurement of Hemodynamic Parameters and Cardiac Preload in Adults with Dengue: A Prospective Observational Study. | PloS one | 11 | 5 | e0156135 | eng | https://pubmed.ncbi.nlm.nih.gov/27196051/ | 10.1371/journal.pone.0156135 |
| TH/DZH | title/abstract | did not have primary data on US in VHF | 2016 | Tom√°s CC and Oliveira E and Sousa D and Uba-Chupel M and Furtado G and Rocha C and Teixeira A and Ferreira P and Alves C and Gisin S and Catarino E and Carvalho N and Coucelo T and Bonfim L and Silva C and Franco D and Gonz√°lez JA and Jardim HG and Silva R and Baixinho CL and Presado M¬™H and Marques M¬™F and Cardoso ME and Cunha M and Mendes J and Xavier A and Galhardo A and Couto M and Frade JG and Nunes C and Mesquita JR and Nascimento MS and Gon√ßalves G and Castro C and M√°rtires A and Monteiro M¬™J and Rainho C and Caballero FP and Monago FM and Guerrero JT and Monago RM and Trigo AP and Gutierrez ML and Milan√©s GM and Reina MG and Villanueva AG and Pi√±ero AS and Aliseda IR and Ramirez FB and Ribeiro A and Quelhas A and Manso C and Caballero FP and Guerrero JT and Monago FM and Santos RB and Jimenez NR and Nu√±ez CG and Gomez IR and Fernandez M¬™JL and Marquez LA and Moreno AL and Huertas M¬™JT and Ramirez FB and Seabra D and Salvador M¬™C and Braga L and Parreira P and Salgueiro-Oliveira A and Arreguy-Sena C and Oliveira BF and Henriques M¬™A and Santos J and Lebre S and Marques A and Festas C and Rodrigues S and Ribeiro A and Lumini J and Figueiredo AG and Hernandez-Martinez FJ and Campi L and Quintana-Montesdeoca M¬™P and Jimenez-Diaz JF and Rodriguez-De-Vera BC and Parente A and Mata M¬™A and Pereira AM¬™ and Fernandes A and Br√°s M and Pinto M¬™R and Parreira P and Basto ML and Rei AC and M√≥nico LM and Sousa G and Morna C and Freitas O and Freitas G and Jardim A and Vasconcelos R and Horta LG and Rosa RS and Kranz LF and Nugem RC and Siqueira MS and Bordin R and Kniess R and Lacerda JT and Guedes J and Machado I and Almeida S and Zilh√£o A and Alves H and Ribeiro √ì and Amaral AP and Santos A and Monteiro J and Rocha M¬™C and Cruz R and Amaral AP and Louren√ßo M and Rocha M¬™C and Cruz R and Antunes S and Mendon√ßa V and Andrade I and Os√≥rio N and Valado A and Caseiro A and Gabriel A and Martins AC and Mendes F and Cabral L and Ferreira M and Gon√ßalves A and Luz TD and Luz L and Martins R and Morgado A and Vale-Dias ML and Porta-Nova R and Fleig TC and Reuter √âM and Froemming MB and Guerreiro SL and Carvalho LL and Guedelha D and Coelho P and Pereira A and Calha A and Cordeiro R and Gon√ßalves A and Certo A and Galv√£o A and Mata M¬™A and Welter A and Pereira E and Ribeiro S and Kretzer M and Jim√©nez-D√≠az JF and Jim√©nez-Rodr√≠guez C and Hern√°ndez-Mart√≠nez FJ and Rodr√≠guez-De-Vera BDC and Marques-Rodrigues A and Coelho P and Bernardes T and Pereira A and Sousa P and Filho JG and Nazario N and Kretzer M and Amaral O and Garrido A and Veiga N and Nunes C and Pedro AR and Pereira C and Almeia A and Fernandes HM and Vasconcelos C and Sousa N and Reis VM and Monteiro MJ and Mendes R and Pinto IC and Pires T and Gama J and Preto V and Silva N and Magalh√£es C and Martins M and Duarte M and Pa√∫l C and Mart√≠n I and Pinheiro AA and Xavier S and Azevedo J and Bento E and Marques C and Marques M and Macedo A and Pereira AT and Almeida JP and Almeida A and Alves J and Sousa N and Saavedra F and Mendes R and Maia AS | Proceedings of the 3rd IPLeiria's International Health Congress : Leiria, Portugal. 6-7 May 2016. | BMC health services research | 16 |  | 200 | eng | https://pubmed.ncbi.nlm.nih.gov/27409075/ | 10.1186/s12913-016-1423-5 |
| TH/DZH | title/abstract | did not have primary data on US in VHF | 2016 | Uhl JF and Lo Vuolo M and Labropoulos N | Anatomy of the lymph node venous networks of the groin and their investigation by ultrasonography. | Phlebology | 31 | 5 | 334-43 | eng | https://pubmed.ncbi.nlm.nih.gov/26060061/ | 10.1177/0268355515585039 |
| TH/DZH | title/abstract | did not have primary data on US in VHF | 2016 | Yacoub S and Lam PK and Vu le HM and Le TL and Ha NT and Toan TT and Van NT and Quyen NT and Le Duyen HT and Van Kinh N and Fox A and Mongkolspaya J and Wolbers M and Simmons CP and Screaton GR and Wertheim H and Wills B | Association of Microvascular Function and Endothelial Biomarkers With Clinical Outcome in Dengue: An Observational Study. | The Journal of infectious diseases | 214 | 5 | 697-706 | eng | https://pubmed.ncbi.nlm.nih.gov/27230099/ | 10.1093/infdis/jiw220 |
| TH/DZH | title/abstract | did not have primary data on US in VHF | 2017 | Alvarez MF and Bol√≠var-Mej√≠a A and Rodriguez-Morales AJ and Ramirez-Vallejo E | Cardiovascular involvement and manifestations of systemic Chikungunya virus infection: A systematic review. | F1000Research | 6 |  | 390 | eng | https://pubmed.ncbi.nlm.nih.gov/28503297/ | 10.12688/f1000research.11078.2 |
| TH/DZH | title/abstract | did not have primary data on US in VHF | 2017 | Bramley JC and Drummond CG and Lennemann NJ and Good CA and Kim KS and Coyne CB | A Three-Dimensional Cell Culture System To Model RNA Virus Infections at the Blood-Brain Barrier. | mSphere | 2 | 3 |  | eng | https://pubmed.ncbi.nlm.nih.gov/28656176/ | 10.1128/mSphere.00206-17 |
| TH/DZH | title/abstract | did not have primary data on US in VHF | 2017 | Cao Z and Liu T and Li X and Wang J and Lin H and Chen L and Wu Z and Ma W | Individual and Interactive Effects of Socio-Ecological Factors on Dengue Fever at Fine Spatial Scale: A Geographical Detector-Based Analysis. | International journal of environmental research and public health | 14 | 7 |  | eng | https://pubmed.ncbi.nlm.nih.gov/28714925/ | 10.3390/ijerph14070795 |
| TH/DZH | title/abstract | did not have primary data on US in VHF | 2017 | Delaine M and Weingertner AS and Nougairede A and Lepiller Q and Fafi-Kremer S and Favre R and Charrel R | Microcephaly Caused by Lymphocytic Choriomeningitis Virus. | Emerging infectious diseases | 23 | 9 | 1548-1550 | eng | https://pubmed.ncbi.nlm.nih.gov/28820372/ | 10.3201/eid2309.170775 |
| TH/DZH | title/abstract | did not have primary data on US in VHF | 2017 | Duarte G and Moron AF and Timerman A and Fernandes CE and Mariani Neto C and Almeida Filho GL and Werner Junior H and Esp√≠rito Santo HFBD and Steibel JAP and Bortoletti Filho J and Andrade JBB and Burl√° M and Silva de S√° MF and Busso NE and Giraldo PC and Moreira de S√° RA and Passini Junior R and Mattar R and Francisco RPV | Zika Virus Infection in Pregnant Women and Microcephaly. | Revista brasileira de ginecologia e obstetricia : revista da Federacao Brasileira das Sociedades de Ginecologia e Obstetricia | 39 | 5 | 235-248 | eng | https://pubmed.ncbi.nlm.nih.gov/28575919/ | 10.1055/s-0037-1603450 |
| TH/DZH | title/abstract | did not have primary data on US in VHF | 2017 | Eppes C and Rac M and Dunn J and Versalovic J and Murray KO and Suter MA and Sanz Cortes M and Espinoza J and Seferovic MD and Lee W and Hotez P and Mastrobattista J and Clark SL and Belfort MA and Aagaard KM | Testing for Zika virus infection in pregnancy: key concepts to deal with an emerging epidemic. | American journal of obstetrics and gynecology | 216 | 3 | 209-225 | eng | https://pubmed.ncbi.nlm.nih.gov/28126366/ | 10.1016/j.ajog.2017.01.020 |
| TH/DZH | title/abstract | US in VHF, but case study or series | 2017 | Fernandes AIV and Mendes CL and Sim√µes RH and Silva AEVF and Madruga CB and Brito CAA and Castellano LR and Cordeiro MT | Cardiac tamponade in a patient with severe dengue fever. | Revista da Sociedade Brasileira de Medicina Tropical | 50 | 5 | 701-705 | eng | https://pubmed.ncbi.nlm.nih.gov/29160522/ | 10.1590/0037-8682-0361-2016 |
| TH/DZH | title/abstract | did not have primary data on US in VHF | 2017 | Garg RK and Rizvi I and Ingole R and Jain A and Malhotra HS and Kumar N and Batra D | Cortical laminar necrosis in dengue encephalitis-a case report. | BMC neurology | 17 | 1 | 79 | eng | https://pubmed.ncbi.nlm.nih.gov/28427358/ | 10.1186/s12883-017-0855-9 |
| TH/DZH | title/abstract | did not have primary data on US in VHF | 2017 | Haussig JM and Severi E and Baum JH and Vanlerberghe V and Laiseca A and Defrance L and Brailescu C and Coulombier D and Jansa J | The European Medical Corps: first Public Health Team mission and future perspectives. | Euro surveillance : bulletin Europeen sur les maladies transmissibles = European communicable disease bulletin | 22 | 37 |  | eng | https://pubmed.ncbi.nlm.nih.gov/28933343/ | 10.2807/1560-7917.ES.2017.22.37.30613 |
| TH/DZH | title/abstract | did not have primary data on US in VHF | 2017 | Heard-Garris N and Arora S and Lurie N | Building Physician Networks as Part of the Zika Response. | Disaster medicine and public health preparedness | 11 | 2 | 259-261 | eng | https://pubmed.ncbi.nlm.nih.gov/28430098/ | 10.1017/dmp.2017.24 |
| TH/DZH | title/abstract | did not have primary data on US in VHF | 2017 | Joob B and Wiwanitkit V | Letter to Editor Commenting on "Patterns of Gall Bladder Wall Thickening in Dengue Fever". | Ultrasound international open | 3 | 4 | E165 | eng | https://pubmed.ncbi.nlm.nih.gov/29226276/ | 10.1055/s-0043-119418 |
| TH/DZH | title/abstract | did not have primary data on US in VHF | 2017 | Jugpal TS and Dixit R and Garg A and Gupta S and Jain V and Patel R and Agarwal S | Spectrum of findings on magnetic resonance imaging of the brain in patients with neurological manifestations of dengue fever. | Radiologia brasileira | 50 | 5 | 285-290 | eng | https://pubmed.ncbi.nlm.nih.gov/29085161/ | 10.1590/0100-3984.2016.0048 |
| TH/DZH | title/abstract | did not have primary data on US in VHF | 2017 | Korman AM and Alikhan A and Kaffenberger BH | Viral exanthems: An update on laboratory testing of the adult patient. | Journal of the American Academy of Dermatology | 76 | 3 | 538-550 | eng | https://pubmed.ncbi.nlm.nih.gov/28413059/ | 10.1016/j.jaad.2016.08.034 |
| TH/DZH | title/abstract | did not have primary data on US in VHF | 2017 | Levaggi ND and Lucas AN and Barletta J√ÅE | Bilateral acute angle closure in a patient with dengue fever: a case report. | Arquivos brasileiros de oftalmologia | 80 | 4 | 266-267 | eng | https://pubmed.ncbi.nlm.nih.gov/28954031/ | 10.5935/0004-2749.20170065 |
| TH/DZH | title/abstract | did not have primary data on US in VHF | 2017 | Lin HZ and Tambyah PA and Yong EL and Biswas A and Chan SY | A review of Zika virus infections in pregnancy and implications for antenatal care in Singapore. | Singapore medical journal | 58 | 4 | 171-178 | eng | https://pubmed.ncbi.nlm.nih.gov/28429036/ | 10.11622/smedj.2017026 |
| TH/DZH | title/abstract | did not have primary data on US in VHF | 2017 | McQuilkin PA and Niescierenko M and Beddoe AM and Goentzel J and Graham EA and Henwood PC and Rehwaldt L and Teklu S and Tupesis J and Marshall R | Academic Medical Support to the Ebola Virus Disease Outbreak in Liberia. | Academic medicine : journal of the Association of American Medical Colleges | 92 | 12 | 1674-1679 | eng | https://pubmed.ncbi.nlm.nih.gov/29019800/ | 10.1097/ACM.0000000000001959 |
| TH/DZH | title/abstract | US in VHF, but case study or series | 2017 | Osuna-Ramos JF and Silva-Gracia C and Maya-Vacio GJ and Romero-Utrilla A and R√≠os-Burgue√±o ER and Velarde-F√©lix JS | [Coexistence of acute appendicitis and dengue fever: A case report]. | Cirugia y cirujanos | 85 |  | 44-48 | spa | https://pubmed.ncbi.nlm.nih.gov/28062078/ | 10.1016/j.circir.2016.11.009 |
| TH/DZH | full text | same data as Parmar 2017 already included | 2017 | Parmar J and Mohan C and Kumar GP and Vora M | Ultrasound is Not Useful as a Screening Tool for Dengue Fever. | Polish journal of radiology | 82 |  | 693-700 | eng | https://pubmed.ncbi.nlm.nih.gov/29657635/ | 10.12659/PJR.902861 |
| TH/DZH | full text | INCLUDED: original patient level data on US in VHF | 2017 | Parmar JP and Mohan C and Vora M | Patterns of Gall Bladder Wall Thickening in Dengue Fever: A Mirror of the Severity of Disease. | Ultrasound international open | 3 | 2 | E76-E81 | eng | https://pubmed.ncbi.nlm.nih.gov/28603785/ | 10.1055/s-0043-105262 |
| TH/DZH | title/abstract | did not have primary data on US in VHF | 2017 | Pham LD and Phung NH and Le NT and Vo TQ | Economic report on the cost of dengue fever in Vietnam: case of a provincial hospital. | ClinicoEconomics and outcomes research : CEOR | 9 |  | 01-Aug | eng | https://pubmed.ncbi.nlm.nih.gov/28031723/ | 10.2147/CEOR.S124023 |
| TH/DZH | title/abstract | did not have primary data on US in VHF | 2017 | Premjibhai Parmar J, Mohan C, Vora M | Letter to Editor Commenting on "Patterns of Gall Bladder Wall Thickening in Dengue Fever" - Reply. | Ultrasound international open | 3 | 4 | E166 | eng | <https://pubmed.ncbi.nlm.nih.gov/29226277/> | 10.1055/s-0043-123174 |
| TH/DZH | full text | INCLUDED: original patient level data on US in VHF | 2017 | Ramabhatta S and Palaniappan S and Hanumantharayappa N and Begum SV | The Clinical and Serological Profile of Pediatric Dengue. | Indian journal of pediatrics | 84 | 12 | 897-901 | eng | https://pubmed.ncbi.nlm.nih.gov/28887788/ | 10.1007/s12098-017-2423-0 |
| TH/DZH | title/abstract | did not have primary data on US in VHF | 2017 | Roberts L | Echoes of Ebola as plague hits Madagascar. | Science (New York, N.Y.) | 358 | 6362 | 430-431 | eng | https://pubmed.ncbi.nlm.nih.gov/29074744/ | 10.1126/science.358.6362.430 |
| TH/DZH | title/abstract | did not have primary data on US in VHF | 2017 | Salerno A and Celentano AM and L√≥pez J and Lara V and Gaozza C and Balcazar DE and Carrillo C and Frank FM and Blanco MM | Novel 2-arylazoimidazole derivatives as inhibitors of Trypanosoma cruzi proliferation: Synthesis and evaluation of their biological activity. | European journal of medicinal chemistry | 125 |  | 327-334 | eng | https://pubmed.ncbi.nlm.nih.gov/27688187/ | 10.1016/j.ejmech.2016.09.045 |
| TH/DZH | title/abstract | did not have primary data on US in VHF | 2017 | Tanyel E and Sunbul M and Fletcher T and Leblebicioglu H | A case of endocarditis mimicking Crimean-Congo haemorrhagic fever. | Tropical doctor | 47 | 1 | 71-72 | eng | https://pubmed.ncbi.nlm.nih.gov/27486029/ | 10.1177/0049475516660465 |
| TH/DZH | title/abstract | US in VHF, but case study or series | 2017 | Teh YH and Tan YP and Zain MM | Dengue shock syndrome with retroperitoneal haematoma requiring repeated artery angioembolization. | The Medical journal of Malaysia | 72 | 4 | 254-256 | eng | https://pubmed.ncbi.nlm.nih.gov/28889142/ |  |
| TH/DZH | title/abstract | did not have primary data on US in VHF | 2017 | Varghese FS and Rausalu K and Hakanen M and Saul S and K√ºmmerer BM and Susi P and Merits A and Ahola T | Obatoclax Inhibits Alphavirus Membrane Fusion by Neutralizing the Acidic Environment of Endocytic Compartments. | Antimicrobial agents and chemotherapy | 61 | 3 |  | eng | https://pubmed.ncbi.nlm.nih.gov/27993855/ | 10.1128/AAC.02227-16 |
| TH/DZH | title/abstract | did not have primary data on US in VHF | 2017 | Wiwanitkit S and Wiwanitkit V | Dengue fever, takotsubo syndrome, cerebral edema, catecholamine monitoring, and serial echocardiography. | Journal of the Formosan Medical Association = Taiwan yi zhi | 116 | 4 | 329 | eng | https://pubmed.ncbi.nlm.nih.gov/28161073/ | 10.1016/j.jfma.2017.01.001 |
| TH/DZH | title/abstract | did not have primary data on US in VHF | 2017 | Wu Z and Hu J and Zeng T and Zhang ZL and Chen J and Wong G and Qiu X and Liu W and Gao GF and Bi Y and Pang DW | Ultrasensitive Ebola Virus Detection Based on Electroluminescent Nanospheres and Immunomagnetic Separation. | Analytical chemistry | 89 | 3 | 2039-2048 | eng | https://pubmed.ncbi.nlm.nih.gov/28208277/ | 10.1021/acs.analchem.6b04632 |
| TH/DZH | full text | no patient level ultrasound data | 2017 | Yacoub S and Trung TH and Lam PK and Thien VHN and Hai DHT and Phan TQ and Nguyet OPK and Quyen NTH and Simmons CP and Broyd C and Screaton GR and Wills B | Cardio-haemodynamic assessment and venous lactate in severe dengue: Relationship with recurrent shock and respiratory distress. | PLoS neglected tropical diseases | 11 | 7 | e0005740 | eng | https://pubmed.ncbi.nlm.nih.gov/28692675/ | 10.1371/journal.pntd.0005740 |
| TH/DZH | title/abstract | did not have primary data on US in VHF | 2017 | Zacharias N and Whitty J and Noblin S and Tsakiri S and Garcia J and Covinsky M and Bhattacharjee M and Saulino D and Tatevian N and Blackwell S | First Neonatal Demise with Travel-Associated Zika Virus Infection in the United States of America. | AJP reports | 7 | 2 | e68-e73 | eng | https://pubmed.ncbi.nlm.nih.gov/28413694/ | 10.1055/s-0037-1601890 |
| TH/DZH | title/abstract | did not have primary data on US in VHF | 2018 | Adams Waldorf KM and Nelson BR and Stencel-Baerenwald JE and Studholme C and Kapur RP and Armistead B and Walker CL and Merillat S and Vornhagen J and Tisoncik-Go J and Baldessari A and Coleman M and Dighe MK and Shaw DWW and Roby JA and Santana-Ufret V and Boldenow E and Li J and Gao X and Davis MA and Swanstrom JA and Jensen K and Widman DG and Baric RS and Medwid JT and Hanley KA and Ogle J and Gough GM and Lee W and English C and Durning WM and Thiel J and Gatenby C and Dewey EC and Fairgrieve MR and Hodge RD and Grant RF and Kuller L and Dobyns WB and Hevner RF and Gale M Jr and Rajagopal L | Congenital Zika virus infection as a silent pathology with loss of neurogenic output in the fetal brain. | Nature medicine | 24 | 3 | 368-374 | eng | https://pubmed.ncbi.nlm.nih.gov/29400709/ | 10.1038/nm.4485 |
| TH/DZH | title/abstract | US in VHF, but case study or series | 2018 | Anam AM and Rabbani R and Shumy F | Spontaneous calf haematoma in severe dengue. | BMJ case reports | 2018 |  |  | eng | https://pubmed.ncbi.nlm.nih.gov/29374645/ | 10.1136/bcr-2017-222932 |
| TH/DZH | title/abstract | did not have primary data on US in VHF | 2018 | Bawankar P and Lahane T and Parekh R and Lahane S and Lahane S and Pathak P and Sonawane S | An unusual occurrence of stromal keratitis in dengue fever. | Indian journal of ophthalmology | 66 | 11 | 1631-1633 | eng | https://pubmed.ncbi.nlm.nih.gov/30355887/ | 10.4103/ijo.IJO_387_18 |
| TH/DZH | title/abstract | did not have primary data on US in VHF | 2018 | Borrero Landazabal MA and Carre√±o Otero AL and Kouznetsov VV and Duque Luna JE and Mendez-Sanchez SC | Alterations of mitochondrial electron transport chain and oxidative stress induced by alkaloid-like Œ±-aminonitriles on Aedes aegypti larvae. | Pesticide biochemistry and physiology | 144 |  | 64-70 | eng | https://pubmed.ncbi.nlm.nih.gov/29463410/ | 10.1016/j.pestbp.2017.11.006 |
| TH/DZH | title/abstract | did not have primary data on US in VHF | 2018 | de Souza AS and de Oliveira-Szjenfeld PS and de Oliveira Melo AS and de Souza LAM and Batista AGM and Tovar-Moll F | Imaging findings in congenital Zika virus infection syndrome: an update. | Child's nervous system : ChNS : official journal of the International Society for Pediatric Neurosurgery | 34 | 1 | 85-93 | eng | https://pubmed.ncbi.nlm.nih.gov/29181810/ | 10.1007/s00381-017-3637-1 |
| TH/DZH | title/abstract | did not have primary data on US in VHF | 2018 | Echols B | "Heal Thyself."‚ÄîAn Argument for Granting Asylum to Healthcare Workers Persecuted During the 2014 West African Ebola Crisis. | SMU law review : a publication of Southern Methodist University School of Law | 71 | 4 | 1153-79 | eng | https://pubmed.ncbi.nlm.nih.gov/30648831/ |  |
| TH/DZH | title/abstract | US in VHF, but case study or series | 2018 | Ehelepola NDB and Rajapaksha RKGM and Dhanapala DMUB and Thennekoon TDK and Ponnamperuma S | Concurrent methicillin-resistant Staphylococcus aureus septicemia and pyomyositis in a patient with dengue hemorrhagic fever: a case report. | BMC infectious diseases | 18 | 1 | 99 | eng | https://pubmed.ncbi.nlm.nih.gov/29486726/ | 10.1186/s12879-018-3012-1 |
| TH/DZH | title/abstract | did not have primary data on US in VHF | 2018 | Giovanetti M and Goes de Jesus J and Lima de Maia M and Junior JX and Castro Amarante MF and Viana P and Khouri Barreto F and de Cerqueira EM and Pedreira Santos N and Barreto Falc√£o M and Machado Costa MC and Barros Fai√ßal AV and Andrade de Melo AL and Santos Amorim L and de Siqueira IC and Macarenhas DL and Bispo de Filippis AM and Rodrigues Faria N and Xavier Acosta A and J√∫nior Alc√¢ntara LC | Genetic evidence of Zika virus in mother's breast milk and body fluids of a newborn with severe congenital defects. | Clinical microbiology and infection : the official publication of the European Society of Clinical Microbiology and Infectious Diseases | 24 | 10 | 1111-1112 | eng | https://pubmed.ncbi.nlm.nih.gov/29906587/ | 10.1016/j.cmi.2018.06.008 |
| TH/DZH | title/abstract | did not have primary data on US in VHF | 2018 | Ianevski A and Zusinaite E and Kuivanen S and Strand M and Lysvand H and Teppor M and Kakkola L and Paavilainen H and Laajala M and Kallio-Kokko H and Valkonen M and Kantele A and Telling K and Lutsar I and Letjuka P and Metelitsa N and Oksenych V and Bj√∏r√•s M and Nordb√∏ SA and Dumpis U and Vitkauskiene A and √ñhrmalm C and Bondeson K and Bergqvist A and Aittokallio T and Cox RJ and Evander M and Hukkanen V and Marjomaki V and Julkunen I and Vapalahti O and Tenson T and Merits A and Kainov D | Novel activities of safe-in-human broad-spectrum antiviral agents. | Antiviral research | 154 |  | 174-182 | eng | https://pubmed.ncbi.nlm.nih.gov/29698664/ | 10.1016/j.antiviral.2018.04.016 |
| TH/DZH | title/abstract | did not have primary data on US in VHF | 2018 | Iannaccone A and Bruno G and Ravera A and Gay F and Salvini M and Bringhen S and Sabia L and Avenatti E and Veglio F and Milan A | Evaluation of Cardiovascular Toxicity Associated with Treatments Containing Proteasome Inhibitors in Multiple Myeloma Therapy. | High blood pressure & cardiovascular prevention : the official journal of the Italian Society of Hypertension | 25 | 2 | 209-218 | eng | https://pubmed.ncbi.nlm.nih.gov/29582365/ | 10.1007/s40292-018-0256-1 |
| TH/DZH | title/abstract | did not have primary data on US in VHF | 2018 | Jayaweera JAAS and Abeydeera WPH and Ranasinghe GR | Intravenously administered cloxacillin-induced neutropenia with eosinophilia in a patient with infective endocarditis: a case report. | Journal of medical case reports | 12 | 1 | 384 | eng | https://pubmed.ncbi.nlm.nih.gov/30593283/ | 10.1186/s13256-018-1933-3 |
| TH/DZH | title/abstract | did not have primary data on US in VHF | 2018 | Joob B and Wiwanitkit V | Ultrasound and screening tool for dengue fever. | Polish journal of radiology | 83 |  | e587 | eng | https://pubmed.ncbi.nlm.nih.gov/30800197/ | 10.5114/pjr.2018.81253 |
| TH/DZH | title/abstract | US in VHF, but case study or series | 2018 | Kularatne SAM and Rajapakse MM and Ralapanawa U and Waduge R and Pathirage LPMMK and Rajapakse RPVJ | Heart and liver are infected in fatal cases of dengue: three PCR based case studies. | BMC infectious diseases | 18 | 1 | 681 | eng | https://pubmed.ncbi.nlm.nih.gov/30567553/ | 10.1186/s12879-018-3603-x |
| TH/DZH | full text | no patient level ultrasound data | 2018 | Kularatne SAM and Ralapanawa U and Dalugama C and Jayasinghe J and Rupasinghe S and Kumarihamy P | Series of 10 dengue fever cases with unusual presentations and complications in Sri Lanka: a single centre experience in 2016. | BMC infectious diseases | 18 | 1 | 674 | eng | https://pubmed.ncbi.nlm.nih.gov/30563480/ | 10.1186/s12879-018-3596-5 |
| TH/DZH | full text | no patient level ultrasound data; TTE only looking at LV function | 2018 | Lakshman A and Balasubramanian P and Nampoothiri RV and Vijayvergiya R and Bhalla A and Varma SC | Elevated cardiac biomarkers and echocardiographic left ventricular dysfunction at admission in patients with dengue fever: report from a tertiary care center in Northwest India. | Tropical doctor | 48 | 4 | 261-265 | eng | https://pubmed.ncbi.nlm.nih.gov/29991327/ | 10.1177/0049475518785315 |
| TH/DZH | title/abstract | did not have primary data on US in VHF | 2018 | Low GK and Looi SY and Yong MH and Sharma D | Predictive and diagnostic test accuracy of ultrasonography in differentiating severe dengue from nonsevere dengue. | Journal of vector borne diseases | 55 | 2 | 79-88 | eng | https://pubmed.ncbi.nlm.nih.gov/30280705/ | 10.4103/0972-9062.242568 |
| TH/DZH | full text | INCLUDED: original patient level data on US in VHF | 2018 | Nainggolan L and Wiguna C and Hasan I and Dewiasty E | Gallbladder Wall Thickening for Early Detection of Plasma Leakage in Dengue Infected Adult Patients. | Acta medica Indonesiana | 50 | 3 | 193-199 | eng | https://pubmed.ncbi.nlm.nih.gov/30333268/ |  |
| TH/DZH | title/abstract | did not have primary data on US in VHF | 2018 | Nelwan EJ | Early Detection of Plasma Leakage in Dengue Hemorrhagic Fever. | Acta medica Indonesiana | 50 | 3 | 183-184 | eng | https://pubmed.ncbi.nlm.nih.gov/30333266/ |  |
| TH/DZH | title/abstract | did not have primary data on US in VHF | 2018 | Pereira AM and Monteiro DLM and Werner H and Daltro P and Fazecas T and Guedes B and Tonni G and Peixoto AB and Araujo J√∫nior E | Zika virus and pregnancy in Brazil: What happened? | Journal of the Turkish German Gynecological Association | 19 | 1 | 39-47 | eng | https://pubmed.ncbi.nlm.nih.gov/29503261/ | 10.4274/jtgga.2017.0072 |
| TH/DZH | full text | no patient level ultrasound data | 2018 | Rabbani MU and Aslam M and Zaheer MS and Ashraf MU | Clinical and Laboratory Profile of Dengue Fever in a North Indian Tertiary Hospital. | The Journal of the Association of Physicians of India | 66 | 4 | 37-9 | eng | https://pubmed.ncbi.nlm.nih.gov/30347950/ |  |
| TH/DZH | title/abstract | did not have primary data on US in VHF | 2018 | Sabia L and Avenatti E and Cesareo M and Leone D and Tosello F and Veglio F and Milan A | Evaluation of aortic stiffness by a new simplified 2D speckle tracking analysis. | The international journal of cardiovascular imaging | 34 | 11 | 1753-1760 | eng | https://pubmed.ncbi.nlm.nih.gov/29931554/ | 10.1007/s10554-018-1400-7 |
| TH/DZH | title/abstract | US in VHF, but case study or series | 2018 | Shah S and Rolfe R and Henostroza G and Seas C | Ultrasound Findings of Plasma Leakage in Dengue Fever. | The American journal of tropical medicine and hygiene | 99 | 6 | 1362-1363 | eng | https://pubmed.ncbi.nlm.nih.gov/30851024/ | 10.4269/ajtmh.18-0422 |
| TH/DZH | title/abstract | did not have primary data on US in VHF | 2018 | Walker CL and Merriam AA and Ohuma EO and Dighe MK and Gale M Jr and Rajagopal L and Papageorghiou AT and Gyamfi-Bannerman C and Adams Waldorf KM | Femur-sparing pattern of abnormal fetal growth in pregnant women from New York City after maternal Zika virus infection. | American journal of obstetrics and gynecology | 219 | 2 | 187.e1-187.e20 | eng | https://pubmed.ncbi.nlm.nih.gov/29738748/ | 10.1016/j.ajog.2018.04.047 |
| TH/DZH | title/abstract | did not have primary data on US in VHF | 2018 | Wiyatno A and Febrianti ESZ and Dewantari AK and Myint KS and Safari D and Idris NS | Characterization of rhinovirus C from a 4-year-old boy with acute onset dilated cardiomyopathy in Jakarta, Indonesia. | JMM case reports | 5 | 9 | e005139 | eng | https://pubmed.ncbi.nlm.nih.gov/30425833/ | 10.1099/jmmcr.0.005139 |
| TH/DZH | title/abstract | did not have primary data on US in VHF | 2019 | Andersen PI and Krpina K and Ianevski A and Shtaida N and Jo E and Yang J and Koit S and Tenson T and Hukkanen V and Anthonsen MW and Bjoras M and Evander M and Windisch MP and Zusinaite E and Kainov DE | Novel Antiviral Activities of Obatoclax, Emetine, Niclosamide, Brequinar, and Homoharringtonine. | Viruses | 11 | 10 |  | eng | https://pubmed.ncbi.nlm.nih.gov/31635418/ | 10.3390/v11100964 |
| TH/DZH | title/abstract | did not have primary data on US in VHF | 2019 | Barsosio HC and Gitonga JN and Karanja HK and Nyamwaya DK and Omuoyo DO and Kamau E and Hamaluba MM and Nyiro JU and Kitsao BS and Nyaguara A and Mwakio S and Newton CR and Sang R and Wright D and Sanders EJ and Seale AC and Agoti CN and Berkley JA and Bejon P and Warimwe GM | Congenital microcephaly unrelated to flavivirus exposure in coastal Kenya. | Wellcome open research | 4 |  | 179 | eng | https://pubmed.ncbi.nlm.nih.gov/32175480/ | 10.12688/wellcomeopenres.15568.1 |
| TH/DZH | title/abstract | US in VHF, but case study or series | 2019 | Biswas S and Kumar P and Tansir G and Biswas A | Case Report: Cardiac Tamponade in Dengue Hemorrhagic Fever: An Unusual Manifestation of a Common Disease. | The American journal of tropical medicine and hygiene | 101 | 2 | 448-450 | eng | https://pubmed.ncbi.nlm.nih.gov/31162011/ | 10.4269/ajtmh.19-0153 |
| TH/DZH | title/abstract | did not have primary data on US in VHF | 2019 | Candelo E and Caicedo G and Rosso F and Ballesteros A and Orrego J and Escobar L and Lapunzina P and Nevado J and Pachajoa H | First report case with negative genetic study (array CGH, exome sequencing) in patients with vertical transmission of Zika virus infection and associated brain abnormalities. | The application of clinical genetics | 12 |  | 141-150 | eng | https://pubmed.ncbi.nlm.nih.gov/31440073/ | 10.2147/TACG.S190661 |
| TH/DZH | full text | INCLUDED: original patient level data on US in VHF | 2019 | Datta G and Mitra P | A Study on Cardiac Manifestations of Dengue Fever. | The Journal of the Association of Physicians of India | 67 | 7 | 14-16 | eng | https://pubmed.ncbi.nlm.nih.gov/31559761/ |  |
| TH/DZH | title/abstract | did not have primary data on US in VHF | 2019 | Duarte-Neto AN and Monteiro RAA and Johnsson J and Cunha MDP and Pour SZ and Saraiva AC and Ho YL and da Silva LFF and Mauad T and Zanotto PMA and Saldiva PHN and de Oliveira IRS and Dolhnikoff M | Ultrasound-guided minimally invasive autopsy as a tool for rapid post-mortem diagnosis in the 2018 Sao Paulo yellow fever epidemic: Correlation with conventional autopsy. | PLoS neglected tropical diseases | 13 | 7 | e0007625 | eng | https://pubmed.ncbi.nlm.nih.gov/31329590/ | 10.1371/journal.pntd.0007625 |
| TH/DZH | title/abstract | did not have primary data on US in VHF | 2019 | Ehelepola NDB and Kumara GDNR and Sapurugala SACS and Buddhadasa WMNP and Dissanayake WP | An Atypical Case of Rickettsial Spotted Fever Myocarditis Mimicking Weil's Disease. | Case reports in infectious diseases | 2019 |  | 9620245 | eng | https://pubmed.ncbi.nlm.nih.gov/31360559/ | 10.1155/2019/9620245 |
| TH/DZH | title/abstract | US in VHF, but case study or series | 2019 | Farias LABG and Beserra FLCN and Fernandes L and Teixeira AAR and Ferragut JM and Gir√£o ES and Pires Neto RDJ | Myocarditis Following Recent Chikungunya and Dengue Virus Coinfection: A Case Report. | Arquivos brasileiros de cardiologia | 113 | 4 | 783-786 | ["eng", "por"] | https://pubmed.ncbi.nlm.nih.gov/31553384/ | 10.5935/abc.20190187 |
| TH/DZH | full text | INCLUDED: original patient level data on US in VHF | 2019 | Ferreira BDC and Correia D | Ultrasound Assessment of Hepatobiliary and Splenic Changes in Patients With Dengue and Warning Signs During the Acute and Recovery Phases. | Journal of ultrasound in medicine : official journal of the American Institute of Ultrasound in Medicine | 38 | 8 | 2015-2024 | eng | https://pubmed.ncbi.nlm.nih.gov/30549307/ | 10.1002/jum.14890 |
| TH/DZH | title/abstract | did not have primary data on US in VHF | 2019 | Grandez-Urbina JA and Corrales-Acosta E and Tejeda-Mariaca JE and Pichardo-Rodriguez R and Garcia-Perdomo H | Case Report: Penile necrosis associated to paraphimosis with calciphylaxis due to terminal chronic kidney disease. | F1000Research | 8 |  | 1133 | eng | https://pubmed.ncbi.nlm.nih.gov/31448110/ | 10.12688/f1000research.18834.1 |
| TH/DZH | title/abstract | did not have primary data on US in VHF | 2019 | Henwood PC | Imaging an Outbreak - Ultrasound in an Ebola Treatment Unit. | The New England journal of medicine | 381 | 1 | 06-Sep | eng | https://pubmed.ncbi.nlm.nih.gov/31269362/ | 10.1056/NEJMp1902830 |
| TH/DZH | title/abstract | did not have primary data on US in VHF | 2019 | Jardon M and Mohammad SF and Jude CM and Pahwa A | Imaging of Emerging Infectious Diseases. | Current radiology reports | 7 | 9 | 25 | eng | https://pubmed.ncbi.nlm.nih.gov/32226652/ | 10.1007/s40134-019-0338-4 |
| TH/DZH | title/abstract | did not have primary data on US in VHF | 2019 | Jayathilaka PGNS and Mendis ASV and Perera MHMTS and Damsiri HMT and Gunaratne AVC and Agampodi SB | An outbreak of leptospirosis with predominant cardiac involvement: a case series. | BMC infectious diseases | 19 | 1 | 265 | eng | https://pubmed.ncbi.nlm.nih.gov/30885170/ | 10.1186/s12879-019-3905-7 |
| TH/DZH | full text | INCLUDED: original patient level data on US in VHF | 2019 | Karava≈ü E and Karake√ßili F | Evaluation of Portal Vein Doppler Findings in Patients with Crimean‚ÅªCongo Hemorrhagic Fever. | Medicina (Kaunas, Lithuania) | 55 | 5 |  | eng | https://pubmed.ncbi.nlm.nih.gov/31100950/ | 10.3390/medicina55050146 |
| TH/DZH | title/abstract | US in VHF, but case study or series | 2019 | Krumm P and Zitzelsberger T and Gawaz M and Greulich S | Young patient with hantavirus-induced myocarditis detected by comprehensive cardiac magnetic resonance assessment. | BMC infectious diseases | 19 | 1 | 15 | eng | https://pubmed.ncbi.nlm.nih.gov/30612548/ | 10.1186/s12879-018-3658-8 |
| TH/DZH | title/abstract | US in VHF, but case study or series | 2019 | Matthias AT and Apsara S and Epa A | A case report of dengue haemorrhagic fever complicated with psoas haematoma requiring blood transfusion. | BMC infectious diseases | 19 | 1 | 385 | eng | https://pubmed.ncbi.nlm.nih.gov/31060498/ | 10.1186/s12879-019-4023-2 |
| TH/DZH | full text | INCLUDED: original patient level data on US in VHF | 2019 | Mohanty B and Sunder A and Pathak S | Clinicolaboratory profile of expanded dengue syndrome - Our experience in a teaching hospital. | Journal of family medicine and primary care | 8 | 3 | 1022-1027 | eng | https://pubmed.ncbi.nlm.nih.gov/31041245/ | 10.4103/jfmpc.jfmpc_12_19 |
| TH/DZH | full text | INCLUDED: original patient level data on US in VHF | 2019 | Paix√£o GMM and Nunes MCP and Beato BDVG and Sable C and Beaton AZ and Oliveira KKB and Rezende BDF and Rios JPP and Fraga CL and Pereira LS and Teixeira MRD and Oliveira NR and Pascoal-Xavier MA and Maciel GV and Brito CGX and J√∫nior MRL and Ribeiro ALP and Nascimento BR | Cardiac Involvement by Yellow Fever(from the PROVAR+ Study). | The American journal of cardiology | 123 | 5 | 833-838 | eng | https://pubmed.ncbi.nlm.nih.gov/30545483/ | 10.1016/j.amjcard.2018.11.032 |
| TH/DZH | title/abstract | did not have primary data on US in VHF | 2019 | Papali A and Adhikari NKJ and Diaz JV and Dondorp AM and D√ºnser MW and Jacob ST and Phua J and Romain M and Schultz MJ | Infrastructure and Organization of Adult Intensive Care Units in Resource-Limited Settings. |  |  |  | 31-68 | eng | https://pubmed.ncbi.nlm.nih.gov/32091695/ | 10.1007/978-3-030-03143-5_3 |
| TH/DZH | full text | INCLUDED: original patient level data on US in VHF | 2019 | Parmar J and Vora M and Mohan C and Shah S and Mahajan H and Patel T | "Honeycomb" pattern of gallbladder wall thickening - A forward step in early diagnosis of "Severe Dengue Fever". | The Indian journal of radiology & imaging | 29 | 1 | 14-18 | eng | https://pubmed.ncbi.nlm.nih.gov/31000936/ | 10.4103/ijri.IJRI_363_18 |
| TH/DZH | title/abstract | did not have primary data on US in VHF | 2019 | Roro M and Deressa W and Lindtj√∏rn B | Intrauterine growth patterns in rural Ethiopia compared with WHO and INTERGROWTH-21st growth standards: A community-based longitudinal study. | PloS one | 14 | 12 | e0226881 | eng | https://pubmed.ncbi.nlm.nih.gov/31891599/ | 10.1371/journal.pone.0226881 |
| TH/DZH | full text | no patient level ultrasound data | 2019 | Sigera PC and Amarasekara R and Rodrigo C and Rajapakse S and Weeratunga P and De Silva NL and Huang CH and Sahoo MK and Pinsky BA and Pillai DR and Tissera HA and Jayasinghe S and Handunnetti S and Fernando SD | Risk prediction for severe disease and better diagnostic accuracy in early dengue infection; the Colombo dengue study. | BMC infectious diseases | 19 | 1 | 680 | eng | https://pubmed.ncbi.nlm.nih.gov/31370795/ | 10.1186/s12879-019-4304-9 |
| TH/DZH | title/abstract | did not have primary data on US in VHF | 2019 | Tanizawa K and Handa T and Nagai S and Oga T and Kubo T and Ito Y and Aihara K and Ikezoe K and Sokai A and Nakatsuka Y and Hirai T and Chin K and Mishima M and Izumi T | Can the Sarcoidosis Health Questionnaire predict the long-term outcomes in Japanese sarcoidosis patients? | Respiratory medicine | 149 |  | 01-Aug | eng | https://pubmed.ncbi.nlm.nih.gov/30885423/ | 10.1016/j.rmed.2019.01.001 |
| TH/DZH | full text | no patient level ultrasound data, otherwise very interesting study | 2019 | Tauseef A and Ijaz F and Chaudhary FA and Ali Z and Akram T and Aftab RK and Ahmad G | Role of Interleukin-10 and Abdominopelvic Ultrasound as a Potential Predictor of Disease Severity in Dengue Hemorrhagic Fever. | Cureus | 11 | 7 | e5249 | eng | https://pubmed.ncbi.nlm.nih.gov/31572634/ | 10.7759/cureus.5249 |
| TH/DZH | full text | INCLUDED: original patient level data on US in VHF | 2019 | Tavares MA and Jo√£o GAP and Bastos MS and Gimaque JBL and Almeida ACG and Ngo TT and Bahamon C and Baia-da-Silva DC and Monteiro WM and Mour√£o MPG and Lacerda MVG | Clinical relevance of gallbladder wall thickening for dengue severity: A cross-sectional study. | PloS one | 14 | 8 | e0218939 | eng | https://pubmed.ncbi.nlm.nih.gov/31469845/ | 10.1371/journal.pone.0218939 |
| TH/DZH | title/abstract | US in VHF, but case study or series | 2019 | Vianello S and Silva de Souza G and Maia M and Belfort R Jr and de Oliveira Dias JR | Ocular Findings in Yellow Fever Infection. | JAMA ophthalmology | 137 | 3 | 300-304 | eng | https://pubmed.ncbi.nlm.nih.gov/30629101/ | 10.1001/jamaophthalmol.2018.6408 |
| TH/DZH | title/abstract | did not have primary data on US in VHF | 2019 | Yao PP and Chen G and Xu F and Yang ZR and Chen C and Sun YS and Lu HJ and Pang WL and Zhang Y and Zhu HP and Xiang HQ | [Genotype and evolution of hantavirus in Tiantai of Zhejiang province, 2011-2018]. | Zhonghua liu xing bing xue za zhi = Zhonghua liuxingbingxue zazhi | 40 | 10 | 1285-1290 | chi | https://pubmed.ncbi.nlm.nih.gov/31658532/ | 10.3760/cma.j.issn.0254-6450.2019.10.021 |
| TH/DZH | full text | INCLUDED: original patient level data on US in VHF | 2020 | Adil B and Rabbani A and Ahmed S and Arshad I Sr and Khalid MA | Gall Bladder Wall Thickening in Dengue Fever - Aid in Labelling Dengue Hemorrhagic Fever and a Marker of Severity. | Cureus | 12 | 11 | e11331 | eng | https://pubmed.ncbi.nlm.nih.gov/33304669/ | 10.7759/cureus.11331 |
| TH/DZH | title/abstract | did not have primary data on US in VHF | 2020 | Agi A and Junin R and Arsad A and Abbas A and Gbadamosi A and Azli NB and Oseh J | Ultrasound-assisted weak-acid hydrolysis of crystalline starch nanoparticles for chemical enhanced oil recovery. | International journal of biological macromolecules | 148 |  | 1251-1271 | eng | https://pubmed.ncbi.nlm.nih.gov/31760018/ | 10.1016/j.ijbiomac.2019.10.099 |
| TH/DZH | title/abstract | did not have primary data on US in VHF | 2020 | Aijazi I and Al Shama FMA and Shandala Y and Varghese RM | Crimean-Congo haemorrhagic fever presenting with acute compartment syndrome of the extremities (think beyond normal infections). | BMJ case reports | 13 | 2 |  | eng | https://pubmed.ncbi.nlm.nih.gov/32033997/ | 10.1136/bcr-2019-232323 |
| TH/DZH | full text | ultrasound only completed in selected patients with elevated cardiac enzymes, also possible duplicate | 2020 | Bhatt M and Soneja M and Farooqui FA and Singla P and Vikram NK and Biswas A and Roy A and Wig N | Myocarditis in admitted patients with dengue fever. | Infection | 48 | 6 | 899-903 | eng | https://pubmed.ncbi.nlm.nih.gov/32780310/ | 10.1007/s15010-020-01500-w |
| TH/DZH | title/abstract | did not have primary data on US in VHF | 2020 | Bietsch K and Williamson J and Reeves M | Family Planning During and After the West African Ebola Crisis. | Studies in family planning | 51 | 1 | 71-86 | eng | https://pubmed.ncbi.nlm.nih.gov/32180246/ | 10.1111/sifp.12110 |
| TH/DZH | title/abstract | did not have primary data on US in VHF | 2020 | Chadalawada S and Sillau S and Archuleta S and Mundo W and Bandali M and Parra-Henao G and Rodriguez-Morales AJ and Villamil-Gomez WE and Su√°rez JA and Shapiro L and Hotez PJ and Woc-Colburn L and DeSanto K and Rassi A Jr and Franco-Paredes C and Henao-Mart√≠nez AF | Risk of Chronic Cardiomyopathy Among Patients With the Acute Phase or Indeterminate Form of Chagas Disease: A Systematic Review and Meta-analysis. | JAMA network open | 3 | 8 | e2015072 | eng | https://pubmed.ncbi.nlm.nih.gov/32865573/ | 10.1001/jamanetworkopen.2020.15072 |
| TH/DZH | title/abstract | US in VHF, but case study or series | 2020 | Dandeniya CL and Gawarammana IB and Weerakoon G | Coronary Artery Spasms Mimicking Acute ST-Elevation Myocardial Infarction in Dengue Haemorrhagic Fever. | Case reports in infectious diseases | 2020 |  | 6310569 | eng | https://pubmed.ncbi.nlm.nih.gov/32095297/ | 10.1155/2020/6310569 |
| TH/DZH | full text | INCLUDED: original patient level data on US in VHF | 2020 | Dussart P and Duong V and Bleakley K and Fortas C and Lorn Try P and Kim KS and Choeung R and In S and Andries AC and Cantaert T and Flamand M and Buchy P and Sakuntabhai A | Comparison of dengue case classification schemes and evaluation of biological changes in different dengue clinical patterns in a longitudinal follow-up of hospitalized children in Cambodia. | PLoS neglected tropical diseases | 14 | 9 | e0008603 | eng | https://pubmed.ncbi.nlm.nih.gov/32925941/ | 10.1371/journal.pntd.0008603 |
| TH/DZH | title/abstract | did not have primary data on US in VHF | 2020 | Godebo TR and Jeuland M and Tekle-Haimanot R and Shankar A and Alemayehu B and Assefa G and Whitford G and Wolfe A | Bone quality in fluoride-exposed populations: A novel application of the ultrasonic method. | Bone reports | 12 |  | 100235 | eng | https://pubmed.ncbi.nlm.nih.gov/31890757/ | 10.1016/j.bonr.2019.100235 |
| TH/DZH | title/abstract | did not have primary data on US in VHF | 2020 | Kodikara I and Abeysekara I and Gamage D and Ilayperuma I | Assessment of 2D ultrasound fluid volume estimation accuracy in different shaped objects: an in vitro study. | Acta radiologica (Stockholm, Sweden : 1987) | 61 | 2 | 253-259 | eng | https://pubmed.ncbi.nlm.nih.gov/31177804/ | 10.1177/0284185119854198 |
| TH/DZH | title/abstract | did not have primary data on US in VHF | 2020 | Kodikara I and Gamage D and Abeysekara I and Ilayperuma I | Impact of volume calculation formulae on volume estimation accuracy of different shaped objects: an in vitro ultrasound and CT study. | Acta radiologica (Stockholm, Sweden : 1987) | 61 | 10 | 1414-1420 | eng | https://pubmed.ncbi.nlm.nih.gov/32013542/ | 10.1177/0284185120901505 |
| TH/DZH | title/abstract | did not have primary data on US in VHF | 2020 | Lebecque O and Dupont M | Puumala hantavirus: an imaging review. | Acta radiologica (Stockholm, Sweden : 1987) | 61 | 8 | 1072-1079 | eng | https://pubmed.ncbi.nlm.nih.gov/31805769/ | 10.1177/0284185119889564 |
| TH/DZH | title/abstract | did not have primary data on US in VHF | 2020 | Leone D and Cina A and Tosello F and Sabia L and Vallelonga F and Avenatti E and Astarita A and Mingrone G and Veglio F and Ridolfi L and Milan A | Proximal aortic diameter evolution in hypertensive patients with mild-to-moderate aortic dilatation: a 5-year follow-up experience. | Journal of hypertension | 38 | 4 | 716-722 | eng | https://pubmed.ncbi.nlm.nih.gov/31764583/ | 10.1097/HJH.0000000000002315 |
| TH/DZH | full text | INCLUDED: original patient level data on US in VHF | 2020 | Osquee HO and Taghizadeh S and Haghdoost M and Pourjafar H and Ansari F | New Insight for the Prognosis of CCHF: Clinical, Laboratory and Sonography Findings. | Current medical imaging | 16 | 9 | 1125-1130 | eng | https://pubmed.ncbi.nlm.nih.gov/33342399/ | 10.2174/1573405615666191111115354 |
| TH/DZH | title/abstract | did not have primary data on US in VHF | 2020 | Ozmen C and Deveci OS and Karaaslan MB and Baydar O and Akray A and Deniz A and Cagliyan CE and Hanta I and Usal A | Predictive value of plasma copeptin level for diagnosis and mortality of pulmonary embolism. | Revista da Associacao Medica Brasileira (1992) | 66 | 12 | 1645-1650 | eng | https://pubmed.ncbi.nlm.nih.gov/33331571/ | 10.1590/1806-9282.66.12.1645 |
| TH/DZH | full text | INCLUDED: original patient level data on US in VHF | 2020 | Rathore APS and Senanayake M and Athapathu AS and Gunasena S and Karunaratna I and Leong WY and Lim T and Mantri CK and Wilder-Smith A and St John AL | Serum chymase levels correlate with severe dengue warning signs and clinical fluid accumulation in hospitalized pediatric patients. | Scientific reports | 10 | 1 | 11856 | eng | https://pubmed.ncbi.nlm.nih.gov/32678248/ | 10.1038/s41598-020-68844-z |
| TH/DZH | title/abstract | did not have primary data on US in VHF | 2020 | Schwabe D and Kellner B and Henkel D and Pilligrath HJ and Krummer S and Zach S and Rohrbeck C and Diefenbach M and Veldman A | Long-Distance Aeromedical Transport of Patients with COVID-19 in Fixed-Wing Air Ambulance Using a Portable Isolation Unit: Opportunities, Limitations and Mitigation Strategies. | Open access emergency medicine : OAEM | 12 |  | 411-419 | eng | https://pubmed.ncbi.nlm.nih.gov/33269009/ | 10.2147/OAEM.S277678 |
| TH/DZH | title/abstract | did not have primary data on US in VHF | 2020 | Tsvetkov VA and Krutikov ES and Chistyakova SI | [Subclinical left ventricular dysfunction in patients with type 2 diabetes mellitus]. | Problemy endokrinologii | 66 | 1 | 56-63 | rus | https://pubmed.ncbi.nlm.nih.gov/33351313/ | 10.14341/probl12359 |
| TH/DZH | title/abstract | US in VHF, but case study or series | 2020 | Vanichkulbodee A and Othong R | Man With Diarrhea and Fever. | Annals of emergency medicine | 76 | 1 | 108-118 | eng | https://pubmed.ncbi.nlm.nih.gov/32591116/ | 10.1016/j.annemergmed.2020.01.020 |
| TH/DZH | title/abstract | did not have primary data on US in VHF | 2020 | Walker CL and Ehinger N and Mason B and Oler E and Little ME and Ohuma EO and Papageorghiou AT and Nayeri U and Curry C and Adams Waldorf KM | Ultrasound prediction of Zika virus-associated congenital injury using the profile of fetal growth. | PloS one | 15 | 5 | e0233023 | eng | https://pubmed.ncbi.nlm.nih.gov/32401826/ | 10.1371/journal.pone.0233023 |
| TH/DZH | full text | INCLUDED: original patient level data on US in VHF | 2020 | Xin Tian C and Baharuddin KA and Shaik Farid AW and Andey R and Ridzuan MI and Siti-Azrin AH | Ultrasound findings of plasma leakage as imaging adjunct in clinical management of dengue fever without warning signs. | The Medical journal of Malaysia | 75 | 6 | 635-641 | eng | https://pubmed.ncbi.nlm.nih.gov/33219170/ |  |
| TH/DZH | full text | INCLUDED: original patient level data on US in VHF | 2021 | Abhinayaa J and James S and Jebaraj R and Vinoth PN | Incidence of Cardiac Manifestations in Children with Dengue Fever: A Cross-sectional Study. | Rambam Maimonides medical journal | 12 | 2 |  | eng | https://pubmed.ncbi.nlm.nih.gov/33938801/ | 10.5041/RMMJ.10436 |
| TH/DZH | title/abstract | did not have primary data on US in VHF | 2021 | Alemad SA and Halboup AM and Aladeeb K and Al-Saleh M and Al-Kufiley N | Coinfection with dengue and hepatitis A complicated with infective endocarditis in a Yemeni patient: a case report. | Journal of medical case reports | 15 | 1 | 450 | eng | https://pubmed.ncbi.nlm.nih.gov/34507614/ | 10.1186/s13256-021-03069-w |
| TH/DZH | title/abstract | did not have primary data on US in VHF | 2021 | Baillieul A and Le TL and Rouland JF | Acute angle-closure glaucoma with choroidal effusion revealing a hantavirus infection: Description of ultrasound biomicroscopy imagery and optical coherence tomography Visante. | European journal of ophthalmology | 31 | 1 | NP4-NP8 | eng | https://pubmed.ncbi.nlm.nih.gov/31256684/ | 10.1177/1120672119858895 |
| TH/DZH | title/abstract | did not have primary data on US in VHF | 2021 | Beressa TB and Deyno S and Mtewa AG and Aidah N and Tuyiringire N and Lukubye B and Weisheit A and Tolo CU and Ogwang PE | Potential Benefits of Antiviral African Medicinal Plants in the Management of Viral Infections: Systematic Review. | Frontiers in pharmacology | 12 |  | 682794 | eng | https://pubmed.ncbi.nlm.nih.gov/35002686/ | 10.3389/fphar.2021.682794 |
| TH/DZH | title/abstract | did not have primary data on US in VHF | 2021 | Biasucci DG and Buonsenso D and Piano A and Bonadia N and Vargas J and Settanni D and Bocci MG and Grieco DL and Carnicelli A and Scoppettuolo G and Eleuteri D and DE Pascale G and Pennisi MA and Franceschi F and Antonelli M | Lung ultrasound predicts non-invasive ventilation outcome in COVID-19 acute respiratory failure: a pilot study. | Minerva anestesiologica | 87 | 9 | 1006-1016 | eng | https://pubmed.ncbi.nlm.nih.gov/34263580/ | 10.23736/S0375-9393.21.15188-0 |
| TH/DZH | title/abstract | did not have primary data on US in VHF | 2021 | Calder√≥n-Gerstein W and Bruno-Huam√°n A and Dami√°n-Mucha M and Huayllani-Flores L | Predictive capacity of the brain natriuretic peptide in the screening of heart failure in a high altitude population. | Respiratory physiology & neurobiology | 289 |  | 103654 | eng | https://pubmed.ncbi.nlm.nih.gov/33746081/ | 10.1016/j.resp.2021.103654 |
| TH/DZH | title/abstract | did not have primary data on US in VHF | 2021 | Cesareo M and Sabia L and Leone D and Avenatti E and Astarita A and Mingrone G and Airale L and Veglio F and Vallelonga F and Milan A | Local transversal aortic strain is impaired in ascending aorta dilatation. | Journal of hypertension | 39 | 7 | 1402-1411 | eng | https://pubmed.ncbi.nlm.nih.gov/33399306/ | 10.1097/HJH.0000000000002757 |
| TH/DZH | title/abstract | did not have primary data on US in VHF | 2021 | Dewan N and Zuluaga D and Osorio L and Krienke ME and Bakker C and Kirsch J | Ultrasound in Dengue: A Scoping Review. | The American journal of tropical medicine and hygiene | 104 | 3 | 826-835 | eng | https://pubmed.ncbi.nlm.nih.gov/33534759/ | 10.4269/ajtmh.20-0103 |
| TH/DZH | full text | INCLUDED: original patient level data on US in VHF | 2021 | Donaldson CD and de Mel S and Clarice CSH and Thilakawardana BU and de Mel P and Shalindi M and Samarasinghe U and de Mel C and Chandrasena L and Wijesinha RS and Wijesinha ANI and Yip C and Yap ES and Seneviratne SL and Abeysuriya V | Admission ultrasonography as a predictive tool for thrombocytopenia and disease severity in dengue infection. | Transactions of the Royal Society of Tropical Medicine and Hygiene | 115 | 12 | 1396-1402 | eng | https://pubmed.ncbi.nlm.nih.gov/33885813/ | 10.1093/trstmh/trab064 |
| TH/DZH | title/abstract | did not have primary data on US in VHF | 2021 | Dronamraju SS and Gaidhane SA and Mahalaqqa KN and Gaidhane AM and Andhale AG and Quazi ZS | Splenic Artery Embolization in Subcapsular Splenic Hematoma Secondary to Dengue Hemorrhagic Fever. | Journal of global infectious diseases | 13 | 3 | 145-147 | eng | https://pubmed.ncbi.nlm.nih.gov/34703156/ | 10.4103/jgid.jgid_140_20 |
| TH/DZH | title/abstract | did not have primary data on US in VHF | 2021 | Espinoza CR and Ruiz CAJ and Ramos OPF and Solano MAQ and Qui√±onez GH and Mallma NES | Optimization of the ultrasoud-assisted extraction of saponins from quinoa (Chenopodium quinoa Wild) using response surface methodology. | Acta scientiarum polonorum. Technologia alimentaria | 20 | 1 | 17-23 | eng | https://pubmed.ncbi.nlm.nih.gov/33449516/ | 10.17306/J.AFS.0859 |
| TH/DZH | title/abstract | did not have primary data on US in VHF | 2021 | Ferdous A and Hossain MM and Afrin M | Severe Dengue With Multisystem Inflammatory Syndrome in Children Due to COVID-19: A Co-infection Case Series. | Cureus | 13 | 11 | e19516 | eng | https://pubmed.ncbi.nlm.nih.gov/34926034/ | 10.7759/cureus.19516 |
| TH/DZH | title/abstract | did not have primary data on US in VHF | 2021 | Ferdous A and Hossain MM and Afrin M and Shirin M | Dengue With COVID-19: Associated With Co-infection and Multiple Organ Dysfunction in a Child. | Cureus | 13 | 12 | e20763 | eng | https://pubmed.ncbi.nlm.nih.gov/34987943/ | 10.7759/cureus.20763 |
| TH/DZH | title/abstract | US in VHF, but case study or series | 2021 | Freise NF and Jensen B and Keitel V and Luedde T | Gallbladder Wall Thickening associated with Dengue Shock Syndrome in a German traveller - no indication for surgical therapy - a case report. | Tropical diseases, travel medicine and vaccines | 7 | 1 | 23 | eng | https://pubmed.ncbi.nlm.nih.gov/34344481/ | 10.1186/s40794-021-00148-0 |
| TH/DZH | title/abstract | US in VHF, but case study or series | 2021 | Kerget F and Demird√∂ƒüen ≈ûO and Kerget B | Case Report: A Rare Case of Crimean-Congo Hemorrhagic Fever Associated with Epididymo-Orchitis. | The American journal of tropical medicine and hygiene | 104 | 3 | 1055-1057 | eng | https://pubmed.ncbi.nlm.nih.gov/33399048/ | 10.4269/ajtmh.20-1143 |
| TH/DZH | full text | INCLUDED: original patient level data on US in VHF | 2021 | Koyama H and Chierakul W and Charunwatthana P and Sanguanwongse N and Phonrat B and Silachamroon U and Dondorp AM | Lung Ultrasound Findings of Patients with Dengue Infection: A Prospective Observational Study. | The American journal of tropical medicine and hygiene | 105 | 3 | 766-770 | eng | https://pubmed.ncbi.nlm.nih.gov/34280132/ | 10.4269/ajtmh.20-1274 |
| TH/DZH | title/abstract | did not have primary data on US in VHF | 2021 | Lee SL and Ng CY and Sidhu J | Infected pancreatic pseudocyst following severe dengue infection. | The Medical journal of Malaysia | 76 | 6 | 927-929 | eng | https://pubmed.ncbi.nlm.nih.gov/34806688/ |  |
| TH/DZH | title/abstract | did not have primary data on US in VHF | 2021 | Leone D and Airale L and Bernardi S and Mingrone G and Astarita A and Cesareo M and Sabia L and Avenatti E and Tosello F and Bruno G and Catarinella C and Venturelli V and Giordana C and Veglio F and Vallelonga F and Milan A | Prognostic role of the ascending aorta dilatation in patients with arterial hypertension. | Journal of hypertension | 39 | 6 | 1163-1169 | eng | https://pubmed.ncbi.nlm.nih.gov/33710167/ | 10.1097/HJH.0000000000002752 |
| TH/DZH | full text | INCLUDED: original patient level data on US in VHF | 2021 | Mansanguan C and Hanboonkunupakarn B and Muangnoicharoen S and Huntrup A and Poolcharoen A and Mansanguan S and Piyaphanee W and Phumratanaprapin W | Cardiac evaluation in adults with dengue virus infection by serial echocardiography. | BMC infectious diseases | 21 | 1 | 940 | eng | https://pubmed.ncbi.nlm.nih.gov/34507547/ | 10.1186/s12879-021-06639-x |
| TH/DZH | title/abstract | did not have primary data on US in VHF | 2021 | Marb√°n-Castro E and Gonc√© A and Fumad√≥ V and Romero-Acevedo L and Bardaj√≠ A | Zika virus infection in pregnant women and their children: A review. | European journal of obstetrics, gynecology, and reproductive biology | 265 |  | 162-168 | eng | https://pubmed.ncbi.nlm.nih.gov/34508989/ | 10.1016/j.ejogrb.2021.07.012 |
| TH/DZH | full text | INCLUDED: original patient level data on US in VHF | 2021 | Neves YCS and Castro-Lima VAC and Solla DJF and Ogata VSM and Pereira FL and Araujo JM and Ho YL and Chammas MC | Ultrasound Findings and Laboratory Predictors of Early Mortality in Patients With Severe Yellow Fever. | AJR. American journal of roentgenology | 216 | 5 | 1392-1399 | eng | https://pubmed.ncbi.nlm.nih.gov/33703928/ | 10.2214/AJR.20.23455 |
| TH/DZH | title/abstract | did not have primary data on US in VHF | 2021 | Neves YCS and Castro-Lima VAC and Solla DJF and Ogata VSM and Pereira FL and Araujo JM and Nastri ACS and Ho YL and Chammas MC | Staging liver fibrosis after severe yellow fever with ultrasound elastography in Brazil: A six-month follow-up study. | PLoS neglected tropical diseases | 15 | 7 | e0009594 | eng | https://pubmed.ncbi.nlm.nih.gov/34283826/ | 10.1371/journal.pntd.0009594 |
| TH/DZH | title/abstract | did not have primary data on US in VHF | 2021 | Prado MB Jr and Narito KM and Adiao KJB | Anti-GM1 IgM antibody positive axonal variant of Guillain-Barre-syndrome in a pediatric patient with dengue fever. | Journal of neuroimmunology | 355 |  | 577572 | eng | https://pubmed.ncbi.nlm.nih.gov/33862418/ | 10.1016/j.jneuroim.2021.577572 |
| TH/DZH | title/abstract | did not have primary data on US in VHF | 2021 | Quan J and Zhang X and Ding Y and Li S and Qiu Y and Wang R and Zhou X | Cucurbit[7]uril as a Broad-Spectrum Antiviral Agent against Diverse RNA Viruses. | Virologica Sinica | 36 | 5 | 1165-1176 | eng | https://pubmed.ncbi.nlm.nih.gov/34037947/ | 10.1007/s12250-021-00404-9 |
| TH/DZH | full text | no patient level ultrasound data | 2021 | Shah C and Vijayaraghavan G and Kartha CC | Spectrum of cardiac involvement in patients with dengue fever. | International journal of cardiology | 324 |  | 180-185 | eng | https://pubmed.ncbi.nlm.nih.gov/32931859/ | 10.1016/j.ijcard.2020.09.034 |
| TH/DZH | title/abstract | US in VHF, but case study or series | 2021 | Shamil PK and Kumar R and Kumar A and Chakrabarti S | A 25-Year-Old Man With Dengue Fever and Hypoxemic Respiratory Failure. | Chest | 159 | 6 | e417-e419 | eng | https://pubmed.ncbi.nlm.nih.gov/34099161/ | 10.1016/j.chest.2020.03.094 |
| TH/DZH | full text | INCLUDED: original patient level data on US in VHF | 2021 | Sigera PC and Weeratunga P and Deepika Fernando S and Lakshitha De Silva N and Rodrigo C and Rajapakse S | Rational use of ultrasonography with triaging of patients to detect dengue plasma leakage in resource limited settings: a prospective cohort study. | Tropical medicine & international health : TM & IH | 26 | 8 | 993-1001 | eng | https://pubmed.ncbi.nlm.nih.gov/33892519/ | 10.1111/tmi.13594 |
| TH/DZH | title/abstract | did not have primary data on US in VHF | 2021 | Singh V and Nayyer PS and Rana V | Dengue Fever: Transient Variable AV Conduction Block (2:1 with Mobitz Type I Second Degree) During Recovery Phase. | The Journal of the Association of Physicians of India | 69 | 4 | 11-Dec | eng | https://pubmed.ncbi.nlm.nih.gov/34470195/ |  |
| TH/DZH | title/abstract | US in VHF, but case study or series | 2021 | Thadchanamoorthy V and Dayasiri K | Child with dengue haemorrhagic fever complicated by ileocaecal intussusception. | BMJ case reports | 14 | 3 |  | eng | https://pubmed.ncbi.nlm.nih.gov/33727286/ | 10.1136/bcr-2020-237697 |
| TH/DZH | title/abstract | did not have primary data on US in VHF | 2021 | Zeng M and Chen Y and Zhao B | Higher liver stiffness in patients with chronic congestive heart failure: data from NHANES with liver ultrasound transient elastography. | Annals of palliative medicine | 10 | 6 | 6859-6866 | eng | https://pubmed.ncbi.nlm.nih.gov/34237983/ | 10.21037/apm-21-1362 |
| TH/DZH | title/abstract | did not have primary data on US in VHF | 2022 | Alvarado-Domenech LI and Rivera-Amill V and Appleton AA and Rosario-Villafa√±e V and Repollet-Carrer I and Borges-Rodr√≠guez M and P√©rez-Rodr√≠guez NM and Olivieri-Ramos O and Gonz√°lez M and Gonz√°lez-Montalvo C and Mu√±iz-Forestier W and Vargas-Lasalle L and P√©rez-Padilla J and Paz-Bailey G and Rodr√≠guez-Rabassa M | Early Childhood Neurodevelopmental Outcomes in Children with Prenatal Zika Virus Exposure: A Cohort Study in Puerto Rico. | The Journal of pediatrics | 247 |  | 38-45.e5 | eng | https://pubmed.ncbi.nlm.nih.gov/35577118/ | 10.1016/j.jpeds.2022.05.016 |
| TH/DZH | title/abstract | did not have primary data on US in VHF | 2022 | Arif A and Abdul Razzaque MR and Kogut LM and Tebha SS and Shahid F and Essar MY | Expanded dengue syndrome presented with rhabdomyolysis, compartment syndrome, and acute kidney injury: A case report. | Medicine | 101 | 7 | e28865 | eng | https://pubmed.ncbi.nlm.nih.gov/35363190/ | 10.1097/MD.0000000000028865 |
| TH/DZH | full text | no patient level ultrasound data | 2022 | Baqi A and Ur Rehman F and Memon PS and Omair SF | Prevalence and Outcomes of Myocarditis in Dengue-Infected Patients Admitted to a Tertiary Care Hospital of Low-Middle Income Country. | Global heart | 17 | 1 | 44 | eng | https://pubmed.ncbi.nlm.nih.gov/35837358/ | 10.5334/gh.1129 |
| TH/DZH | title/abstract | did not have primary data on US in VHF | 2022 | Brix S and Kaiser S and L√∂rz AN and Le Saout M and Schumacher M and Bonk F and Egilsdottir H and Olafsdottir SH and Tandberg AHS and Taylor J and Tewes S and Xavier JR and Linse K | Habitat variability and faunal zonation at the √Ügir Ridge, a canyon-like structure in the deep Norwegian Sea. | PeerJ | 10 |  | e13394 | eng | https://pubmed.ncbi.nlm.nih.gov/35726260/ | 10.7717/peerj.13394 |
| TH/DZH | title/abstract | did not have primary data on US in VHF | 2022 | Cherevashchenko LA and Dadova LY and Tsogoev AS and Bobrik YV and Efimenko NV and Starokozhko LE and Amiyants VY and Gaidamaka II | [Physical factors in the medical rehabilitation of patients with chronic cerebral ischemia in the outpatient stage]. | Voprosy kurortologii, fizioterapii, i lechebnoi fizicheskoi kultury | 99 | 6 | Dec-18 | rus | https://pubmed.ncbi.nlm.nih.gov/36511461/ | 10.17116/kurort20229906212 |
| TH/DZH | title/abstract | US in VHF, but case study or series | 2022 | Flor MA and Andrade JV and Bucaram JA | Acute Pancreatitis Secondary to Dengue Fever: An Uncommon Presentation of a Common Endemic Illness. | Case reports in infectious diseases | 2022 |  | 9540705 | eng | https://pubmed.ncbi.nlm.nih.gov/36561472/ | 10.1155/2022/9540705 |
| TH/DZH | full text | cases not confirmed | 2022 | Gleeson T and Pagnarith Y and Habsreng E and Lindsay R and Hill M and Sanseverino A and Patel V and Gaspari R | Dengue Management in Triage using Ultrasound in children from Cambodia: a prospective cohort study. | The Lancet regional health. Western Pacific | 19 |  | 100371 | eng | https://pubmed.ncbi.nlm.nih.gov/35079724/ | 10.1016/j.lanwpc.2021.100371 |
| TH/DZH | title/abstract | did not have primary data on US in VHF | 2022 | Gleeson T and Pagnarith Y and Habsreng E and Lindsay R and Hill M and Sanseverino A and Patel V and Gaspari R | The authors' reply to comments on "dengue management in triage using ultrasound in children from Cambodia: A prospective cohort study". | The Lancet regional health. Western Pacific | 25 |  | 100559 | eng | https://pubmed.ncbi.nlm.nih.gov/35990712/ | 10.1016/j.lanwpc.2022.100559 |
| TH/DZH | full text | no patient level ultrasound data | 2022 | Gupta S and Gupta M and Kashyap JR and Arora SK | Early cardiovascular involvement in dengue fever: A prospective study with two-dimensional speckle tracking echocardiography. | Tropical doctor | 52 | 2 | 285-292 | eng | https://pubmed.ncbi.nlm.nih.gov/35098804/ | 10.1177/00494755221076686 |
| TH/DZH | title/abstract | US in VHF, but case study or series | 2022 | Gurung S and Karki S and Khadka M and Gurung S and Dhakal S | Acute acalculous cholecystitis in a patient with dengue fever: A case report. | Annals of medicine and surgery (2012) | 84 |  | 104960 | eng | https://pubmed.ncbi.nlm.nih.gov/36582863/ | 10.1016/j.amsu.2022.104960 |
| TH/DZH | title/abstract | did not have primary data on US in VHF | 2022 | Halstead SB and Tissera H and Kumarasinghe M and Fernando L | Comments on "Dengue management in triage using ultrasound in children from Cambodia: A prospective cohort study". | The Lancet regional health. Western Pacific | 25 |  | 100527 | eng | https://pubmed.ncbi.nlm.nih.gov/36091881/ | 10.1016/j.lanwpc.2022.100527 |
| TH/DZH | full text | INCLUDED: original patient level data on US in VHF | 2022 | Ibrahim MA and Hamzah SS and Md Noor J and Mohamad MIK and Mokhtar MF and Isa MR and Abdul Rani MF | The association of ultrasound assessment of gallbladder wall thickness with dengue fever severity. | The ultrasound journal | 14 | 1 | 13 | eng | https://pubmed.ncbi.nlm.nih.gov/35325315/ | 10.1186/s13089-022-00262-w |
| TH/DZH | full text | no ultrasound results | 2022 | Jaspard M and Mulangu S and Juchet S and Serra B and Dicko I and Lang HJ and Baka BM and Komanda GM and Katsavara JM and Kabuni P and Mambu FM and Isnard M and Vanhecke C and Letord A and Dieye I and Patterson-Lomba O and Mbaya OT and Isekusu F and Mangala D and Biampata JL and Kitenge R and Kinda M and Anglaret X and Muyembe JJ and Kojan R and Ezzedine K and Malvy D | Development of the PREDS score to predict in-hospital mortality of patients with Ebola virus disease under advanced supportive care: Results from the EVISTA cohort in the Democratic Republic of the Congo. | EClinicalMedicine | 54 |  | 101699 | eng | https://pubmed.ncbi.nlm.nih.gov/36263398/ | 10.1016/j.eclinm.2022.101699 |
| TH/DZH | full text | no patient level ultrasound data | 2022 | Kaagaard MD and Wegener A and Gomes LC and Holm AE and Lima KO and Matos LO and Vieira IVM and de Souza RM and Vestergaard LS and Marinho CRF and Dos Santos FB and Biering-S√∏rensen T and Silvestre OM and Brainin P | Potential role of transthoracic echocardiography for screening LV systolic dysfunction in patients with a history of dengue infection. A cross-sectional and cohort study and review of the literature. | PloS one | 17 | 11 | e0276725 | eng | https://pubmed.ncbi.nlm.nih.gov/36399460/ | 10.1371/journal.pone.0276725 |
| TH/DZH | title/abstract | did not have primary data on US in VHF | 2022 | Kilicdag H and Torer B and Demir S and Hanta D and Akbas T and Mert MK and Soker G | Impact of antenatal corticosteroid exposure on thymus size in premature infants. | Archives de pediatrie : organe officiel de la Societe francaise de pediatrie | 29 | 1 | 67-71 | eng | https://pubmed.ncbi.nlm.nih.gov/34763993/ | 10.1016/j.arcped.2021.09.015 |
| TH/DZH | title/abstract | did not have primary data on US in VHF | 2022 | L√≥pez-Vi√±as L and Vega-Villar J and Roc√≠o-Mart√≠n E and Garc√≠a-Garc√≠a P and De La Rosa Santiago E and Galv√°n-Rom√°n JM and Wix-Ramos R | Diaphragm impairment in patients admitted for severe COVID-19. | European journal of translational myology | 32 | 2 |  | eng | https://pubmed.ncbi.nlm.nih.gov/35727218/ | 10.4081/ejtm.2022.10460 |
| TH/DZH | title/abstract | did not have primary data on US in VHF | 2022 | Lebecque O and Falticeanu A and Mulquin N and Dupont M | Abdominal CT findings in Puumala hantavirus-infected patients. | Abdominal radiology (New York) | 47 | 7 | 2552-2559 | eng | https://pubmed.ncbi.nlm.nih.gov/35441863/ | 10.1007/s00261-022-03467-8 |
| TH/DZH | title/abstract | did not have primary data on US in VHF | 2022 | Mahajan A and Yadav S and Maheshwari A and Mahto D and Divya K and Ackshaya R and Meena H and Shakya S and Kumar V | Profile of Children with Kawasaki Disease Associated with Tropical Infections. | Indian journal of pediatrics | 89 | 8 | 759-764 | eng | https://pubmed.ncbi.nlm.nih.gov/34935098/ | 10.1007/s12098-021-03953-9 |
| TH/DZH | title/abstract | did not have primary data on US in VHF | 2022 | Mathis A and Collins D and Dobo S and Walling DM and Sheridan WP and Taylor R | Pharmacokinetics and Safety of the Nucleoside Analog Antiviral Drug Galidesivir Administered to Healthy Adult Subjects. | Clinical pharmacology in drug development | 11 | 4 | 467-474 | eng | https://pubmed.ncbi.nlm.nih.gov/35182042/ | 10.1002/cpdd.1037 |
| TH/DZH | title/abstract | did not have primary data on US in VHF | 2022 | Moraes AG and Ayo CM and Elp√≠dio LNS and Souza VH and Yamanaka AHU and Nogueira ML and Passos SD and Brand√£o CC and Mattos LC and Amaral GCD and Neto QAL and Visentainer JEL | HLA-G, LILRB1 and LILRB2 Variants in Zika Virus Transmission from Mother to Child in a Population from South and Southeast of Brazil. | Current issues in molecular biology | 44 | 7 | 2783-2793 | eng | https://pubmed.ncbi.nlm.nih.gov/35877415/ | 10.3390/cimb44070191 |
| TH/DZH | full text | INCLUDED: original patient level data on US in VHF | 2022 | Nerella S and Sarkar UK and Namdeo H | Electrocardiographic and echocardiographic findings in children with dengue infection. | Journal of family medicine and primary care | 11 | 6 | 2334-2339 | eng | https://pubmed.ncbi.nlm.nih.gov/36119231/ | 10.4103/jfmpc.jfmpc_1280_21 |
| TH/DZH | title/abstract | did not have primary data on US in VHF | 2022 | Pandeya A and Upadhyay D and Oli B and Parajuli M and Silwal N and Shrestha A and Gautam N and Gajurel BP | Dengue encephalitis featuring "double-doughnut" sign - A case report. | Annals of medicine and surgery (2012) | 78 |  | 103939 | eng | https://pubmed.ncbi.nlm.nih.gov/35734672/ | 10.1016/j.amsu.2022.103939 |
| TH/DZH | full text | full text not available, case series of patient with renal rupture | 2022 | Razi RZ and Ahmed AM and Sultana S and Amin K and Ahsan M and Akter P and Paul P and Sangma MA and Ferdousi RA and Jahan F and Hasan MS and Ahsan SB | Clinical and Haematological Findings in Dengue Fever. | Mymensingh medical journal : MMJ | 31 | 2 | 344-349 | eng | https://pubmed.ncbi.nlm.nih.gov/35383748/ |  |
| TH/DZH | full text | INCLUDED: original patient level data on US in VHF | 2022 | Riswari SF and Budiman MF and Darmayanti D and Ernawati and Prodjosoewojo S and Susandi E and Oehadian A and Alisjahbana B | A Comparison of the Accuracy of Handheld Hemoglobinometer and Hematocrit Measurements for Detecting Plasma Leakage in Dengue Hemorrhagic Fever. | International journal of general medicine | 15 |  | 2589-2595 | eng | https://pubmed.ncbi.nlm.nih.gov/35282645/ | 10.2147/IJGM.S343017 |
| TH/DZH | title/abstract | did not have primary data on US in VHF | 2022 | Santana EFM and Casati MFM and Geraldo MSP and Werner H and Araujo J√∫nior E | Intrauterine Zika virus infection: review of the current findings with emphasis in the prenatal and postnatal brain imaging diagnostic methods. | The journal of maternal-fetal & neonatal medicine : the official journal of the European Association of Perinatal Medicine, the Federation of Asia and Oceania Perinatal Societies, the International Society of Perinatal Obstetricians | 35 | 25 | 6062-6068 | eng | https://pubmed.ncbi.nlm.nih.gov/33781162/ | 10.1080/14767058.2021.1904874 |
| TH/DZH | title/abstract | US in VHF, but case study or series | 2022 | Setyawati AN and Tjahjono Dk K and Chionardes MA and Arkhaesi N | Acute acalculous cholecystitis in a pediatric dengue hemorrhagic fever patient: A case report, lesson learned from limited resource setting. | Annals of medicine and surgery (2012) | 81 |  | 104437 | eng | https://pubmed.ncbi.nlm.nih.gov/36147073/ | 10.1016/j.amsu.2022.104437 |
| TH/DZH | title/abstract | did not have primary data on US in VHF | 2022 | Siraj F and Dhar A and Shabir A and Mantoo S and Khan UH | A Rare Case of Brucellosis With Spontaneous Splenic Rupture Presenting as an Acute Abdomen. | Cureus | 14 | 9 | e28753 | eng | https://pubmed.ncbi.nlm.nih.gov/36211104/ | 10.7759/cureus.28753 |
| TH/DZH | title/abstract | did not have primary data on US in VHF | 2022 | Subaharan K and Senthamarai Selvan P and Subramanya TM and Senthoorraja R and Manjunath S and Das T and Pragadheesh VS and Bakthavatsalam N and Mohan MG and Senthil-Nathan S and Uragayala S and Samuel PP and Govindarajan R and Eswaramoorthy M | Ultrasound-assisted nanoemulsion of Trachyspermum ammi essential oil and its constituent thymol on toxicity and biochemical aspect of Aedes aegypti. | Environmental science and pollution research international | 29 | 47 | 71326-71337 | eng | https://pubmed.ncbi.nlm.nih.gov/35595904/ | 10.1007/s11356-022-20870-2 |
| TH/DZH | title/abstract | did not have primary data on US in VHF | 2022 | Tanizawa K and Handa T and Nagai S and Yokomatsu T and Ueda S and Ikezoe K and Ogino S and Hirai T and Izumi T | Basal interventricular septum thinning and long-term left ventricular function in patients with sarcoidosis. | Respiratory investigation | 60 | 3 | 385-392 | eng | https://pubmed.ncbi.nlm.nih.gov/35283057/ | 10.1016/j.resinv.2022.02.001 |
| TH/DZH | title/abstract | US in VHF, but case study or series | 2022 | Thadchanamoorthy V and Ganeshrajah A and Dayasiri K and Jayasekara NP | Acute appendicitis during the recovery phase of dengue hemorrhagic fever: two case reports. | Journal of medical case reports | 16 | 1 | 219 | eng | https://pubmed.ncbi.nlm.nih.gov/35659758/ | 10.1186/s13256-022-03443-2 |
| TH/DZH | title/abstract | did not have primary data on US in VHF | 2022 | Tian Z and Yao N and Wu Y and Wang F and Zhao Y | Serum superoxide dismutase level is a potential biomarker of disease prognosis in patients with hemorrhagic fever with renal syndrome caused by the Hantaan virus. | BMC infectious diseases | 22 | 1 | 446 | eng | https://pubmed.ncbi.nlm.nih.gov/35538453/ | 10.1186/s12879-022-07394-3 |
| TH/DZH | full text | INCLUDED: original patient level data on US in VHF | 2022 | Uthraraj NS and Sriraam LM and Hiriyur Prakash M and Kumar M and Palanisamy U and Chettiakkapalayam Venkatachalam KU | Predictive Factors for the Complications of Dengue Fever in Children: A Retrospective Analysis. | Cureus | 14 | 12 | e33027 | eng | https://pubmed.ncbi.nlm.nih.gov/36721616/ | 10.7759/cureus.33027 |
| TH/DZH | title/abstract | US in VHF, but case study or series | 2023 | √Ålvarez-Garc√≠a M and Otegui L and Ortega Larrod√© A and Calvo Hern√°ndez R | Liver enzyme alteration in response to dengue virus. | Revista espanola de enfermedades digestivas |  |  |  | eng | https://pubmed.ncbi.nlm.nih.gov/38095160/ | 10.17235/reed.2023.10102/2023 |
| TH/DZH | title/abstract | did not have primary data on US in VHF | 2023 | Anderson CA and Barrera MD and Boghdeh NA and Smith M and Alem F and Narayanan A | Brilacidin as a Broad-Spectrum Inhibitor of Enveloped, Acutely Infectious Viruses. | Microorganisms | 12 | 1 |  | eng | https://pubmed.ncbi.nlm.nih.gov/38257881/ | 10.3390/microorganisms12010054 |
| TH/DZH | title/abstract | did not have primary data on US in VHF | 2023 | Castro PT and Werner H and Araujo J√∫nior E and Bonasoni MP and Tonni G | Prenatal and Postnatal Zika Intrauterine Infection: Diagnostic Imaging Techniques and Placental Pathology. | Fetal and pediatric pathology | 42 | 2 | 207-215 | eng | https://pubmed.ncbi.nlm.nih.gov/36125260/ | 10.1080/15513815.2022.2118559 |
| TH/DZH | full text | INCLUDED: original patient level data on US in VHF | 2023 | Chaudhary S and Manrai K and Dhagat P and Dudeja P and Sen D and Grewal DS and Kakria N | Abdominal and Chest Ultrasonography: A predictor for disease progression in nonsevere dengue. | Medical journal, Armed Forces India | 79 | 4 | 386-391 | eng | https://pubmed.ncbi.nlm.nih.gov/37441301/ | 10.1016/j.mjafi.2021.03.012 |
| TH/DZH | full text | INCLUDED: original patient level data on US in VHF | 2023 | Erameh CO and Koch T and Edeawe OI and Oestereich L and Omansen T and Jochum J and Adomeh DI and Ikponmwonsa O and Aire C and Pahlmann M and Asogun DA and Ogbaini-Emovon E and Okogbenin SA and G√ºnther S and Ramharter M and Akideno PE and Kreuels B | Focussed Assessment with Sonography in acute Lassa Fever (FASLa):¬†Development of a point-of-care protocol and description of common ultrasound findings. | The Journal of infection | 87 | 1 | 27-33 | eng | https://pubmed.ncbi.nlm.nih.gov/37075910/ | 10.1016/j.jinf.2023.04.008 |
| TH/DZH | title/abstract | did not have primary data on US in VHF | 2023 | Garcia-Zamora S and Picco JM and Lepori AJ and Galello MI and Saad AK and Ay√≥n M and Monga-Aguilar N and Shehadeh I and Manganiello CF and Izaguirre C and Fallabrino LN and Clavero M and Mansur F and Ghibaudo S and Sevilla D and Cado CA and Priotti M and Liblik K and Gastaldello N and Merlo PM | Abnormal echocardiographic findings after COVID-19 infection: a multicenter registry. | The international journal of cardiovascular imaging | 39 | 1 | 77-85 | eng | https://pubmed.ncbi.nlm.nih.gov/36515755/ | 10.1007/s10554-022-02706-9 |
| TH/DZH | title/abstract | did not have primary data on US in VHF | 2023 | Inban P and Gulla V and Devani A and Akuma CM and Gowthavaram CA and Hussain A and Aung LLL and Jadav N and Savaliya B and Sajjad T and Khan AM and Akuma O and Kelechi AE | Acute pancreatitis associated with the antibiotic levofloxacin: A rare case report. | Clinical case reports | 11 | 12 | e8186 | eng | https://pubmed.ncbi.nlm.nih.gov/38033693/ | 10.1002/ccr3.8186 |
| TH/DZH | full text | ultrasound only completed in selected patients (those with ECG abnormalities) | 2023 | Jadav N and Vaghela N and Verma N and Davis D and Sonani AA and Patoliya P | Unmasking the Hidden Threat of Cardiac Involvement in Dengue Fever: A Critical Longitudinal Study. | Cureus | 15 | 9 | e45961 | eng | https://pubmed.ncbi.nlm.nih.gov/37900359/ | 10.7759/cureus.45961 |
| TH/DZH | title/abstract | did not have primary data on US in VHF | 2023 | Kaagaard MD and Matos LO and Evangelista MVP and Wegener A and Holm AE and Vestergaard LS and Do Valle SCN and Silvestre OM and Lacerda MVG and de Souza RM and Barreto Dos Santos F and Biering-S√∏rensen T and Brainin P | Frequency of pleural effusion in dengue patients by severity, age and imaging modality: a systematic review and meta-analysis. | BMC infectious diseases | 23 | 1 | 327 | eng | https://pubmed.ncbi.nlm.nih.gov/37189054/ | 10.1186/s12879-023-08311-y |
| TH/DZH | full text | INCLUDED: original patient level data on US in VHF | 2023 | Lakra MS and Karotkar SA and Lakhkar B and Dhawan V and Lakra A | To study ultrasound and chest X-ray findings and their role in the diagnosis of dengue fever in children. | Journal of family medicine and primary care | 12 | 9 | 1938-1942 | eng | https://pubmed.ncbi.nlm.nih.gov/38024929/ | 10.4103/jfmpc.jfmpc_2325_22 |
| TH/DZH | title/abstract | did not have primary data on US in VHF | 2023 | Mart√≠nez-Arias A and Valerio L and Roure-D√≠ez S and Fern√°ndez-Rivas G and Rivaya B and P√©rez-Olmeda MT and Soldevila-Langa L and Parr√≥n I and Clotet-Sala B and Vall√®s X and Rodrigo C | Zika virus screening during pregnancy: Results and lessons learned from a screening program and a post-delivery follow-up analysis (2016-2022). | Birth defects research | 115 | 17 | 1646-1657 | eng | https://pubmed.ncbi.nlm.nih.gov/37668290/ | 10.1002/bdr2.2236 |
| TH/DZH | title/abstract | did not have primary data on US in VHF | 2023 | Mathieu M and Lambert V and Carles G and Picone O and Carod JF and Pomar L and Nacher M and Hcini N | Incidence, causes, and risk factors of stillbirth in an Amazonian context: Saint Laurent du Maroni maternity ward 2016-2021. | European journal of obstetrics & gynecology and reproductive biology: X | 18 |  | 100190 | eng | https://pubmed.ncbi.nlm.nih.gov/37095766/ | 10.1016/j.eurox.2023.100190 |
| TH/DZH | title/abstract | did not have primary data on US in VHF | 2023 | Medas F and Dobrinja C and Al-Suhaimi EA and Altmeier J and Anajar S and Arikan AE and Azaryan I and Bains L and Basili G and Bolukbasi H and Bononi M and Borumandi F and Bozan MB and Brenta G and Brunaud L and Brunner M and Buemi A and Canu GL and Cappellacci F and Cartwright SB and Castells Fust√© I and Cavalheiro B and Cavallaro G and Chala A and Chan SYB and Chaplin J and Cheema MS and Chiapponi C and Chiofalo MG and Chrysos E and D'Amore A and de Cillia M and De Crea C and de Manzini N and de Matos LL and De Pasquale L and Del Rio P and Demarchi MS and Dhiwakar M and Donatini G and Dora JM and D'Orazi V and Doulatram Gamgaram VK and Eismontas V and Kabiri EH and El Malki HO and Elzahaby I and Enciu O and Eskander A and Feroci F and Figueroa-Bohorquez D and Filis D and Fran√ßois G and Fr√≠as-Fern√°ndez P and Gamboa-Dominguez A and Genc V and Giordano D and G√≥mez-Pedraza A and Graceffa G and Griffin J and Guerreiro SC and Gupta K and Gupta KK and Gurrado A and Hajiioannou J and Hakala T and Harahap WA and Hargitai L and Hartl D and Hellmann A and Hlozek J and Hoang VT and Iacobone M and Innaro N and Ioannidis O and Jang JHI and Xavier-Junior JC and Jovanovic M and Kaderli RM and Kakamad F and Kaliszewski K and Karamanliev M and Katoh H and Ko≈°ec A and Kovacevic B and Kowalski LP and Kr√°lik R and Yadav SK and Kumorov√° A and Lampridis S and Lasithiotakis K and Leclere JC and Leong EKF and Leow MK and Lim JY and Lino-Silva LS and Liu SYW and Llorach NP and Lombardi CP and L√≥pez-G√≥mez J and Lori E and Quintanilla-Dieck L and Lucchini R and Madani A and Manatakis D and Markovic I and Materazzi G and Mazeh H and Mercante G and Meyer-Rochow GY and Mihaljevic O and Miller JA and Minuto M and Monacelli M and Mulita F and Mullineris B and Mu√±oz-de-Nova JL and Murad√°s Girardi F and Nader S and Napadon T and Nastos C and Offi C and Ronen O and Oragano L and Orois A and Pan Y and Panagiotidis E and Panchangam RB and Papavramidis T and Parida PK and Paspala A and P√©rez √íV and Petrovic S and Raffaelli M and Ramacciotti CF and Ratia Gimenez T and Rivo V√°zquez √Å and Roh JL and Rossi L and Sanabria A and Santeerapharp A and Semenov A and Seneviratne S and Serdar A and Sheahan P and Sheppard SC and Slotcavage RL and Smaxwil C and Kim SY and Sorrenti S and Spartalis E and Sriphrapradang C and Testini M and Turk Y and Tzikos G and Vabalayte K and Vargas-Osorio K and V√°zquez Renter√≠a RS and Vel√°zquez-Fern√°ndez D and Vithana SMP and Y√ºcel L and Yulian ED and Zahradnikova P and Zarogoulidis P and Ziablitskaia E and Zolotoukho A and Cal√≤ PG | Effect of the COVID-19 pandemic on surgery for indeterminate thyroid nodules (THYCOVID): a retrospective, international, multicentre, cross-sectional study. | The lancet. Diabetes & endocrinology | 11 | 6 | 402-413 | eng | https://pubmed.ncbi.nlm.nih.gov/37127041/ | 10.1016/S2213-8587(23)00094-3 |
| TH/DZH | title/abstract | did not have primary data on US in VHF | 2023 | Mendpara V and Endreddy JKR and Gajula S and Ravulapalli P and Kumar M and Kaur P and Thakkar M | Overlapping Phenotypes of Alcoholic Cardiomyopathy and Left Ventricular Non-compaction: A Case Report and Discussion of Converging Cardiomyopathies. | Cureus | 15 | 11 | e48220 | eng | https://pubmed.ncbi.nlm.nih.gov/38050525/ | 10.7759/cureus.48220 |
| TH/DZH | title/abstract | did not have primary data on US in VHF | 2023 | Mi Fang H and Ng OT and Agrawal R | Myopic Shift in a Patient with Dengue Fever. | Ocular immunology and inflammation | 31 | 1 | 191-193 | eng | https://pubmed.ncbi.nlm.nih.gov/34648404/ | 10.1080/09273948.2021.1985523 |
| TH/DZH | title/abstract | did not have primary data on US in VHF | 2023 | Miller RL and Schuh H and Chandran A and Aris IM and Bendixsen C and Blossom J and Breton C and Camargo CA Jr and Canino G and Carroll KN and Commodore S and Cordero JF and Dabelea DM and Ferrara A and Fry RC and Ganiban JM and Gern JE and Gilliland FD and Gold DR and Habre R and Hare ME and Harte RN and Hartert T and Hasegawa K and Khurana Hershey GK and Jackson DJ and Joseph C and Kerver JM and Kim H and Litonjua AA and Marsit CJ and McEvoy C and Mendon√ßa EA and Moore PE and Nkoy FL and O'Connor TG and Oken E and Ownby D and Perzanowski M and Rivera-Spoljaric K and Ryan PH and Singh AM and Stanford JB and Wright RJ and Wright RO and Zanobetti A and Zoratti E and Johnson CC | Incidence rates of childhood asthma with recurrent exacerbations in the US Environmental influences on Child Health Outcomes (ECHO) program. | The Journal of allergy and clinical immunology | 152 | 1 | 84-93 | eng | https://pubmed.ncbi.nlm.nih.gov/36972767/ | 10.1016/j.jaci.2023.03.016 |
| TH/DZH | title/abstract | did not have primary data on US in VHF | 2023 | Montero-V√°zquez RA | [Kawasaki disease cytokine release syndrome and Kawasaki disease shock syndrome: A case report]. | Revista alergia Mexico (Tecamachalco, Puebla, Mexico : 1993) | 69 | 3 | 146-150 | spa | https://pubmed.ncbi.nlm.nih.gov/36869015/ | 10.29262/ram.v69i3.1152 |
| TH/DZH | title/abstract | US in VHF, but case study or series | 2023 | Munasinghe KVP and Silva FHDS | A Case Report of Takotsubo Cardiomyopathy With Dengue. | Cureus | 15 | 7 | e42774 | eng | https://pubmed.ncbi.nlm.nih.gov/37663978/ | 10.7759/cureus.42774 |
| TH/DZH | full text | no patient level ultrasound data | 2023 | Nainggolan L and Dewi BE and Hakiki A and Pranata AJ and Sudiro TM and Martina B and van Gorp E | Association of viral kinetics, infection history, NS1 protein with plasma leakage among Indonesian dengue infected patients. | PloS one | 18 | 5 | e0285087 | eng | https://pubmed.ncbi.nlm.nih.gov/37130105/ | 10.1371/journal.pone.0285087 |
| TH/DZH | full text | cases not confirmed | 2023 | Osorio L and Prieto I and Zuluaga D and Ropero D and Dewan N and Kirsch JD | Evaluation of remote radiologist-interpreted point-of-care ultrasound for suspected dengue patients in a primary health care facility in Colombia. | Infectious diseases of poverty | 12 | 1 | 90 | eng | https://pubmed.ncbi.nlm.nih.gov/37759280/ | 10.1186/s40249-023-01141-9 |
| TH/DZH | title/abstract | US in VHF, but case study or series | 2023 | Pahari S and Basukala S and Kunwar P and Thapa K and Khand Y and Thapa O | Spontaneous splenic hematoma secondary to dengue infection: a rare case report. | Annals of medicine and surgery (2012) | 85 | 4 | 1030-1033 | eng | https://pubmed.ncbi.nlm.nih.gov/37113826/ | 10.1097/MS9.0000000000000312 |
| TH/DZH | title/abstract | did not have primary data on US in VHF | 2023 | Patil C and Bandaru A and Bandari R and Kumar M and Kumar P | Long-Segment Epidural Hemorrhage of the Cervical and Dorsal Spine: A Case Report of a Rare Complication of Dengue Virus Disease. | Cureus | 15 | 1 | e33435 | eng | https://pubmed.ncbi.nlm.nih.gov/36751224/ | 10.7759/cureus.33435 |
| TH/DZH | title/abstract | US in VHF, but case study or series | 2023 | Pradeep C and Karunathilake P and Abeyagunawardena S and Ralapanawa U and Jayalath T | Hemophagocytic lymphohistiocytosis as a rare complication of dengue haemorrhagic fever: a case report. | Journal of medical case reports | 17 | 1 | 224 | eng | https://pubmed.ncbi.nlm.nih.gov/37259116/ | 10.1186/s13256-023-03967-1 |
| TH/DZH | full text | no patient level ultrasound data | 2023 | Rezende IM and McClure MA and Pereira LS and Fradico JRB and Cenachi ARC and Moura AS and Paladino LLA and Dutra MRT and Alves PA and Xavier MAP and Said RFDC and Ramalho DB and Gama TDP and Martins-Filho OA and Monath TP and Teixeira-Carvalho A and Drumond BP and LaBeaud AD | Characterization and Investigation of Risk Factors for Late-Relapsing Hepatitis After Yellow Fever. | Clinical infectious diseases : an official publication of the Infectious Diseases Society of America | 77 | 4 | 565-573 | eng | https://pubmed.ncbi.nlm.nih.gov/37099356/ | 10.1093/cid/ciad249 |
| TH/DZH | full text | INCLUDED: original patient level data on US in VHF | 2023 | Saghir H and Tariq M and Safdar N and Zahoor A and Rashid F and Khan SE and Abid S and Mumtaz H | Association of Liver Function Test with Severity of Dengue Fever in Suburbs of Islamabad. | Journal of community hospital internal medicine perspectives | 13 | 6 | 95-99 | eng | https://pubmed.ncbi.nlm.nih.gov/38596542/ | 10.55729/2000-9666.1250 |
| TH/DZH | title/abstract | did not have primary data on US in VHF | 2023 | Saini SS and Copello GJ and Martini MF | Solid phase extraction with rotating cigarette filter for determination of bisphenol A in source and drinking water: computational and analytical studies. | Analytical sciences : the international journal of the Japan Society for Analytical Chemistry | 39 | 4 | 607-617 | eng | https://pubmed.ncbi.nlm.nih.gov/36807887/ | 10.1007/s44211-023-00276-x |
| TH/DZH | title/abstract | did not have primary data on US in VHF | 2023 | Sandeep M and Padhi BK and Yella SST and Sruthi KG and Venkatesan RG and Krishna Sasanka KBS and Satapathy P and Mohanty A and Al-Tawfiq JA and Iqhrammullah M and Rabaan AA and Kabi A and Sah S and Rustagi S and Al-Qaim ZH and Barboza JJ and Waheed Y and Harapan H and Sah R | Myocarditis manifestations in dengue cases: A systematic review and meta-analysis. | Journal of infection and public health | 16 | 11 | 1761-1768 | eng | https://pubmed.ncbi.nlm.nih.gov/37738692/ | 10.1016/j.jiph.2023.08.005 |
| TH/DZH | title/abstract | did not have primary data on US in VHF | 2023 | Santini M and Ljubiƒá J and ≈†o≈°tar N and Vilibiƒá-ƒåavlek T and Bogdaniƒá M and Zakotnik S and Av≈°iƒç-≈Ωupanc T and Korva M and Kurolt IC and Radmaniƒá L and ≈†imiƒçiƒá P and Krznariƒá J and Gjura≈°in B and Kutle≈°a M and Vi≈°koviƒá K and Balent NC and ≈Ωunec R and Margeta Mariƒá I and Ribaroviƒá A and ≈Ωidovec-Lepej S | Hantavirus Pulmonary Syndrome Caused by Puumala Orthohantavirus-A Case Report and Literature Review. | Microorganisms | 11 | 12 |  | eng | https://pubmed.ncbi.nlm.nih.gov/38138107/ | 10.3390/microorganisms11122963 |
| TH/DZH | title/abstract | did not have primary data on US in VHF | 2023 | Saron WAA and Shanmugam K and Tung CC and Patmanathan RK and Rathore APS and Anderson DE and St John AL | Exacerbated Zika virus-induced neuropathology and microcephaly in fetuses of dengue-immune nonhuman primates. | Science translational medicine | 15 | 719 | eadd2420 | eng | https://pubmed.ncbi.nlm.nih.gov/37878671/ | 10.1126/scitranslmed.add2420 |
| TH/DZH | title/abstract | did not have primary data on US in VHF | 2023 | Singh D and Kaur L | Maternal Zika Virus Infection in the First Trimester and Fetal Stigmata in the Third Trimester. | The Indian journal of radiology & imaging | 33 | 3 | 400-402 | eng | https://pubmed.ncbi.nlm.nih.gov/37362378/ | 10.1055/s-0043-1761254 |
| TH/DZH | title/abstract | did not have primary data on US in VHF | 2023 | Singh S and Inban P and Mishra A and Yadav AS and Singh T and Singh R and Savaliya BP and Mankad SP and Gowthavaram CA and Khan A | Atypical Kawasaki Disease in a 16-Month-Old Baby: A Case Report and Literature Review. | Cureus | 15 | 5 | e39336 | eng | https://pubmed.ncbi.nlm.nih.gov/37378132/ | 10.7759/cureus.39336 |
| TH/DZH | title/abstract | did not have primary data on US in VHF | 2023 | Sinha P and Chauhan VS | Rectus Sheath Hematoma in a Recovering Dengue Patient: A Case Report. | Cureus | 15 | 11 | e49338 | eng | https://pubmed.ncbi.nlm.nih.gov/38143708/ | 10.7759/cureus.49338 |
| TH/DZH | title/abstract | did not have primary data on US in VHF | 2023 | Timilsina B and Suryabanshi A and Bhandari A and Pokhrel S and Chataut CP | Spontaneous widespread muscle hematoma complicated by pyomyositis in a case of dengue hemorrhagic fever: a case report from Nepal. | Annals of medicine and surgery (2012) | 85 | 10 | 5223-5227 | eng | https://pubmed.ncbi.nlm.nih.gov/37811049/ | 10.1097/MS9.0000000000001222 |
| TH/DZH | full text | no patient level ultrasound data | 2023 | Yang Y and Lian J and Zhao Y and Huo C and Lv S and Gao H and Wang Z | Lung ultrasonography versus chest CT for assessing peripheric pulmonary lesions in hemorrhagic fever with renal syndrome: a prospective comparative study in China. | European radiology | 33 | 7 | 4895-4904 | eng | https://pubmed.ncbi.nlm.nih.gov/36840765/ | 10.1007/s00330-023-09457-8 |
| TH/DZH | title/abstract | did not have primary data on US in VHF | 2023 | Zakharyan EA and Gritskevich OY and Ibragimova RE and Grigoriev PE | Correlation of Serum Endocan Level With Apoptosis Indicators and Severity of Atherosclerotic Lesions of Coronary Arteries in Patients With Coronary Heart Disease. | Kardiologiia | 63 | 11 | Dec-20 | ["rus", "eng"] | https://pubmed.ncbi.nlm.nih.gov/38088108/ | 10.18087/cardio.2023.11.n2570 |
| TH/DZH | title/abstract | did not have primary data on US in VHF | 2023 | Zullo F and Di Mascio D and Berghella V | Evidence-based labor management: postpartum care after vaginal delivery (part 6). | American journal of obstetrics & gynecology MFM | 5 | 7 | 100977 | eng | https://pubmed.ncbi.nlm.nih.gov/37094636/ | 10.1016/j.ajogmf.2023.100977 |
| TH/DZH | title/abstract | US in VHF, but case study or series | 2024 | √Ålvarez-Garc√≠a M and Otegui L and Ortega Larrod√© A and Calvo Hern√°ndez R | Liver enzyme alteration in response to dengue virus. | Revista espanola de enfermedades digestivas | 116 | 12 | 731 | eng | https://pubmed.ncbi.nlm.nih.gov/38095160/ | 10.17235/reed.2023.10102/2023 |
| TH/DZH | title/abstract | did not have primary data on US in VHF | 2024 | Ali Naghian AH and Hashemi Z and Chekin F and Saleh N | An ordered mesoporous carbon-silica hybrid for the detection of the antiviral drug ribavirin in clinical samples. | Nanoscale advances | 6 | 18 | 4657-4663 | eng | https://pubmed.ncbi.nlm.nih.gov/39263401/ | 10.1039/d4na00435c |
| TH/DZH | title/abstract | did not have primary data on US in VHF | 2024 | Bravo-Jaimes K and Vasquez-Loarte T and Rojas-Camayo J and Medina M and Mejia CR and Zapata-Galarza H and Berrocal M and Orozco J and Lozano D and Santiva√±ez M and Sangay C and Rosales W and Mamani L and Macedo N and Coronado J and Huaman G and Marquez R and Li Z and Masood KM and Alejos J and Reardon L and Lin J | A new algorithm DEtectS critical Congenital Heart Disease at different altitudes: ANDES-CHD study. | Journal of perinatology : official journal of the California Perinatal Association | 44 | 3 | 373-378 | eng | https://pubmed.ncbi.nlm.nih.gov/38308011/ | 10.1038/s41372-024-01888-5 |
| TH/DZH | title/abstract | did not have primary data on US in VHF | 2024 | Campana V and Inizan C and Pommier JD and Menudier LY and Vincent M and Lecuit M and Lamballerie X and Dupont-Rouzeyrol M and Murgue B and Cabi√© A | Liver involvement in dengue: A systematic review. | Reviews in medical virology | 34 | 4 | e2564 | eng | https://pubmed.ncbi.nlm.nih.gov/38923215/ | 10.1002/rmv.2564 |
| TH/DZH | title/abstract | did not have primary data on US in VHF | 2024 | de Oliveira Neto EG and Nascimento DDL and Bertoncini TV and Lopes AA and Bezerra ASA and Soares MVA | The role of the radiologist in the dengue endemic: a pictorial essay. | Radiologia brasileira | 57 |  | e20240077en | eng | https://pubmed.ncbi.nlm.nih.gov/39687468/ | 10.1590/0100-3984.2024.0077-en |
| TH/DZH | title/abstract | US in VHF, but case study or series | 2024 | Dom√≠nguez-Rojas J and Cabrera-Rojas L and Prado-G√≥mez T and Atamari-Anahui N | [Fulminant acute liver failure secondary to severe dengue in a child: case report]. | Revista de gastroenterologia del Peru : organo oficial de la Sociedad de Gastroenterologia del Peru | 44 | 2 | 150-154 | spa | https://pubmed.ncbi.nlm.nih.gov/39019809/ |  |
| TH/DZH | title/abstract | did not have primary data on US in VHF | 2024 | Galeano MA and Ranalletti ML and Pelizzari M and Sabbione L and Lutereau JF and Salinas APS and Anoni MC and Gonseski VC | Contribution of imaging in the diagnosis of three neglected diseases in the Southern Cone: Leishmaniasis, Dengue, and Chikungunya. | Pediatric radiology |  |  |  | eng | https://pubmed.ncbi.nlm.nih.gov/39107472/ | 10.1007/s00247-024-06016-1 |
| TH/DZH | title/abstract | did not have primary data on US in VHF | 2024 | GBD 2021 Stroke Risk Factor Collaborators | Global, regional, and national burden of stroke and its risk factors, 1990-2021: a systematic analysis for the Global Burden of Disease Study 2021. | The Lancet. Neurology | 23 | 10 | 973-1003 | eng | https://pubmed.ncbi.nlm.nih.gov/39304265/ | 10.1016/S1474-4422(24)00369-7 |
| TH/DZH | title/abstract | did not have primary data on US in VHF | 2024 | Hcini N and Lambert V and Carod JF and Mathieu M and Carles G and Picone O and Sibiude J and Pomar L and Nacher M | Emerging and re-emerging infectious diseases in pregnant women in an amazonian region: a large retrospective study from French Guiana. | European journal of clinical microbiology & infectious diseases : official publication of the European Society of Clinical Microbiology | 43 | 6 | 1081-1090 | eng | https://pubmed.ncbi.nlm.nih.gov/38573394/ | 10.1007/s10096-024-04813-y |
| TH/DZH | title/abstract | did not have primary data on US in VHF | 2024 | Hu H and Zhan J and Chen W and Yang Y and Jiang H and Zheng X and Li J and Hu F and Yu D and Li J and Yang X and Zhang Y and Wang X and Bi Z and Liang Y and Shen H and Du H and Lian J | Development and validation of a novel death risk stratification scale in patients with hemorrhagic fever with renal syndrome: a 14-year ambispective cohort study. | Clinical microbiology and infection : the official publication of the European Society of Clinical Microbiology and Infectious Diseases | 30 | 3 | 387-394 | eng | https://pubmed.ncbi.nlm.nih.gov/37952580/ | 10.1016/j.cmi.2023.11.003 |
| TH/DZH | full text | unclear if cases confirmed | 2024 | Jain A and Vasnaik M and Singam A and Mudiganti VNKS | A Cross-Sectional Study on Bedside Abdominal Ultrasound Findings as a Diagnostic and Prognostic Tool in Dengue Fever in Manipal Hospital, Bengaluru, India. | Cureus | 16 | 7 | e64734 | eng | https://pubmed.ncbi.nlm.nih.gov/39156261/ | 10.7759/cureus.64734 |
| TH/DZH | title/abstract | did not have primary data on US in VHF | 2024 | Jean Pierre AR and Green SR and Anandaraj L and Sivaprakasam M and Kasirajan A and Devaraju P and Anumulapuri S and Mutheneni SR and Balakrishna Pillai A | Severity prediction markers in dengue: a prospective cohort study using machine learning approach. | Biomarkers : biochemical indicators of exposure, response, and susceptibility to chemicals | 29 | 8 | 557-564 | eng | https://pubmed.ncbi.nlm.nih.gov/39556525/ | 10.1080/1354750X.2024.2430997 |
| TH/DZH | title/abstract | US in VHF, but case study or series | 2024 | Kashyap A and Bhatta LR and Bhatta B and Nepali P and Gyawali P and Rajak A and Dhakal S | Dengue fever complicated with ST elevation myocardial infarction with atypical features: A case report. | SAGE open medical case reports | 12 |  | 2050313X231225342 | eng | https://pubmed.ncbi.nlm.nih.gov/38205141/ | 10.1177/2050313X231225342 |
| TH/DZH | full text | no patient level ultrasound data | 2024 | Keshav LB and K A and Malhotra K and Shetty S | Lung Manifestation of Dengue Fever: A Retrospective Study. | Cureus | 16 | 5 | e60655 | eng | https://pubmed.ncbi.nlm.nih.gov/38903312/ | 10.7759/cureus.60655 |
| TH/DZH | title/abstract | US in VHF, but case study or series | 2024 | Khadka B and Khanal K and Regmi A and Ghimire A and Kc S and Nepal R | Pyopericardium progressing to cardiac tamponade in a patient with dengue fever. | IDCases | 36 |  | e01996 | eng | https://pubmed.ncbi.nlm.nih.gov/38873641/ | 10.1016/j.idcr.2024.e01996 |
| TH/DZH | title/abstract | did not have primary data on US in VHF | 2024 | Lau MJ and Valdez AR and Jones MJ and Aranson I and Hoffmann AA and McGraw EA | The effect of repeat feeding on dengue virus transmission potential in Wolbachia-infected Aedes aegypti following extended egg quiescence. | PLoS neglected tropical diseases | 18 | 7 | e0012305 | eng | https://pubmed.ncbi.nlm.nih.gov/38976758/ | 10.1371/journal.pntd.0012305 |
| TH/DZH | full text | no patient level ultrasound data | 2024 | McBride A and Duyen HTL and Vuong NL and Tho PV and Tai LTH and Phong NT and Ngoc NT and Yen LM and Nhat PTH and Vi TT and Llewelyn MJ and Thwaites L and Hao NV and Yacoub S | Endothelial and inflammatory pathophysiology in dengue shock: New insights from a prospective cohort study in Vietnam. | PLoS neglected tropical diseases | 18 | 3 | e0012071 | eng | https://pubmed.ncbi.nlm.nih.gov/38536887/ | 10.1371/journal.pntd.0012071 |
| TH/DZH | title/abstract | US in VHF, but case study or series | 2024 | Mishra AK and Mateen S and Jabeen F and Singh S and Verma PK | Sheehan Syndrome Unmasked by Adrenal Crisis Secondary to Severe Dengue Fever. | Ochsner journal | 24 | 3 | 219-224 | eng | https://pubmed.ncbi.nlm.nih.gov/39280878/ | 10.31486/toj.24.0019 |
| TH/DZH | title/abstract | did not have primary data on US in VHF | 2024 | Osolodkin DI and Kozlovskaya LI and Iusupov IR and Kurkin AV and Shustova EY and Orlov AA and Khvatov EV and Mutnykh ES and Kurashova SS and Vetrova AN and Yatsenko DO and Goryashchenko AS and Ivanov VN and Lukyanenko ER and Karpova EV and Stepanova DA and Volok VP and Sotskova SE and Dzagurova TK and Karganova GG and Lukashev AN and Ishmukhametov AA | Phenotypic assessment of antiviral activity for spiro-annulated oxepanes and azepenes. | Chemical biology & drug design | 103 | 5 | e14553 | eng | https://pubmed.ncbi.nlm.nih.gov/38789394/ | 10.1111/cbdd.14553 |
| TH/DZH | title/abstract | US in VHF, but case study or series | 2024 | Pramudyo M and Putra ICS and Iqbal M and Prameswari HS and Karwiky G and Dewi TI and Raharjo P and Kamarullah W and Sukmadi N | Clinically suspected acute right ventricular fulminant dengue myocarditis masquerading with dual lethal arrhythmias: a case report. | Journal of medical case reports | 18 | 1 | 554 | eng | https://pubmed.ncbi.nlm.nih.gov/39548493/ | 10.1186/s13256-024-04792-w |
| TH/DZH | title/abstract | did not have primary data on US in VHF | 2024 | Qi F and Cao Y and Shen Y and Wang H and Li D and Yang Q and Li Z and Zhang Z | Nasopharyngeal neutrophilic-retention signatures could predict disease progression in early SARS-CoV-2 infection. | Journal of medical virology | 96 | 1 | e29328 | eng | https://pubmed.ncbi.nlm.nih.gov/38146903/ | 10.1002/jmv.29328 |
| TH/DZH | title/abstract | US in VHF, but case study or series | 2024 | Sa√∫l P and Lamberto Y and Chac√≥n N and S√°nchez Cunto M and Chediack V and Cunto E | [Takotsubo syndrome in dengue patient]. | Medicina | 84 | 3 | 584-587 | spa | https://pubmed.ncbi.nlm.nih.gov/38907979/ |  |
| TH/DZH | full text | no patient level ultrasound data | 2024 | Vo LT and Nguyen DT and Tran TN and Tran HH and ƒêoan TT and Pham TN and Mai TT and Nguyen QX and Nguyen TK and Nguyen TT and Burza S and Nguyen TT | Pediatric Profound Dengue Shock Syndrome and Use of Point-of-Care Ultrasound During Mechanical Ventilation to Guide Treatment: Single-Center Retrospective Study, 2013-2021. | Pediatric critical care medicine : a journal of the Society of Critical Care Medicine and the World Federation of Pediatric Intensive and Critical Care Societies | 25 | 4 | e177-e185 | eng | https://pubmed.ncbi.nlm.nih.gov/37966344/ | 10.1097/PCC.0000000000003413 |
| TH/DZH | title/abstract | did not have primary data on US in VHF | 2024 | Yang X and Yu C and Chen Y and Nian B and Chai M and Maimaiti D and Xu D and Zang X | Hemorrhagic Fever with Renal Syndrome Complicated by Acute Pancreatitis, High Intraocular Pressure, and Pulmonary Involvement: a Case Report. | Infection and drug resistance | 17 |  | 1919-1925 | eng | https://pubmed.ncbi.nlm.nih.gov/38766677/ | 10.2147/IDR.S454049 |
| TH/DZH | title/abstract | did not have primary data on US in VHF | 2024 | Zenchenko AA and Drenichev MS and Khvatov EV and Uvarova VI and Goryashchenko AS and Frolenko VS and Karpova EV and Kozlovskaya LI and Osolodkin DI and Ishmukhametov AA and Mikhailov SN and Oslovsky VE | Elongation of N(6)-benzyladenosine scaffold via Pd-catalyzed C-C bond formation leads to derivatives with antiflaviviral activity. | Bioorganic & medicinal chemistry | 98 |  | 117552 | eng | https://pubmed.ncbi.nlm.nih.gov/38128296/ | 10.1016/j.bmc.2023.117552 |
| TH/DZH | title/abstract | US in VHF, but case study or series | 2025 | Abshir T and Al Hassani A and Al Hassani Z and Saleem A | Dengue Hemorrhagic Fever Complicated by Complete Atrioventricular Block: A Case Report. | Cureus | 17 | 5 | e84611 | eng | https://pubmed.ncbi.nlm.nih.gov/40546620/ | 10.7759/cureus.84611 |
| TH/DZH | title/abstract | did not have primary data on US in VHF | 2025 | Chernorotov V A and Kostenich V S and Eremenko A N | [Fat necrosis mimicking recurrent kidney cancer]. | Urologiia (Moscow, Russia : 1999) |  | 1 | 75-78 | rus | https://pubmed.ncbi.nlm.nih.gov/40377639/ |  |
| TH/DZH | title/abstract | did not have primary data on US in VHF | 2025 | Galeano MA and Ranalletti ML and Pelizzari M and Sabbione L and Lutereau JF and Salinas APS and Anoni MC and Gonseski VC | Contribution of imaging in the diagnosis of three neglected diseases in the Southern Cone: Leishmaniasis, Dengue, and Chikungunya. | Pediatric radiology | 55 | 1 | 75-87 | eng | https://pubmed.ncbi.nlm.nih.gov/39107472/ | 10.1007/s00247-024-06016-1 |
| TH/DZH | title/abstract | did not have primary data on US in VHF | 2025 | Ignatiev VV and Oleynik AV and Muraviev AV and Garapov TA and Dankova II and Tishukov MY | [Treatment of children with blunt spleen injury throughout 25-year period in Sevastopol and the Republic of Crimea]. | Khirurgiia |  | 2 | 67-76 | rus | https://pubmed.ncbi.nlm.nih.gov/39918805/ | 10.17116/hirurgia202502167 |
| TH/DZH | title/abstract | did not have primary data on US in VHF | 2025 | Imani-Musimwa P and Grant E and Mukadi-Bamuleka D and Tsongo-Kibendelwa Z and Fraterne-Muhayangabo R and Mbala-Kingebeni P and Kitenge-Omasumbu R and Nyakio-Ngeleza O and Barhwamire-Kabesha T and Minani-Ndabahweje M and Sihali-Kyolov J and Bitwe-Mihanda R and Sengeyi-Mushengezi-Amani D and Ververs M | Maternal and fetal survival following Ebola, HIV and Malaria co-infection in the first trimester of gestation in resource-limited setting in Democratic Republic of Congo. | BMC pregnancy and childbirth | 25 | 1 | 210 | eng | https://pubmed.ncbi.nlm.nih.gov/40011840/ | 10.1186/s12884-025-07265-0 |
| TH/DZH | title/abstract | did not have primary data on US in VHF | 2025 | Jayawardena TU and Merindol N and Liyanage NS and G√©linas SE and Lionel B and Seydou K and Seck M and Evidente A and Desgagn√©-Penix I | Antiviral alkaloids from Crinum jagus: Extraction, synergistic effects, and activity against dengue virus and human coronavirus OC43. | Heliyon | 11 | 4 | e42580 | eng | https://pubmed.ncbi.nlm.nih.gov/40028517/ | 10.1016/j.heliyon.2025.e42580 |
| TH/DZH | title/abstract | US in VHF, but case study or series | 2025 | Kasuni Suwandika WA and Senadeera VR and Ranga CKVD and Chathuranga WAT and Warushahennadi J and Dissanayake AS and Fonseka CL | Dying From Dengue Encephalitis in the Absence of DHF: A Case Report. | Case reports in infectious diseases | 2025 |  | 9852545 | eng | https://pubmed.ncbi.nlm.nih.gov/41122205/ | 10.1155/crdi/9852545 |
| TH/DZH | title/abstract | US in VHF, but case study or series | 2025 | Kolapo AR and Johnson BO | Acute Appendicitis Occurring in Dengue Fever: A Case Report and Review of the Literature. | Case reports in infectious diseases | 2025 |  | 4654968 | eng | https://pubmed.ncbi.nlm.nih.gov/40727843/ | 10.1155/crdi/4654968 |
| TH/DZH | title/abstract | did not have primary data on US in VHF | 2025 | Munawar F and Rana IA and Mumtaz MA | PDA-associated infective endocarditis with pulmonary artery perforation. | Pakistan journal of medical sciences | 41 | 1 | 344-346 | eng | https://pubmed.ncbi.nlm.nih.gov/39867805/ | 10.12669/pjms.41.1.10200 |
| TH/DZH | title/abstract | did not have primary data on US in VHF | 2025 | Nicacio JM and de Souza CDF and Khouri R and Pereira VC and do Carmo RF and Patriota PVAM and Nunes SLP and de Morais J√∫nior JC and Barral-Netto M and Lima JAC and Armstrong ADC | Arbovirus exposure and subclinical myocardial dysfunction in an Indigenous population in Northeast Brazil: a cross-sectional study. | BMC infectious diseases | 25 | 1 | 89 | eng | https://pubmed.ncbi.nlm.nih.gov/39833670/ | 10.1186/s12879-024-10320-4 |
| TH/DZH | title/abstract | US in VHF, but case study or series | 2025 | Parepalli A and Saboo K and Acharya S and Kota V and Nelakuditi M | Dengue Hemorrhagic Fever Presenting With Pericardial Effusion and Cardiac Tamponade: A Case Report. | Cureus | 17 | 1 | e76836 | eng | https://pubmed.ncbi.nlm.nih.gov/39897308/ | 10.7759/cureus.76836 |
| TH/DZH | title/abstract | did not have primary data on US in VHF | 2025 | Salloum M and Larivi√®re Y and Bikioli Bolombo F and Zola Matuvanga T and Lemey G and Maketa V and Muhindo-Mavoko H and Van Damme P and Mitashi P and Bastiaens H and Van Geertruyden JP and Paviotti A | The exclusion of pregnant women from an Ebola vaccine trial in Boende, western DRC: Perceptions of female participants who became pregnant and community members. | Vaccine | 55 |  | 127000 | eng | https://pubmed.ncbi.nlm.nih.gov/40121733/ | 10.1016/j.vaccine.2025.127000 |
| TH/DZH | title/abstract | did not have primary data on US in VHF | 2025 | Shahsavand Davoudi A and Harandi H and Samiee R and Forghani S and Mohammadi K and Shafaati M | Ultrasound evaluation of gallbladder wall thickness for predicting severe dengue: a systematic review and meta-analysis. | The ultrasound journal | 17 | 1 | 12 | eng | https://pubmed.ncbi.nlm.nih.gov/39899203/ | 10.1186/s13089-025-00417-5 |
| TH/DZH | title/abstract | did not have primary data on US in VHF | 2025 | Thockchom N and Sharma R and Panda PK and Gupta AK | Dengue fever complicated with bilateral Psoas hematoma and superadded infection: A case report. | Journal of vector borne diseases | 62 | 2 | 242-247 | eng | https://pubmed.ncbi.nlm.nih.gov/39836045/ | 10.4103/JVBD.JVBD_193_23 |
| TH/DZH | title/abstract | did not have primary data on US in VHF | 2025 | Trujillo-Aguirre ML and Marcilla-Truyenque RL and Quiroz-Aldave JE and Morales-Moreno A and Durand-V√°squez MDC and Concepci√≥n-Zavaleta MJ and Paz-Ibarra J | Post-dengue subacute thyroiditis in a Peruvian woman: case report and literature review. | Revista peruana de medicina experimental y salud publica | 41 | 4 | 431-437 | ["spa", "eng"] | https://pubmed.ncbi.nlm.nih.gov/39936768/ | 10.17843/rpmesp.2024.414.14228 |
