# Supplementary material for: A systematic review of ultrasound to predict severe disease in patients with dengue and other viral infections associated with hemorrhagic fevers
Source: PLoS Negl Trop Dis. 2026 Sep 17;20(9):e0014609. doi: 10.1371/journal.pntd.0014609 (PMC13585234; doi:10.1371/journal.pntd.0014609)
Supplement: Supplementary file 6 [file pntd.0014609.s006.docx]

Supporting Information 6. Sensitivity and specificity with weights from the meta-analysis of ultrasound findings for severe disease. SROC plots created in MetaDTA.^1,2^ For hantavirus, only a single study testing a single ultrasound finding was included, so SROC plots could not be created.

6a. Dengue.

| Author | Finding | True  positive | False negative | False positive | True  negative | Sensitivity | Specificity | Weight  Sensitivity | Weight  Specificity |
| --- | --- | --- | --- | --- | --- | --- | --- | --- | --- |
| Srikiatkhachorn A 2007 | Ascites | 3.0 | 18.0 | 4.0 | 57.0 | 0.143 | 0.934 | 8.351 | 8.283 |
| Donaldson CD 2021 | Ascites | 10.1 | 60.9 | 0.1 | 106.9 | 0.142 | 0.999 | 7.371 | 8.368 |
| Sigera PC 2021 | Ascites | 19.3 | 0.7 | 108.3 | 299.7 | 0.965 | 0.735 | 10.972 | 8.647 |
| Srikiatkhachorn A 2007 | GBWT | 1.0 | 20.0 | 3.0 | 58.0 | 0.048 | 0.951 | 7.017 | 8.096 |
| Parmar J 2019 | GBWT | 60.0 | 24.0 | 72.0 | 88.0 | 0.714 | 0.550 | 11.899 | 8.588 |
| Donaldson CD 2021 | GBWT | 29.0 | 41.0 | 5.0 | 101.0 | 0.414 | 0.953 | 10.81 | 8.552 |
| Donaldson CD 2021 | Pericardial effusion | 5.0 | 66.0 | 0.0 | 107.0 | 0.071 | 1.000 | 5.917 | 8.126 |
| Mansanguan C 2021 | Pericardial effusion | 6.1 | 36.9 | 0.1 | 39.9 | 0.141 | 0.998 | 6.794 | 7.845 |
| Srikiatkhachorn A 2007 | Pleural effusion | 2.0 | 19.0 | 1.0 | 60.0 | 0.095 | 0.984 | 5.053 | 8.066 |
| Donaldson CD 2021 | Pleural effusion | 13.0 | 57.0 | 3.0 | 103.0 | 0.186 | 0.972 | 9.818 | 8.455 |
| Sigera PC 2021 | Pleural effusion | 10.0 | 9.0 | 19.0 | 388.0 | 0.526 | 0.953 | 8.25 | 8.689 |
| Donaldson CD 2021 | Splenomegaly | 7.0 | 63.0 | 1.0 | 105.0 | 0.100 | 0.991 | 7.748 | 8.285 |

6b. Crimean-Congo hemorrhagic fever.

| Author | Finding | True  positive | False  negative | False  positive | True  negative | Sensitivity | Specificity | Weight  Sensitivity | Weight  Specificity |
| --- | --- | --- | --- | --- | --- | --- | --- | --- | --- |
| Engin A 2009 | pericardial effusion | 5.4 | 0.6 | 14.4 | 25.6 | 0.905 | 0.639 | 12.123 | 10.289 |
| Osquee HO 2020 | pleural effusion | 0.1 | 7.9 | 11.1 | 126.9 | 0.010 | 0.920 | 8.001 | 10.460 |
| Ziraman I 2014 | ascites | 7.0 | 11.0 | 16.0 | 176.0 | 0.389 | 0.917 | 12.741 | 10.591 |
| Karavas E 2019 | ascites | 0.0 | 11.0 | 1.0 | 38.0 | 0.002 | 0.974 | 6.382 | 8.838 |
| Osquee HO 2020 | ascites | 7.3 | 0.7 | 38.3 | 99.7 | 0.914 | 0.722 | 12.862 | 10.547 |
| Ziraman I 2014 | GBWT | 11.0 | 7.0 | 33.0 | 159.0 | 0.611 | 0.828 | 14.610 | 10.595 |
| Karavas E 2019 | GBWT | 0.0 | 10.0 | 0.0 | 38.0 | 0.001 | 1.000 | 4.563 | 7.394 |
| Osquee HO 2020 | GBWT | 3.0 | 4.0 | 46.0 | 91.0 | 0.429 | 0.664 | 12.537 | 10.580 |
| Ziraman I 2014 | mesenteric lymphadenopathy | 0.0 | 19.0 | 7.0 | 186.0 | 0.002 | 0.964 | 8.951 | 10.198 |
| Osquee HO 2020 | hyperechoic kidneys | 1.0 | 6.0 | 9.0 | 128.0 | 0.143 | 0.934 | 7.230 | 10.509 |

6c. Lassa virus.

| Author | Finding | True  positive | False  negative | False  positive | True  negative | Sensitivity | Specificity | Weight  Sensitivity | Weight  Specificity |
| --- | --- | --- | --- | --- | --- | --- | --- | --- | --- |
| Erameh CO 2023 | ascites | 1.0 | 6.0 | 13.0 | 24.0 | 0.143 | 0.649 | 30.190 | 17.865 |
| Erameh CO 2023 | pleural effusion | 3.0 | 2.0 | 2.0 | 27.0 | 0.600 | 0.931 | 11.390 | 20.577 |
| Erameh CO 2023 | pericardial effusion | 1.0 | 6.0 | 9.0 | 30.0 | 0.143 | 0.769 | 26.427 | 19.768 |
| Erameh CO 2023 | kidneys | 4.0 | 3.0 | 4.0 | 35.0 | 0.571 | 0.897 | 16.347 | 21.520 |
| Erameh CO 2023 | B lines | 0.1 | 4.9 | 2.1 | 28.9 | 0.012 | 0.934 | 15.645 | 20.271 |

6d. Yellow fever.

| Author | Finding | True  positive | False  negative | False  positive | True  negative | Sensitivity | Specificity | Weight  Sensitivity | Weight  Specificity |
| --- | --- | --- | --- | --- | --- | --- | --- | --- | --- |
| Paixao GMM 2019 | pericardial effusion | 9.0 | 39.0 | 1.0 | 21.0 | 0.188 | 0.955 | 32.657 | 33.090 |
| Neves YCS 2021 | ascites | 9.0 | 17.0 | 5.0 | 15.0 | 0.346 | 0.750 | 33.412 | 34.587 |
| Neves YCS 2021 | GBWT | 24.0 | 4.0 | 15.0 | 5.0 | 0.857 | 0.250 | 33.931 | 32.324 |

**References**

1.	Freeman SC, Kerby CR, Patel A, Cooper NJ, Quinn T, Sutton AJ. Development of an interactive web-based tool to conduct and interrogate meta-analysis of diagnostic test accuracy studies: MetaDTA. *BMC Med Res Methodol* 2019; **19**: 81.

2.	Patel A, Cooper N, Freeman S, Sutton A. Graphical enhancements to summary receiver operating characteristic plots to facilitate the analysis and reporting of meta-analysis of diagnostic test accuracy data. *Res Synth Methods* 2021; **12**: 34–44.
